# Supplementary figures and images for: USP25 aggravates liver cancer development and impairs chemosensitivity by limiting LATS1 activation (part 1 of 2)
Source: EMBO Rep. 2026 Mar 25;27(9):2406–33. doi: 10.1038/s44319-026-00749-w (PMC13172046; doi:10.1038/s44319-026-00749-w)

**Figure 1A**

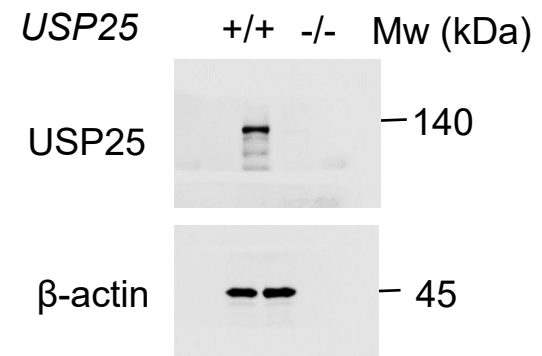

Supplement: Supplementary file 2 — Source data Fig. 1 [file 44319_2026_749_MOESM2_ESM.zip › Figure 1/1A/Western blots 1A.pdf]

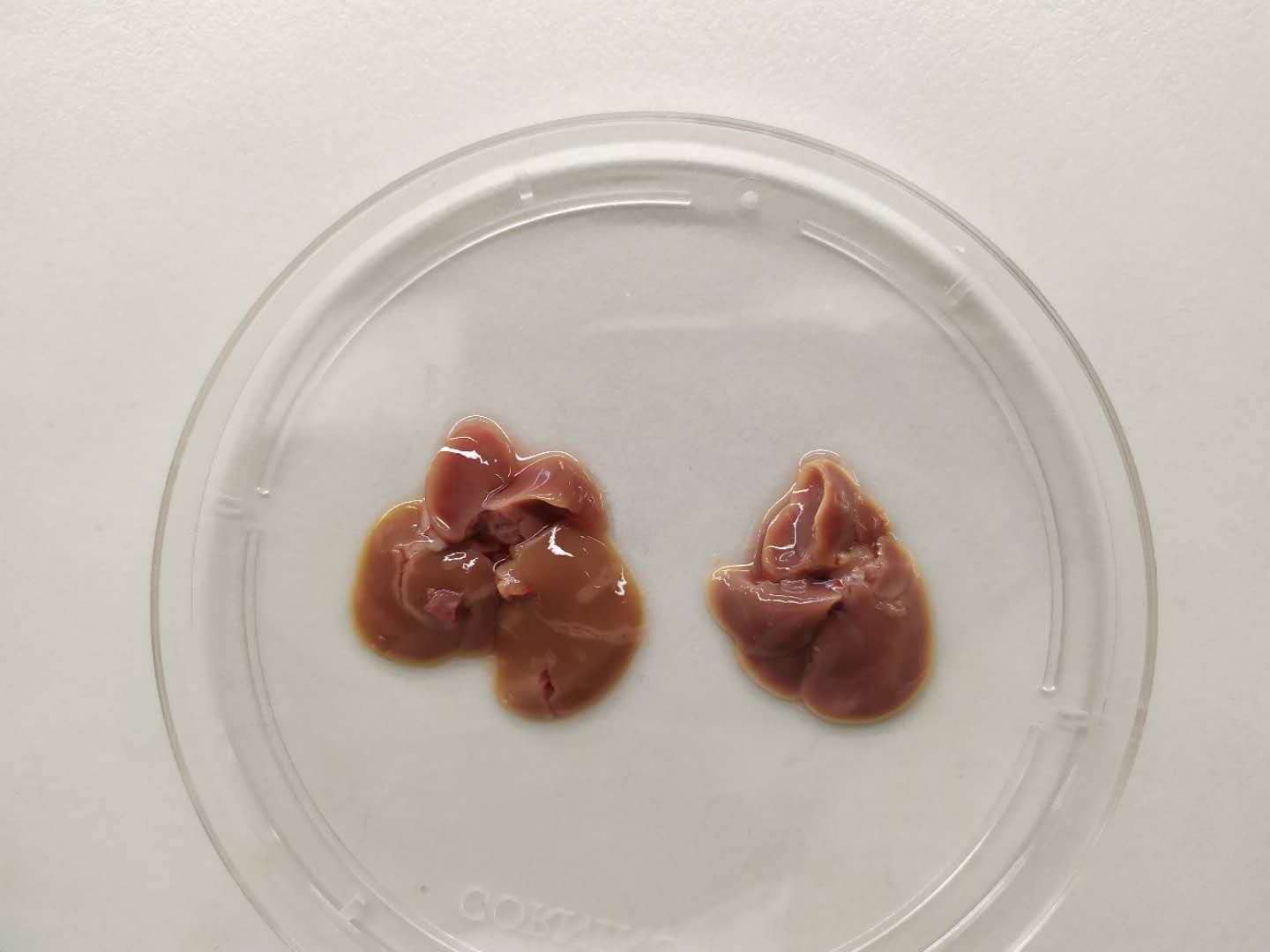

Supplement: Supplementary file 2 — Source data Fig. 1 [file 44319_2026_749_MOESM2_ESM.zip › Figure 1/1B/a 2M USP2WT KO 肝.jpg]

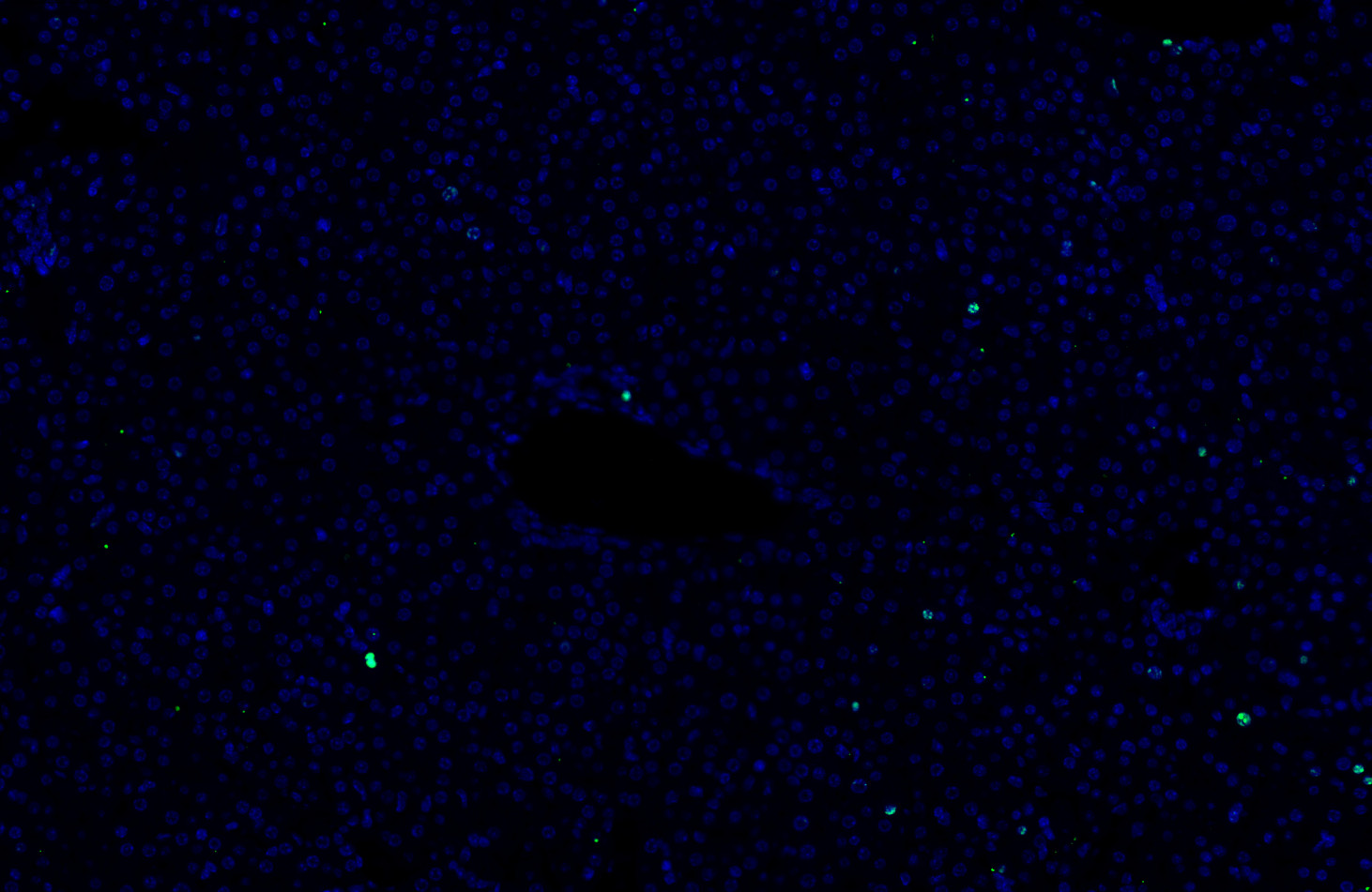

Supplement: Supplementary file 2 — Source data Fig. 1 [file 44319_2026_749_MOESM2_ESM.zip › Figure 1/1E/USP25 KO.jpg]

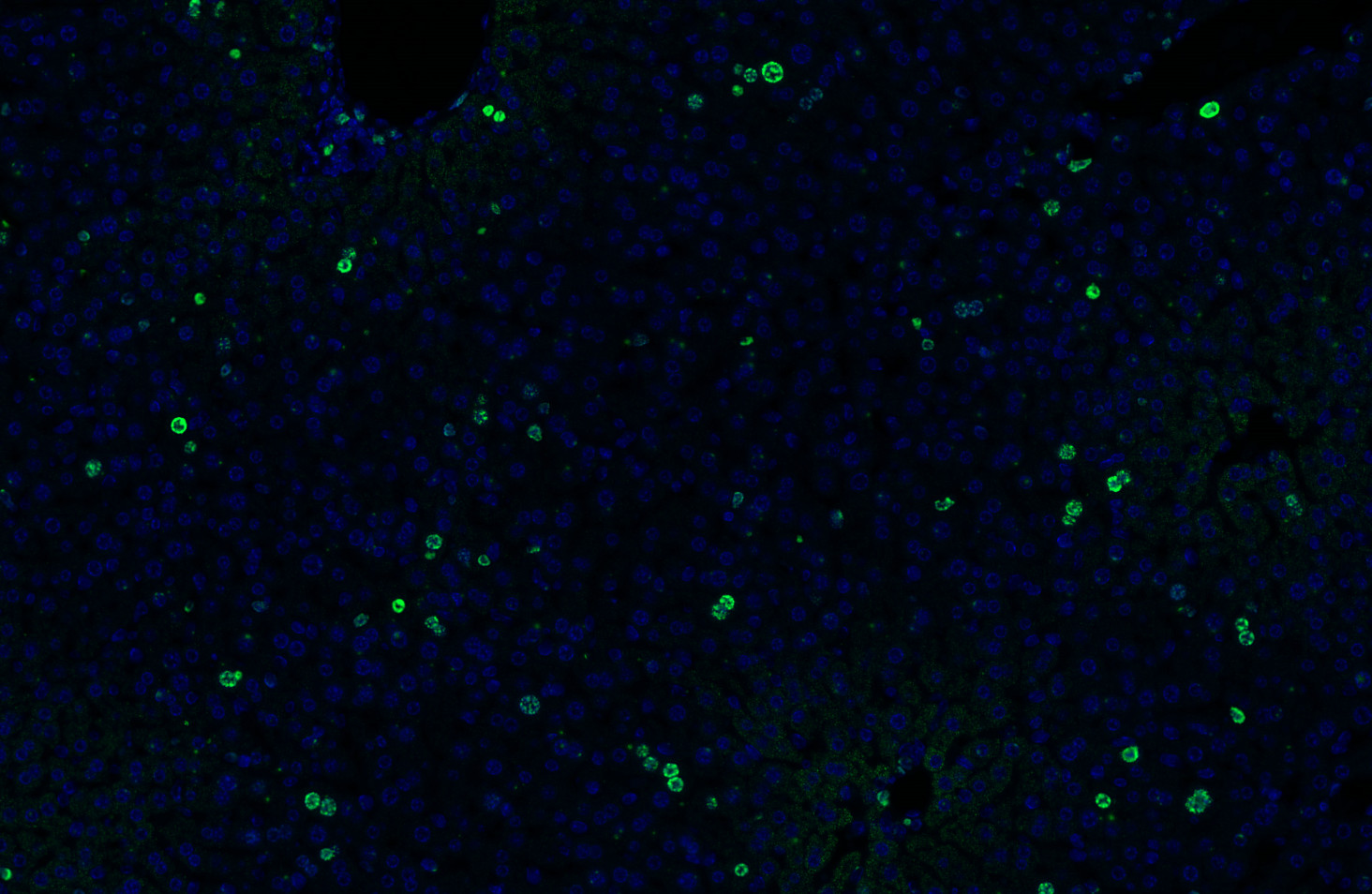

Supplement: Supplementary file 2 — Source data Fig. 1 [file 44319_2026_749_MOESM2_ESM.zip › Figure 1/1E/USP25 WT.jpg]

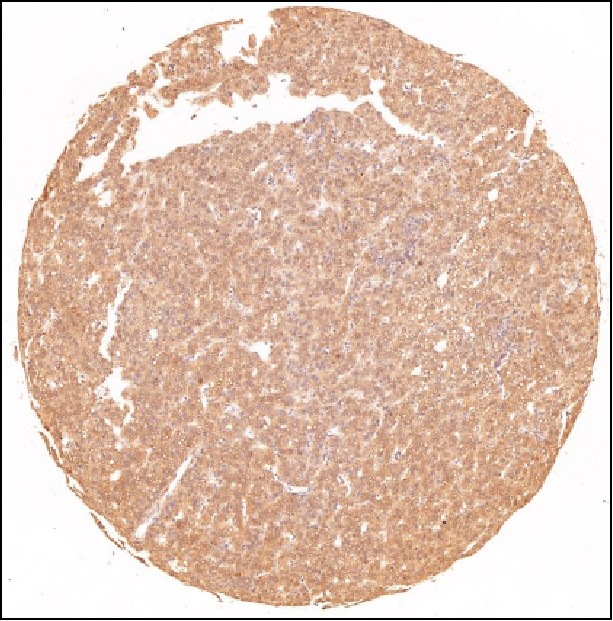

Supplement: Supplementary file 2 — Source data Fig. 1 [file 44319_2026_749_MOESM2_ESM.zip › Figure 1/1I/HCC.jpg]

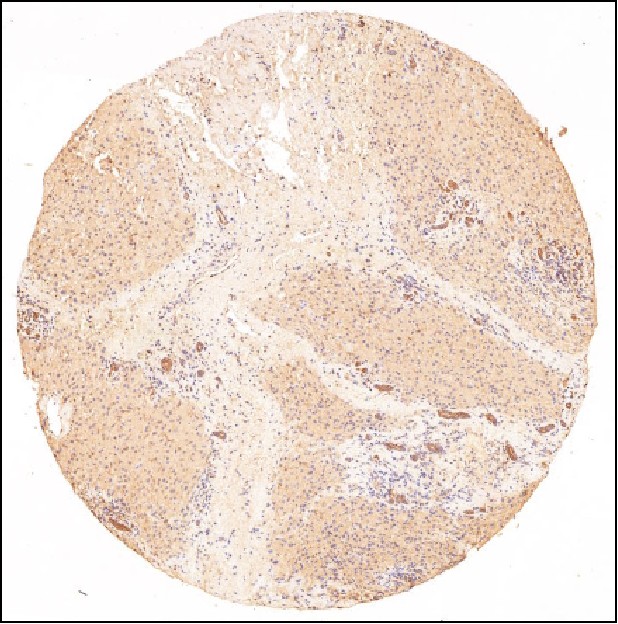

Supplement: Supplementary file 2 — Source data Fig. 1 [file 44319_2026_749_MOESM2_ESM.zip › Figure 1/1I/adjacent tissue.jpg]

**Figure 2A**

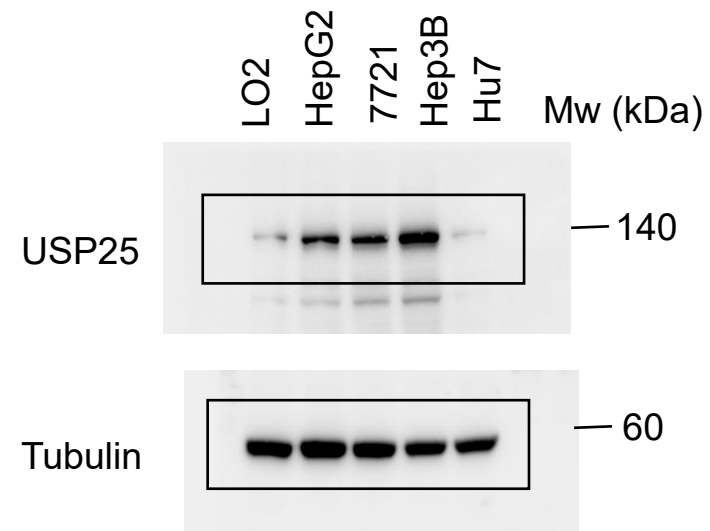

Supplement: Supplementary file 3 — Source data Fig. 2 [file 44319_2026_749_MOESM3_ESM.zip › Figure 2/2A/Western blots 2A.pdf]

**Figure 2B**

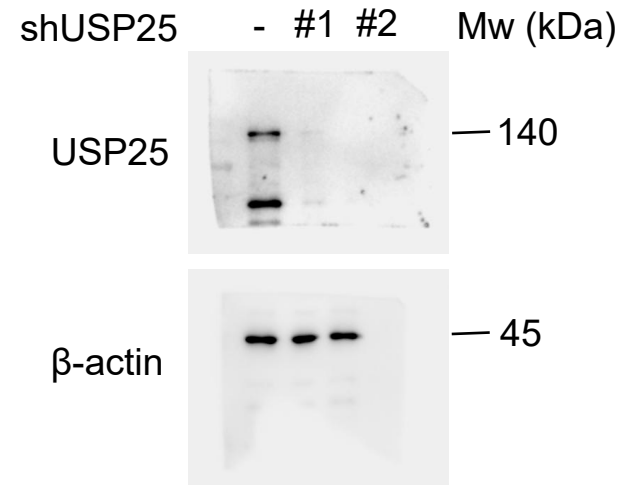

Supplement: Supplementary file 3 — Source data Fig. 2 [file 44319_2026_749_MOESM3_ESM.zip › Figure 2/2B/Western blots 2B.pdf]

**Figure 2C**

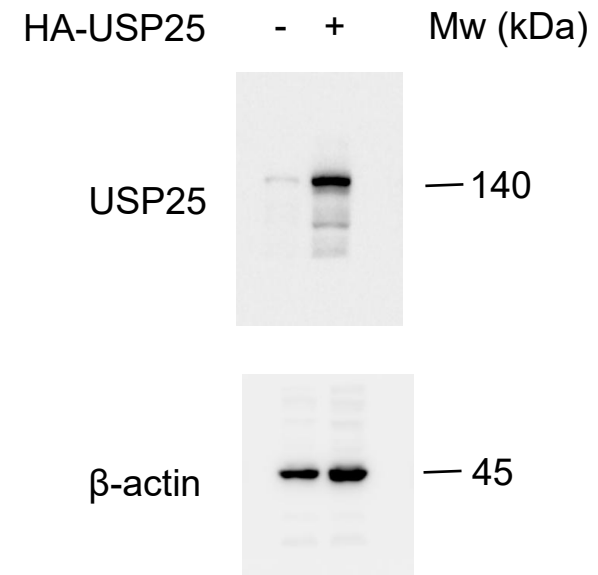

Supplement: Supplementary file 3 — Source data Fig. 2 [file 44319_2026_749_MOESM3_ESM.zip › Figure 2/2C/Western blots 2C.pdf]

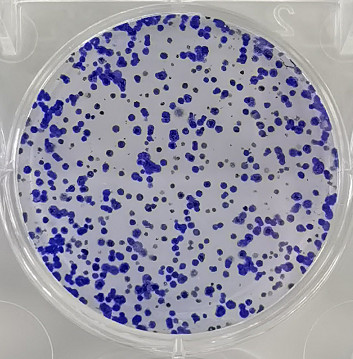

Supplement: Supplementary file 3 — Source data Fig. 2 [file 44319_2026_749_MOESM3_ESM.zip › Figure 2/2F/Ctrl.jpg]

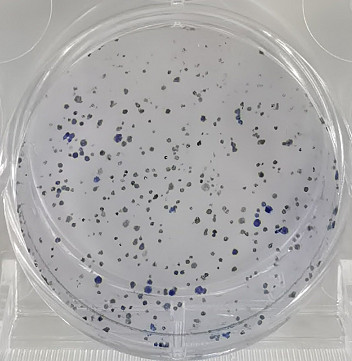

Supplement: Supplementary file 3 — Source data Fig. 2 [file 44319_2026_749_MOESM3_ESM.zip › Figure 2/2F/shUSP25#1.jpg]

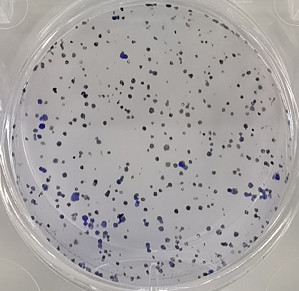

Supplement: Supplementary file 3 — Source data Fig. 2 [file 44319_2026_749_MOESM3_ESM.zip › Figure 2/2F/shUSP25#2.jpg]

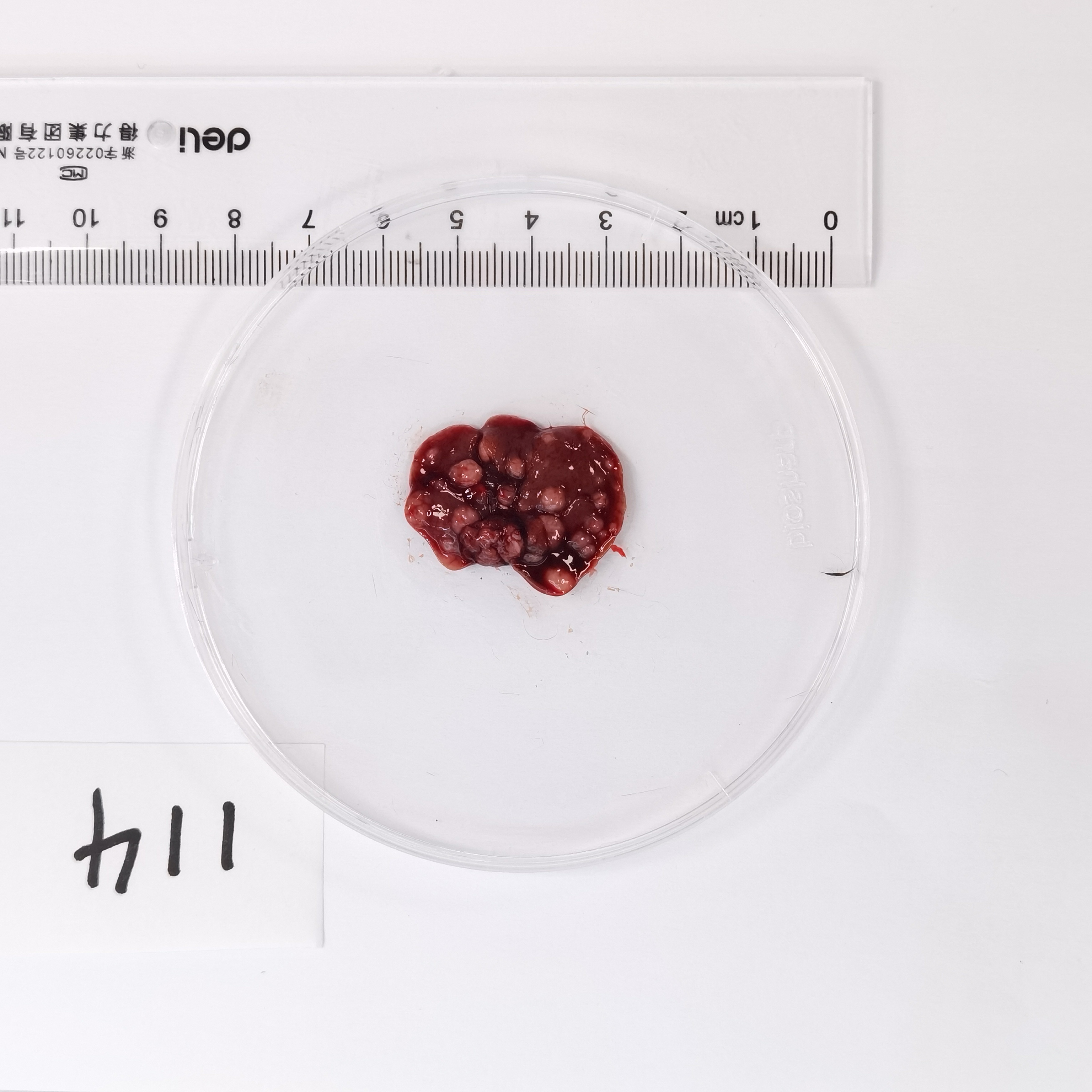

Supplement: Supplementary file 3 — Source data Fig. 2 [file 44319_2026_749_MOESM3_ESM.zip › Figure 2/2I/USP25++.jpg]

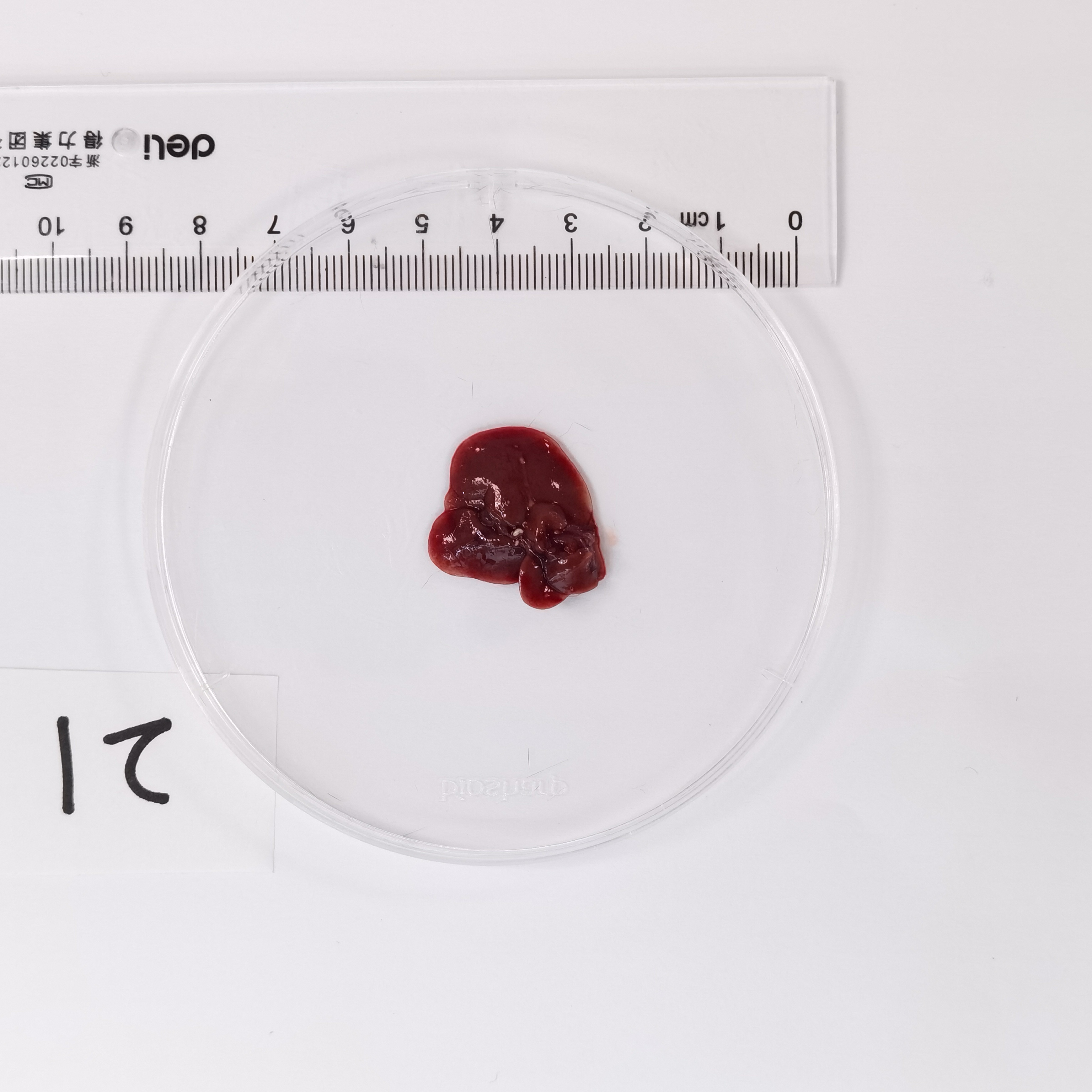

Supplement: Supplementary file 3 — Source data Fig. 2 [file 44319_2026_749_MOESM3_ESM.zip › Figure 2/2I/USP25- -.jpg]

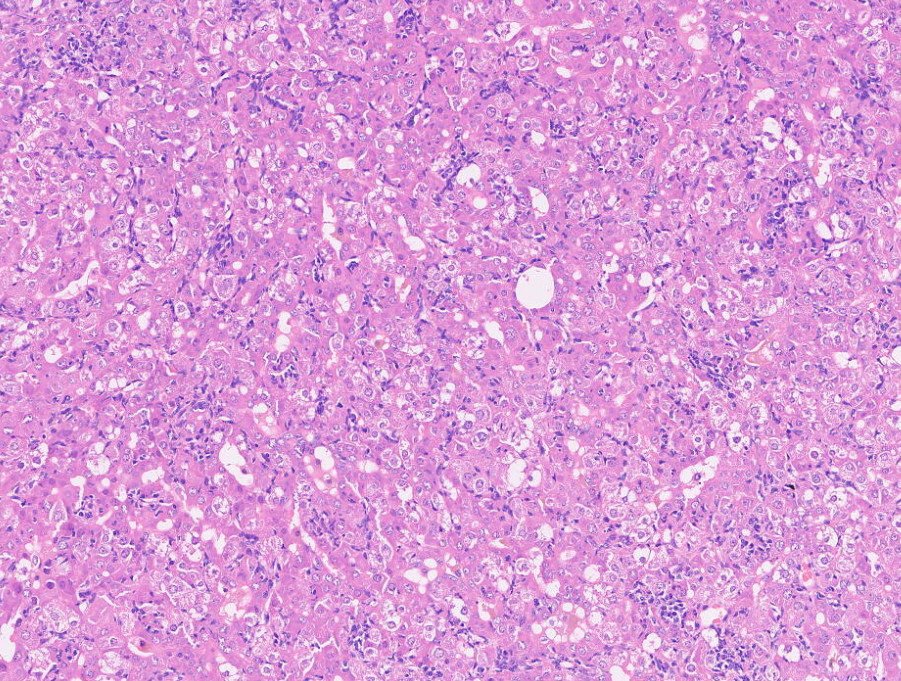

Supplement: Supplementary file 3 — Source data Fig. 2 [file 44319_2026_749_MOESM3_ESM.zip › Figure 2/2M/USP25++.jpg]

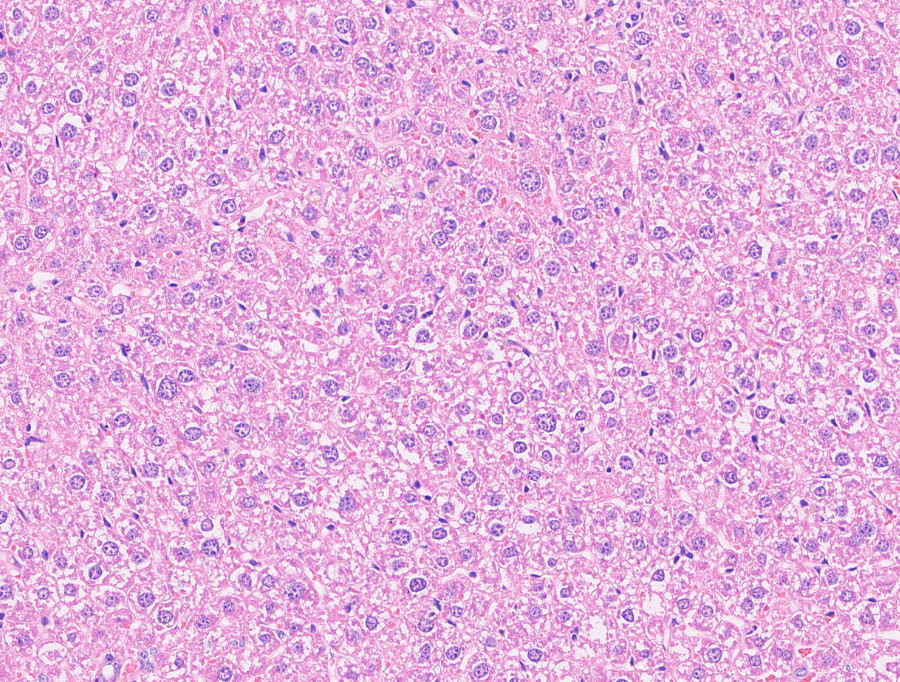

Supplement: Supplementary file 3 — Source data Fig. 2 [file 44319_2026_749_MOESM3_ESM.zip › Figure 2/2M/USP25- -.jpg]

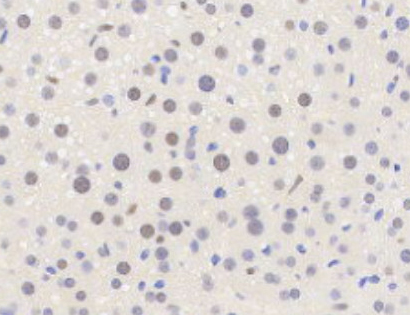

Supplement: Supplementary file 3 — Source data Fig. 2 [file 44319_2026_749_MOESM3_ESM.zip › Figure 2/2N/USP25++-1.jpg]

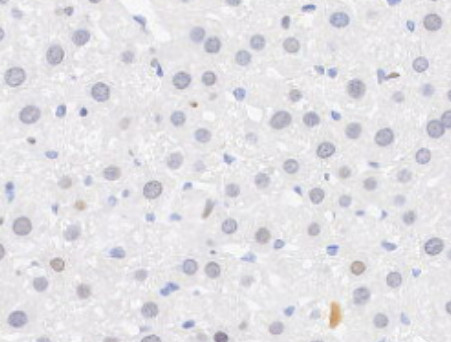

Supplement: Supplementary file 3 — Source data Fig. 2 [file 44319_2026_749_MOESM3_ESM.zip › Figure 2/2N/USP25---1.jpg]

Figure 3C

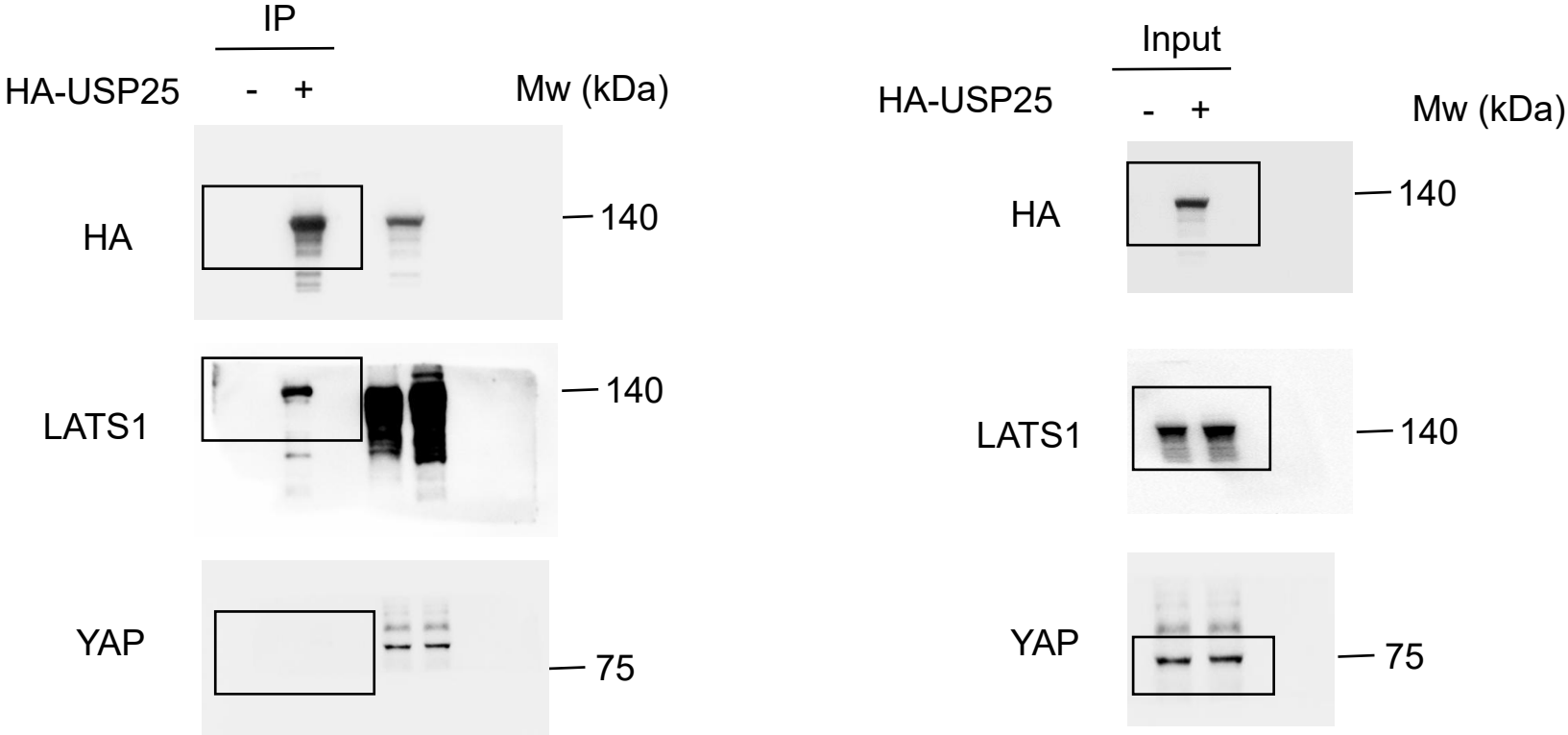

Supplement: Supplementary file 4 — Source data Fig. 3 [file 44319_2026_749_MOESM4_ESM.zip › Figure 3/3C/Western blots 3C.pdf]

Figure 3D

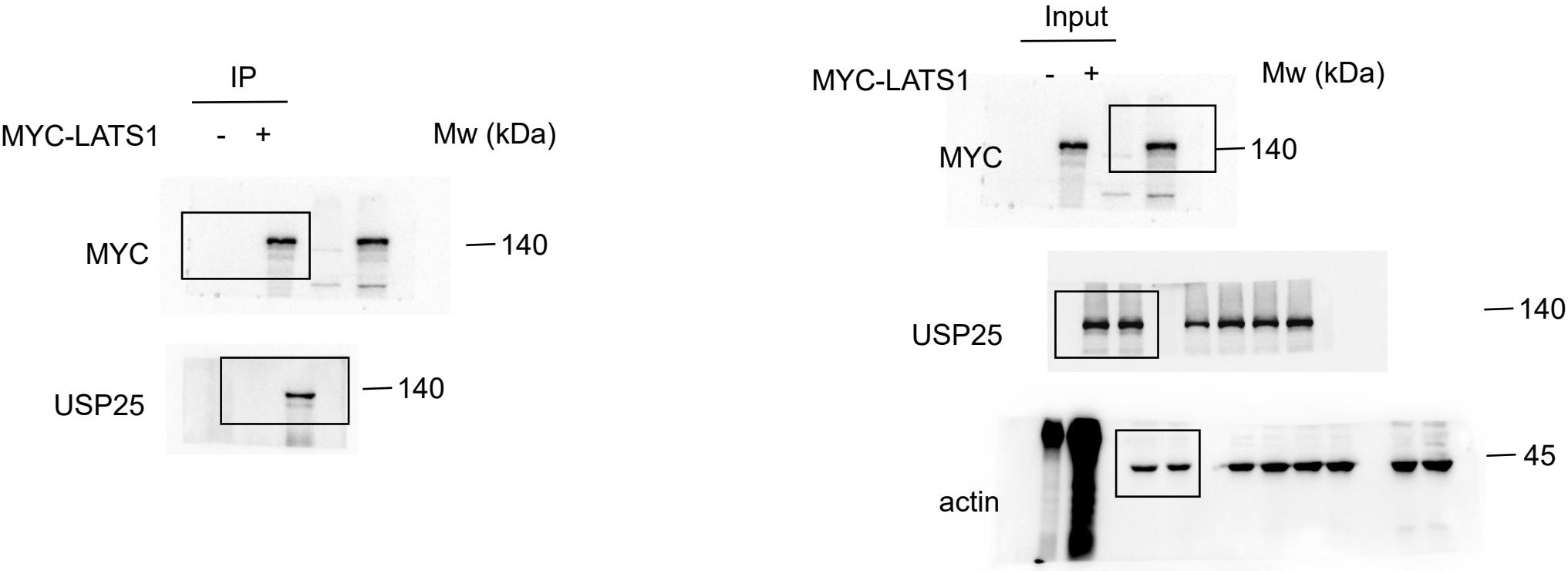

Supplement: Supplementary file 4 — Source data Fig. 3 [file 44319_2026_749_MOESM4_ESM.zip › Figure 3/3D/Western blots 3D.pdf]

Figure 3E

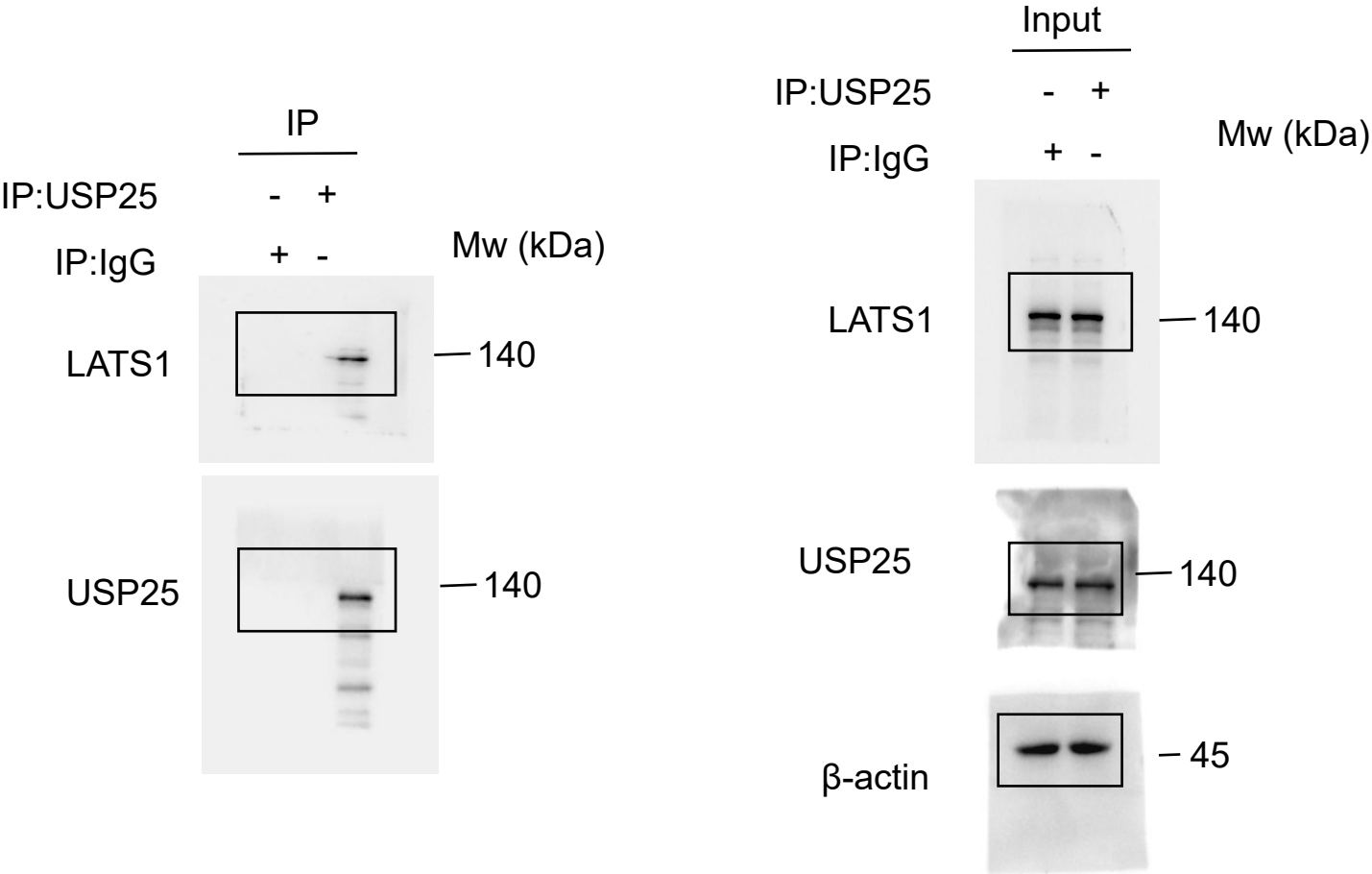

Supplement: Supplementary file 4 — Source data Fig. 3 [file 44319_2026_749_MOESM4_ESM.zip › Figure 3/3E/Western blots 3E.pdf]

Figure 3F

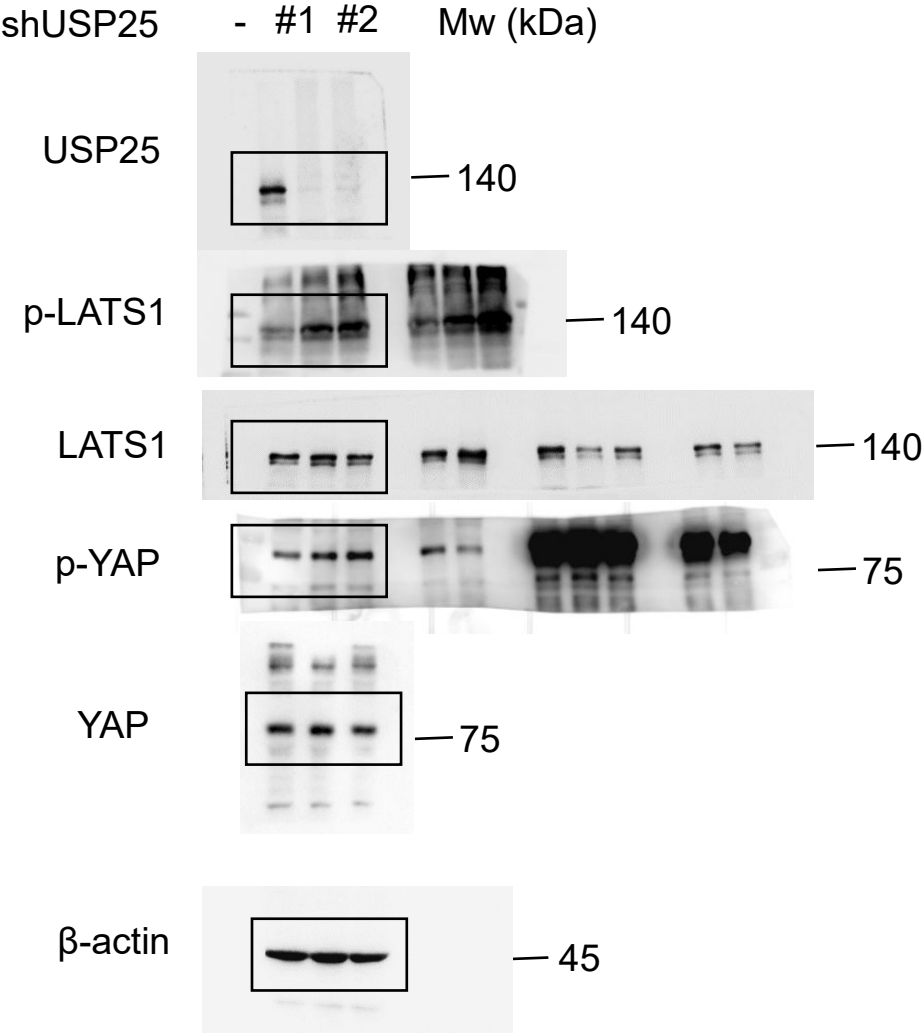

Supplement: Supplementary file 4 — Source data Fig. 3 [file 44319_2026_749_MOESM4_ESM.zip › Figure 3/3F/Western blots 3F.pdf]

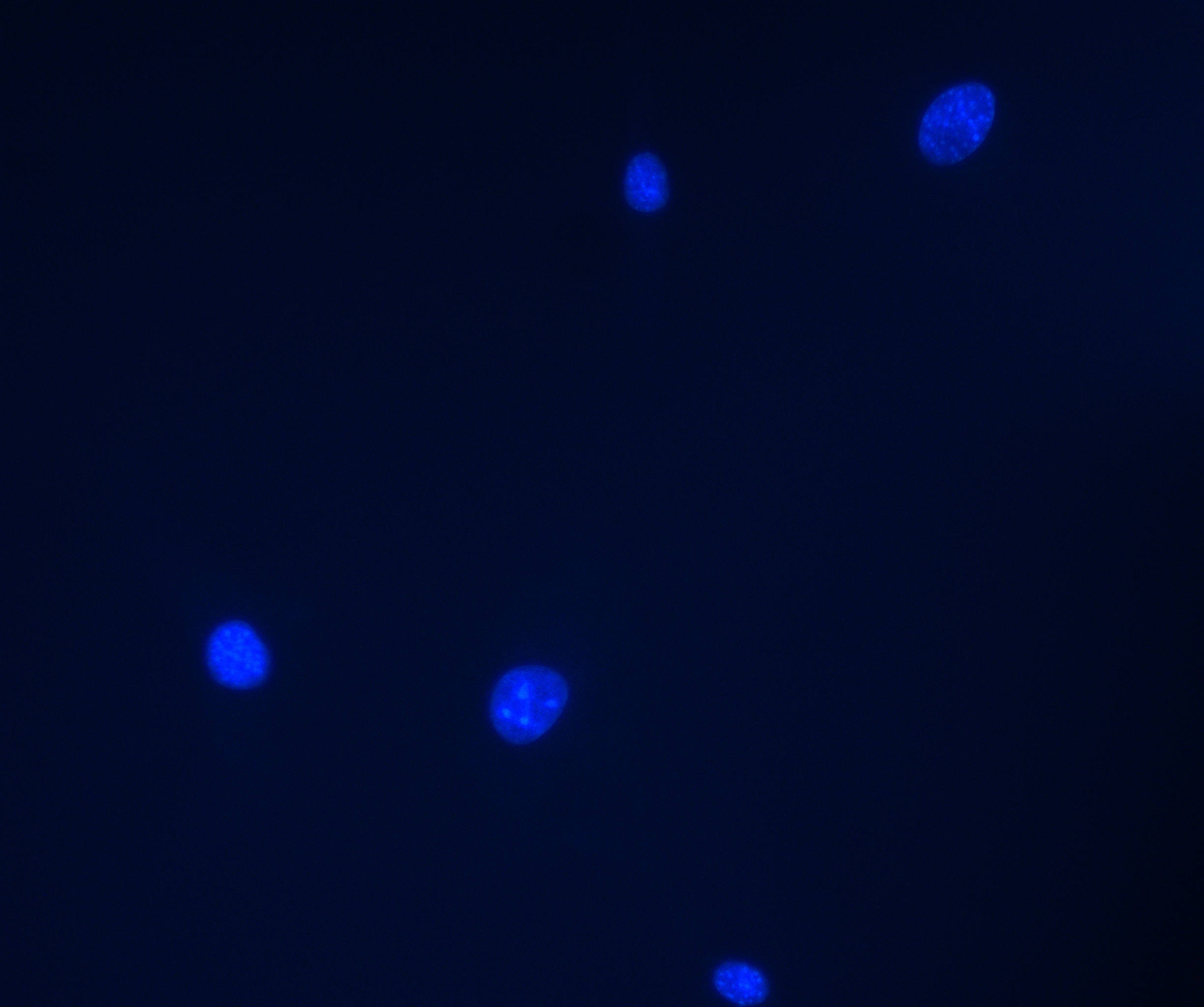

Supplement: Supplementary file 4 — Source data Fig. 3 [file 44319_2026_749_MOESM4_ESM.zip › Figure 3/3G/KO DAPI.jpg]

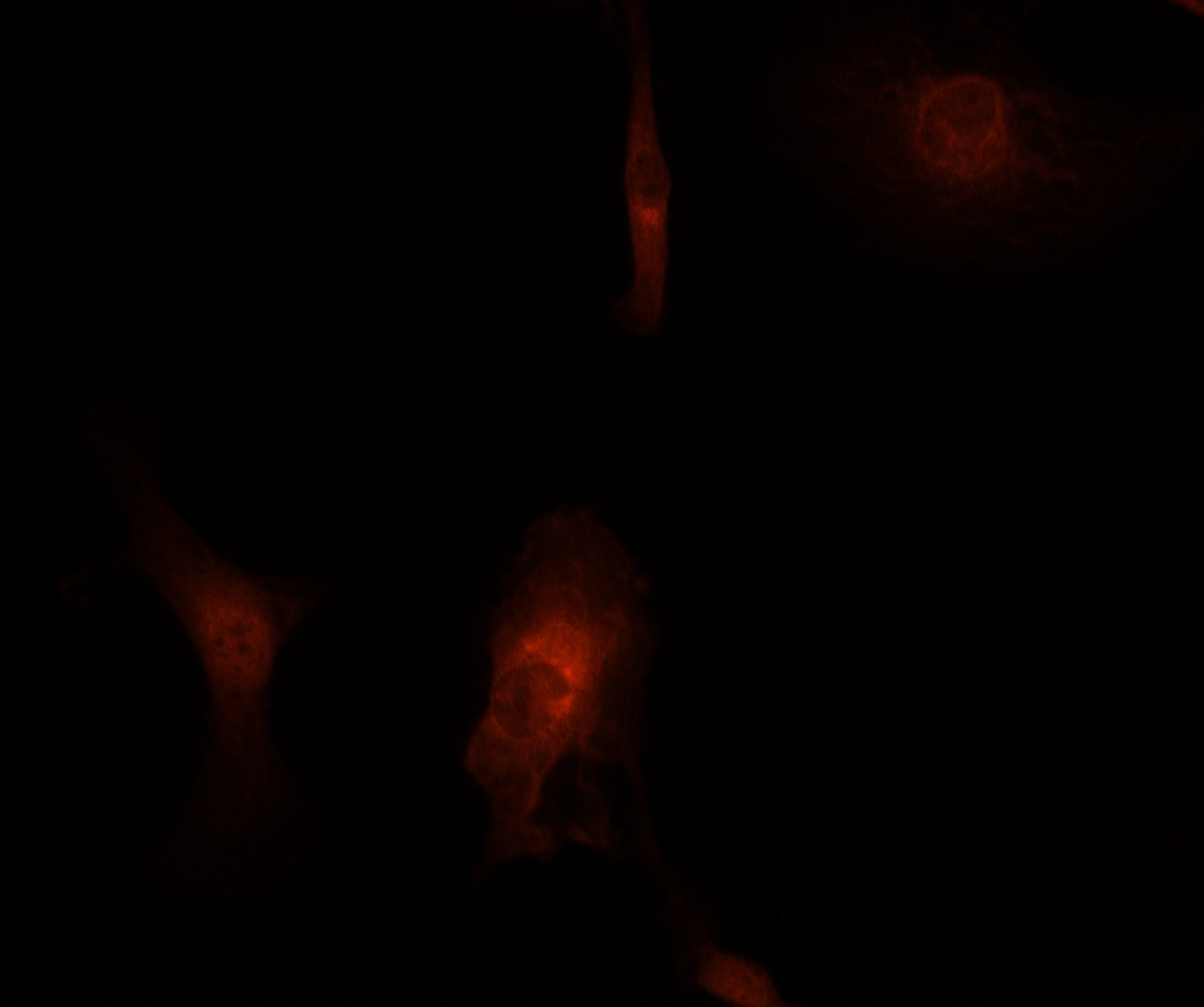

Supplement: Supplementary file 4 — Source data Fig. 3 [file 44319_2026_749_MOESM4_ESM.zip › Figure 3/3G/KO YAP.jpg]

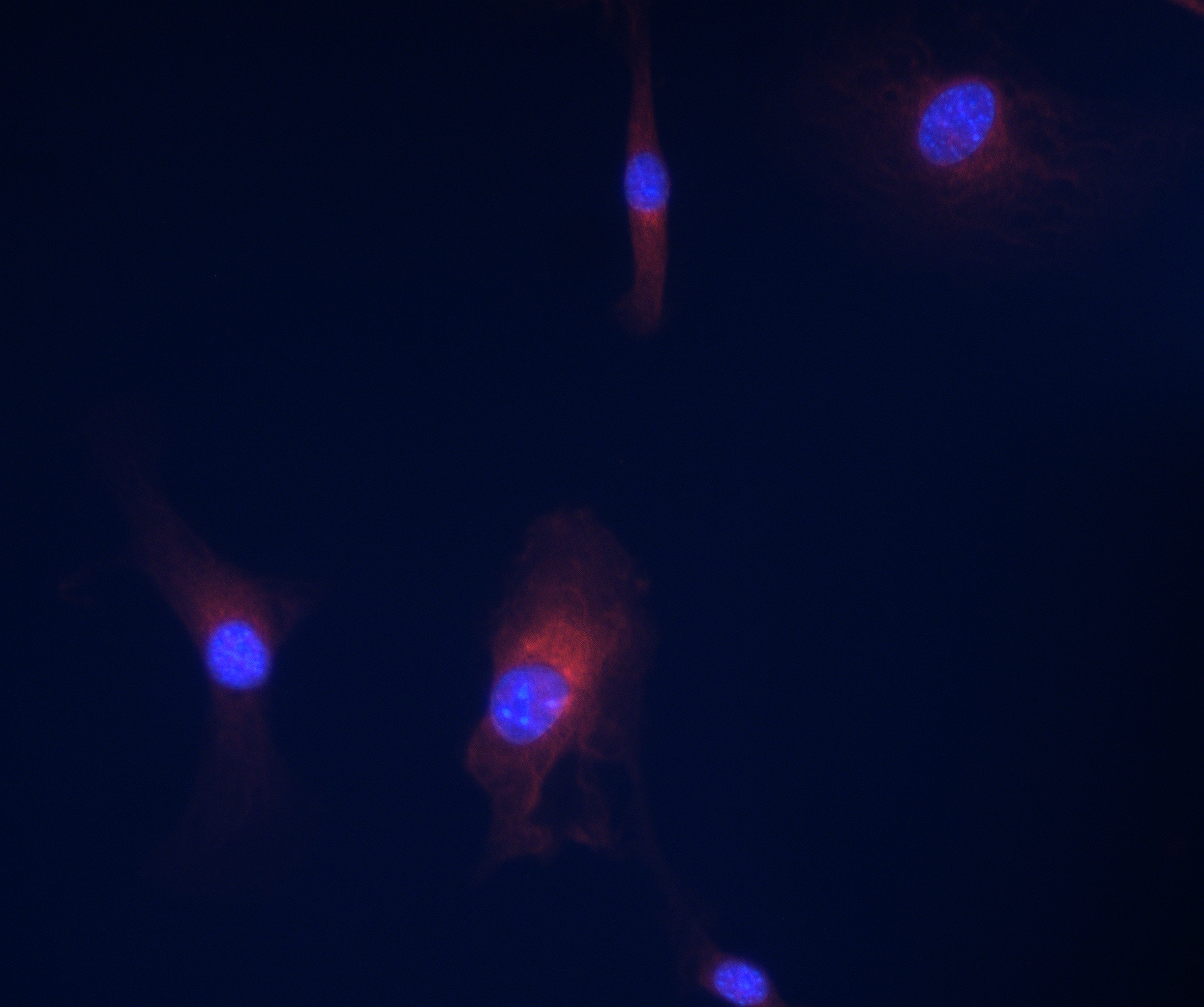

Supplement: Supplementary file 4 — Source data Fig. 3 [file 44319_2026_749_MOESM4_ESM.zip › Figure 3/3G/KO merge.jpg]

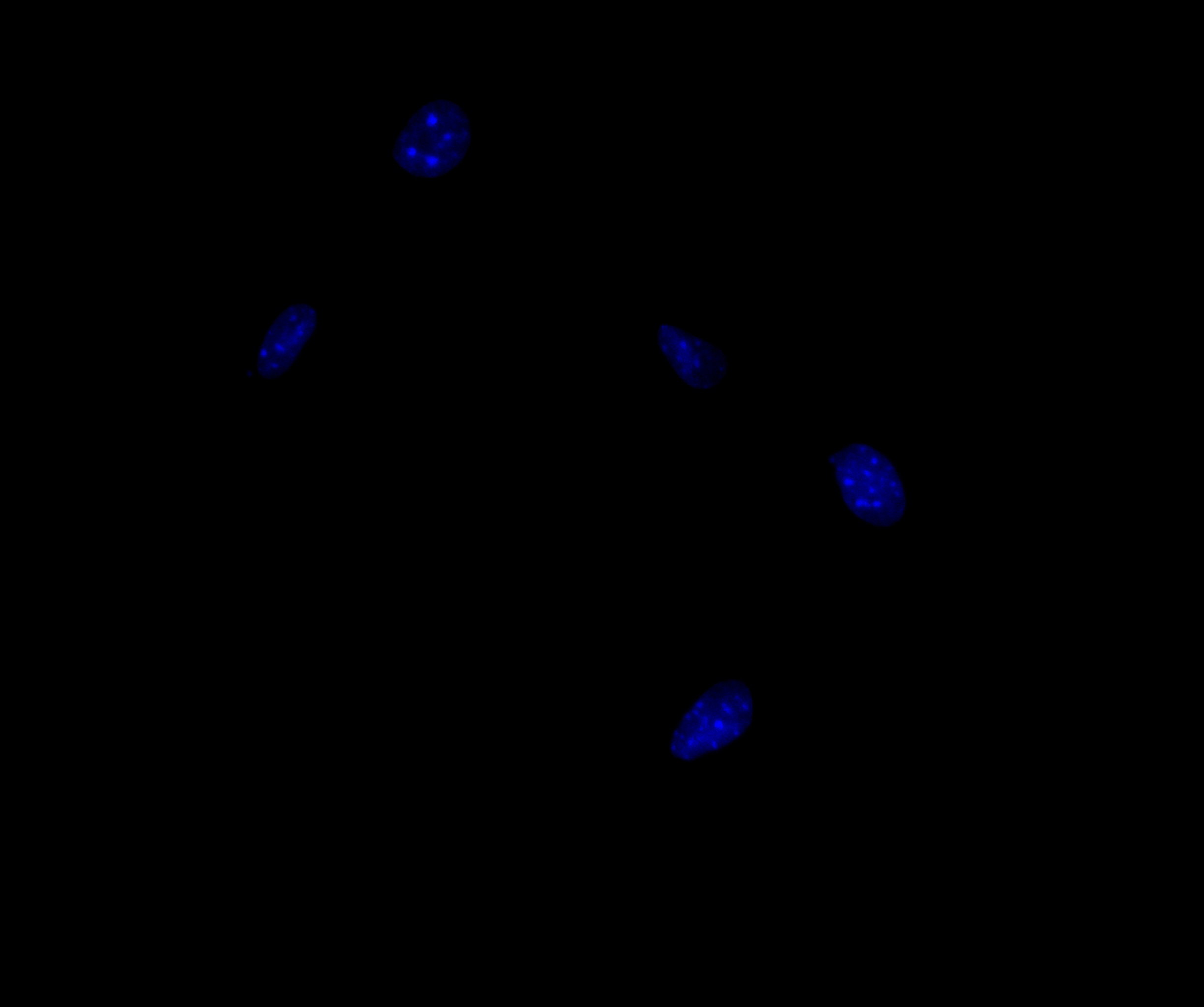

Supplement: Supplementary file 4 — Source data Fig. 3 [file 44319_2026_749_MOESM4_ESM.zip › Figure 3/3G/WT DAPI.jpg]

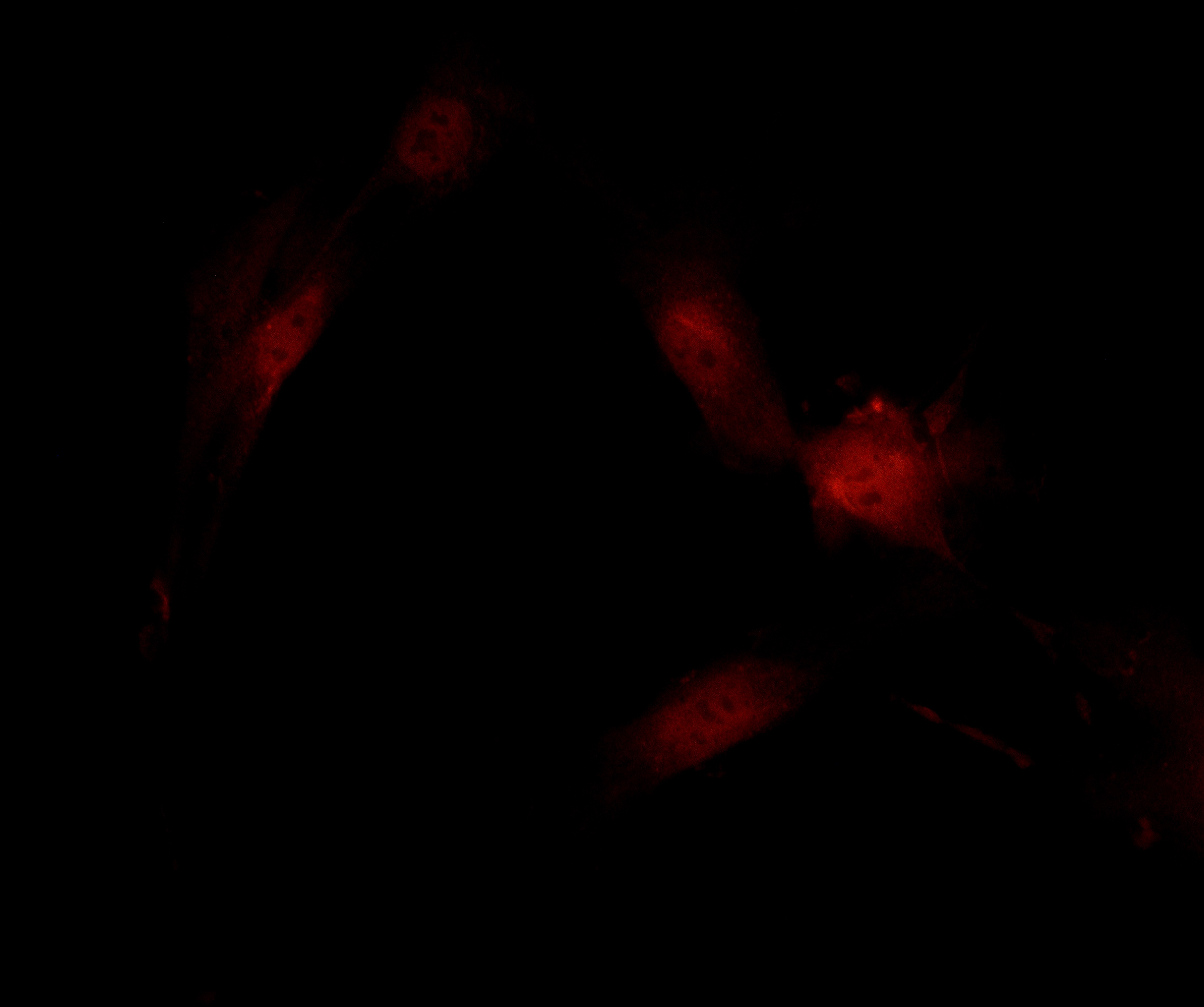

Supplement: Supplementary file 4 — Source data Fig. 3 [file 44319_2026_749_MOESM4_ESM.zip › Figure 3/3G/WT YAP.jpg]

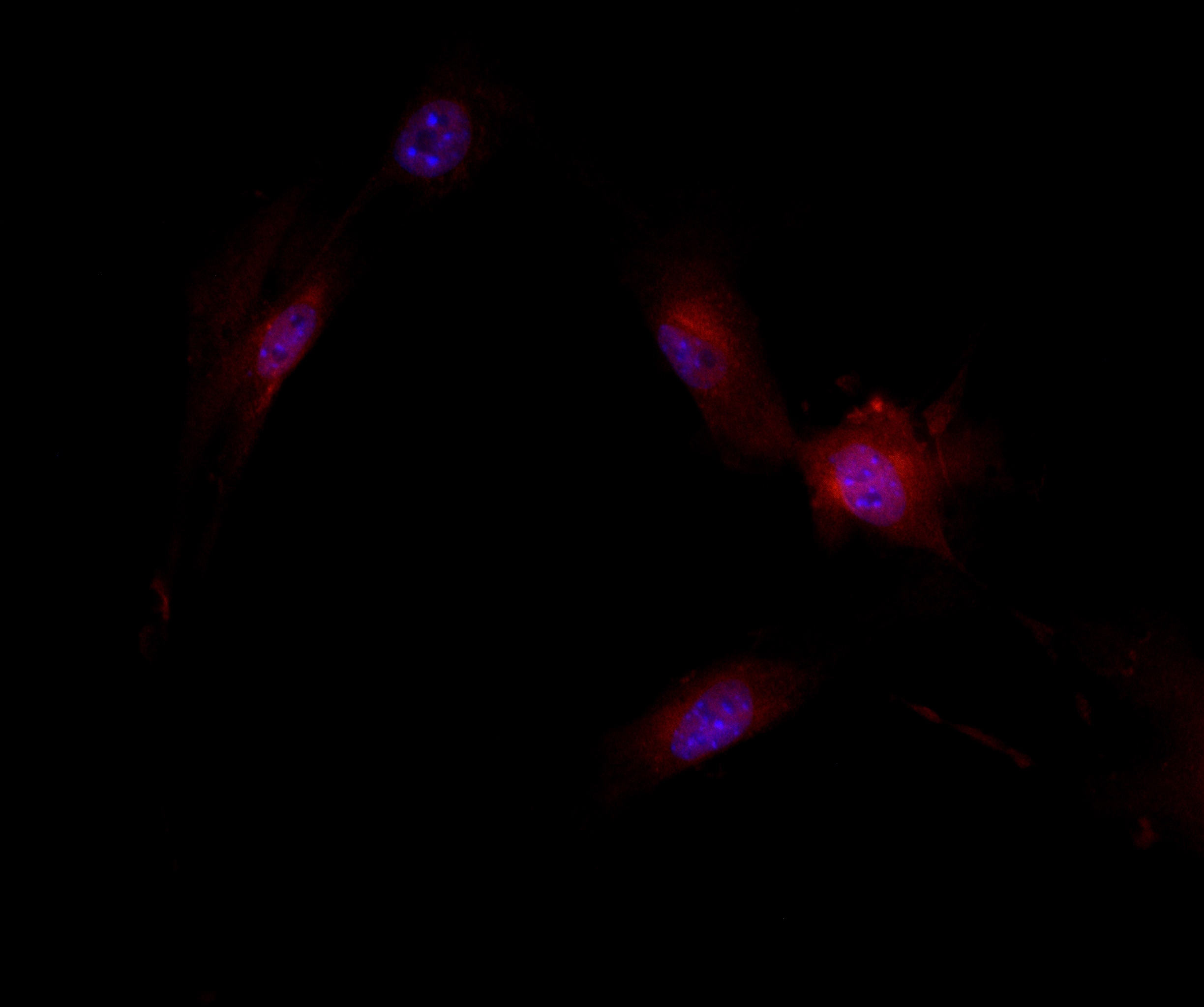

Supplement: Supplementary file 4 — Source data Fig. 3 [file 44319_2026_749_MOESM4_ESM.zip › Figure 3/3G/WT merge.jpg]

Figure 3J

|           |   |   |    |    |
|-----------|---|---|----|----|
| shUSP25   | - | - | #1 | #2 |
| His-ub    | - | + | +  | +  |
| MYC-LATS1 | + | + | +  | +  |

Mw (kDa)

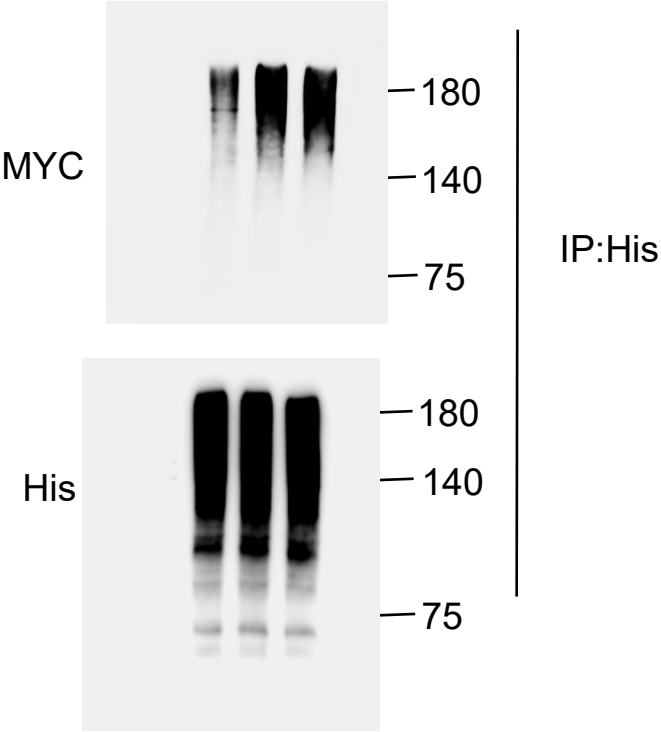

|           |   |   |    |    |
|-----------|---|---|----|----|
| shUSP25   | - | - | #1 | #2 |
| His-ub    | - | + | +  | +  |
| MYC-LATS1 | + | + | +  | +  |

Mw (kDa)

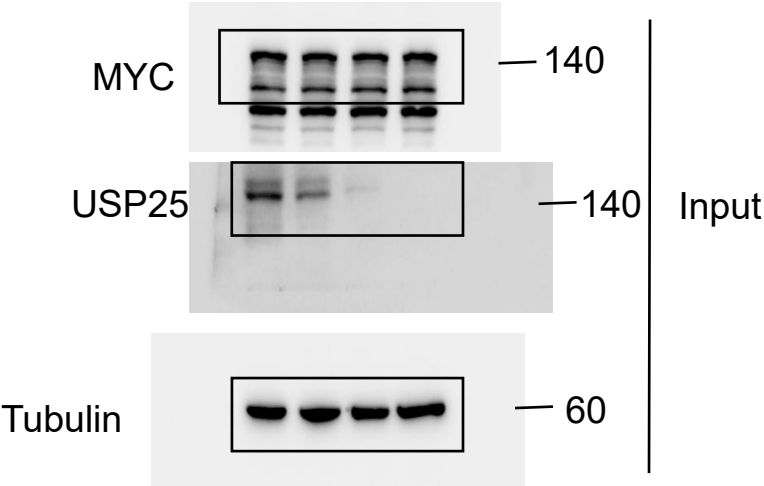

Supplement: Supplementary file 4 — Source data Fig. 3 [file 44319_2026_749_MOESM4_ESM.zip › Figure 3/3J/Western blots 3J.pdf]

Figure 3K

|           |   |    |    |          |
|-----------|---|----|----|----------|
| HA-USP25  | - | WT | CS |          |
| His-ub    | + | +  | +  |          |
| MYC-LATS1 | + | +  | +  | Mw (kDa) |

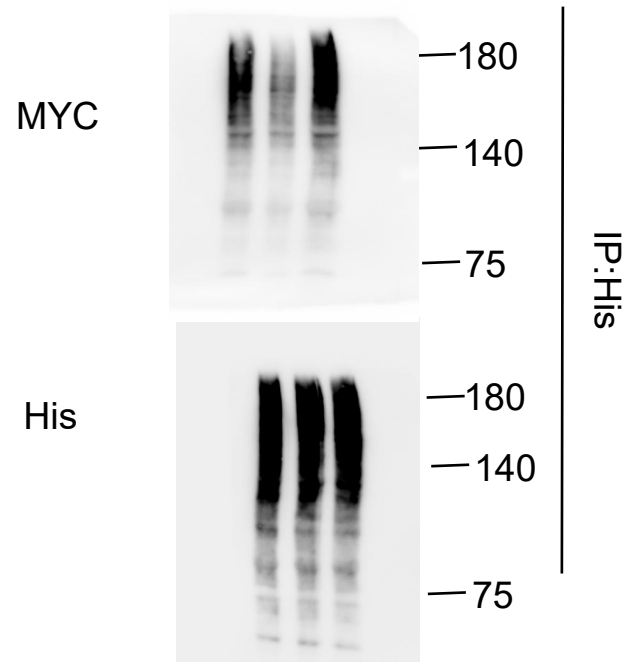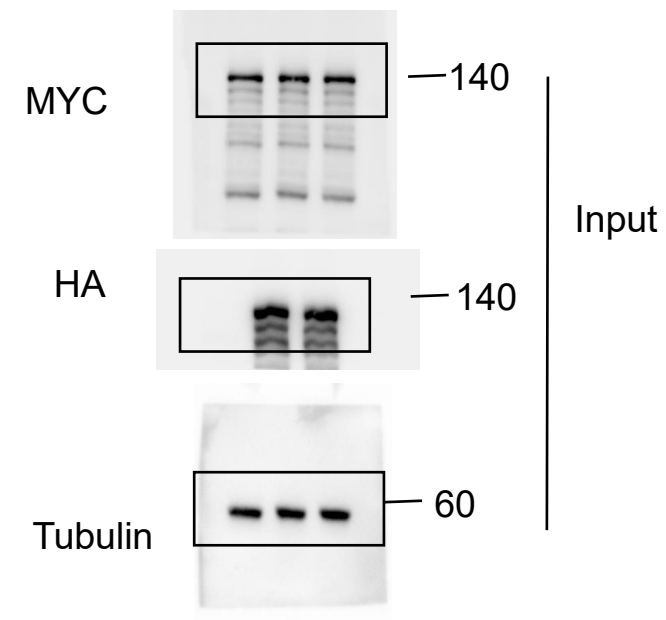

Supplement: Supplementary file 4 — Source data Fig. 3 [file 44319_2026_749_MOESM4_ESM.zip › Figure 3/3K/Western blots 3K.pdf]

Figure 3L

| His-ub    | WT |   | K48 |   | K63 |   | Mw (kDa) |
|-----------|----|---|-----|---|-----|---|----------|
| shUSP25   | -  | + | -   | + | -   | + |          |
| MYC-LATS1 | +  | + | +   | + | +   | + |          |

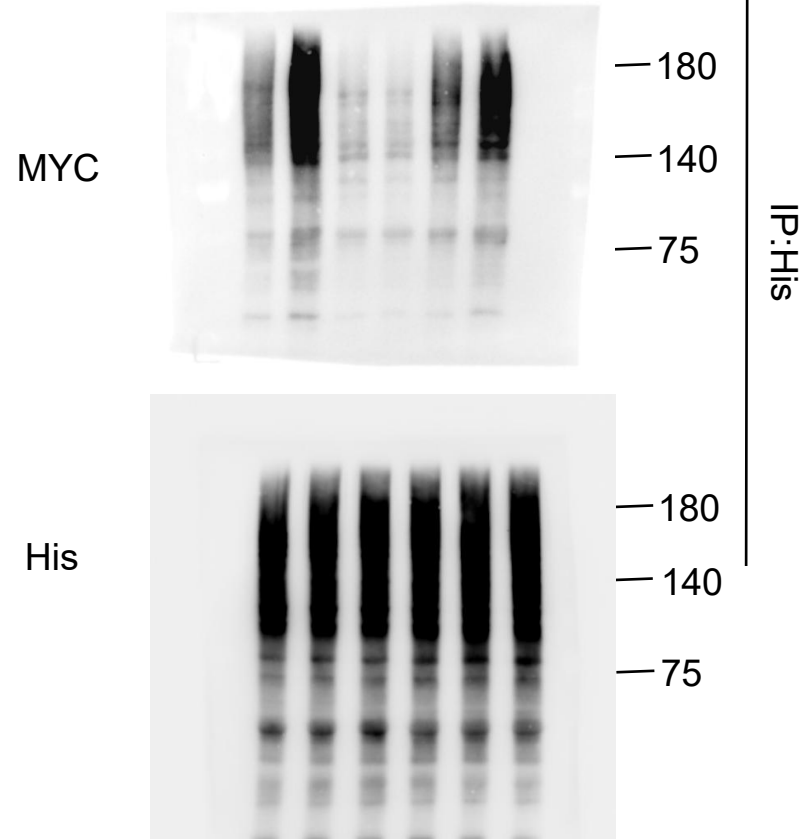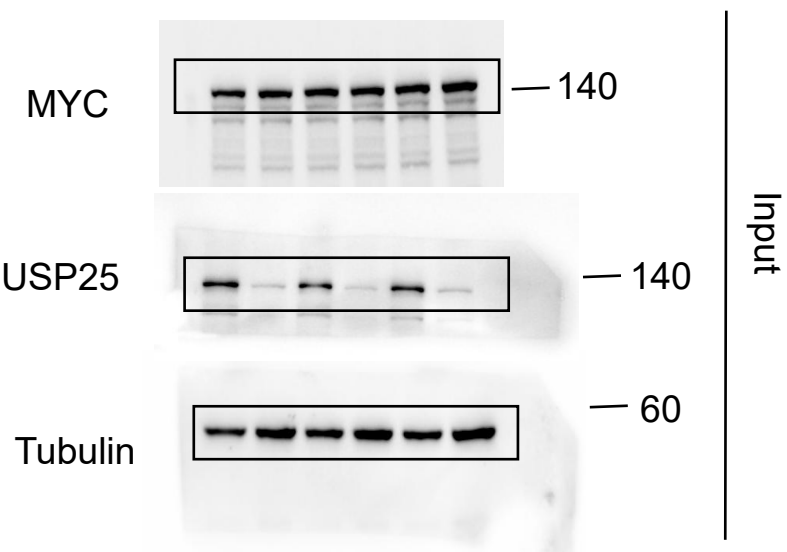

Supplement: Supplementary file 4 — Source data Fig. 3 [file 44319_2026_749_MOESM4_ESM.zip › Figure 3/3L/Western blots 3L.pdf]

Figure 3M

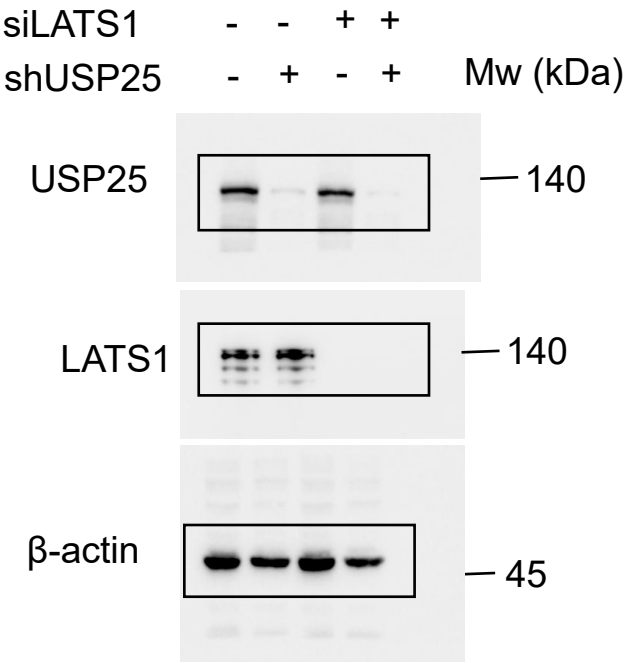

Supplement: Supplementary file 4 — Source data Fig. 3 [file 44319_2026_749_MOESM4_ESM.zip › Figure 3/3M/Western blots 3M.pdf]

Figure 4B

|           |    |   |       |   |       |   |        |   |          |
|-----------|----|---|-------|---|-------|---|--------|---|----------|
| Myc-LATS1 | WT |   | K688R |   | K751R |   | K1005R |   |          |
| shUSP25   | -  | + | -     | + | -     | + | -      | + |          |
| His-ub    | +  | + | +     | + | +     | + | +      | + | Mw (kDa) |

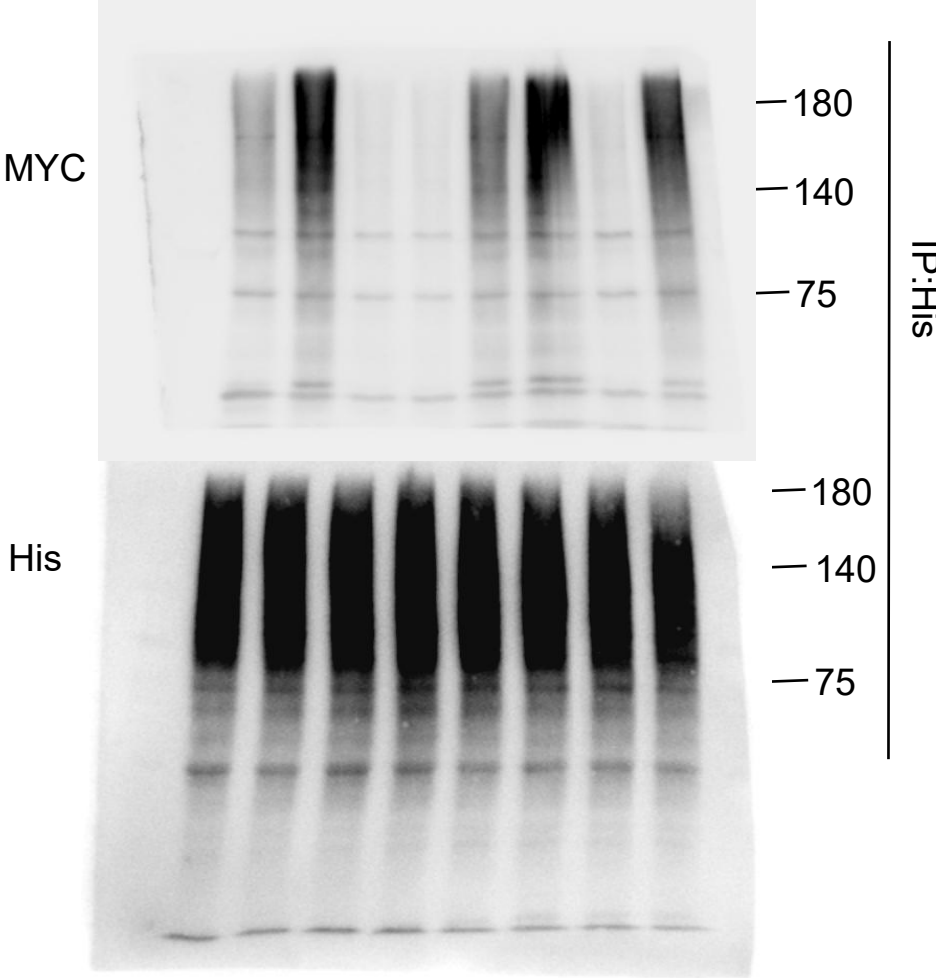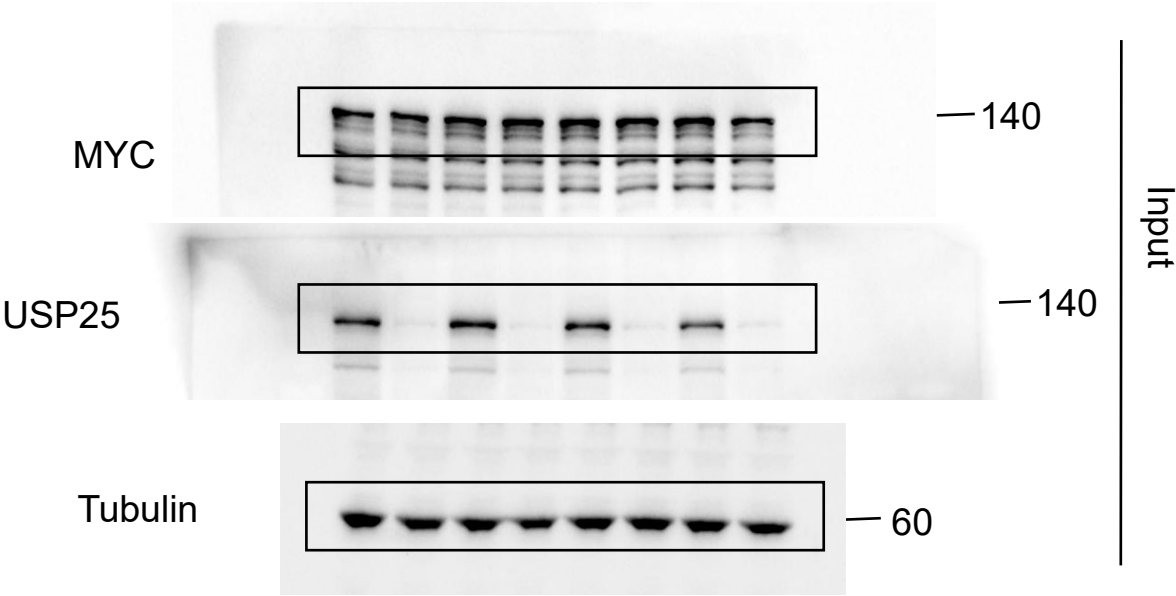

Supplement: Supplementary file 5 — Source data Fig. 4 [file 44319_2026_749_MOESM5_ESM.zip › Figure 4/4B/Western blots 4B.pdf]

Figure 4D

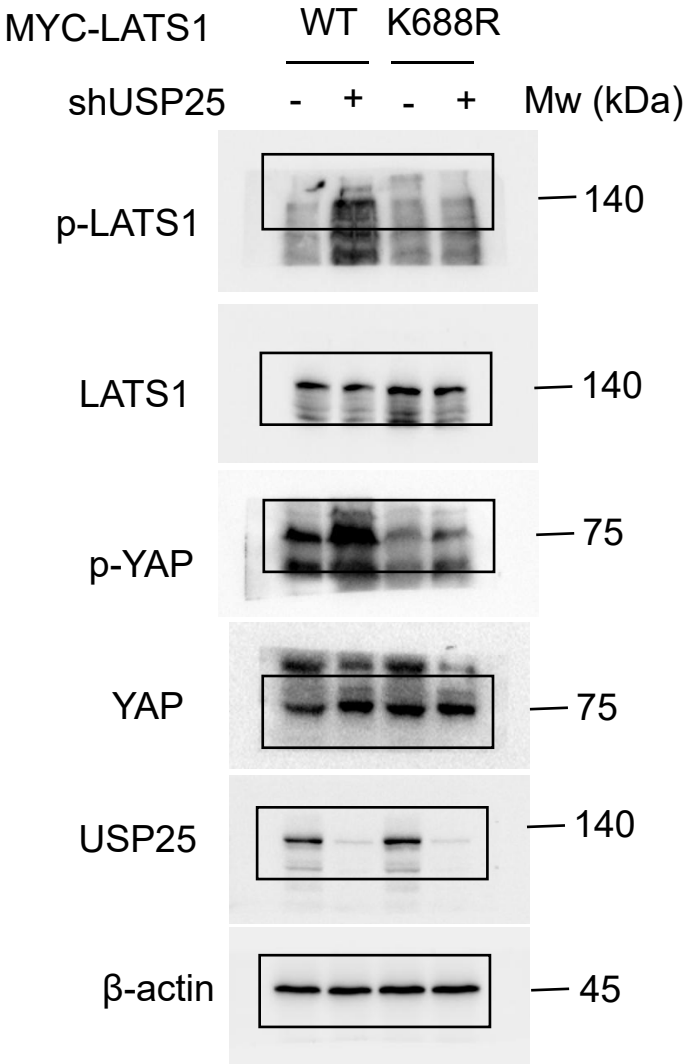

Supplement: Supplementary file 5 — Source data Fig. 4 [file 44319_2026_749_MOESM5_ESM.zip › Figure 4/4D/Western blots 4D.pdf]

Figure 4F

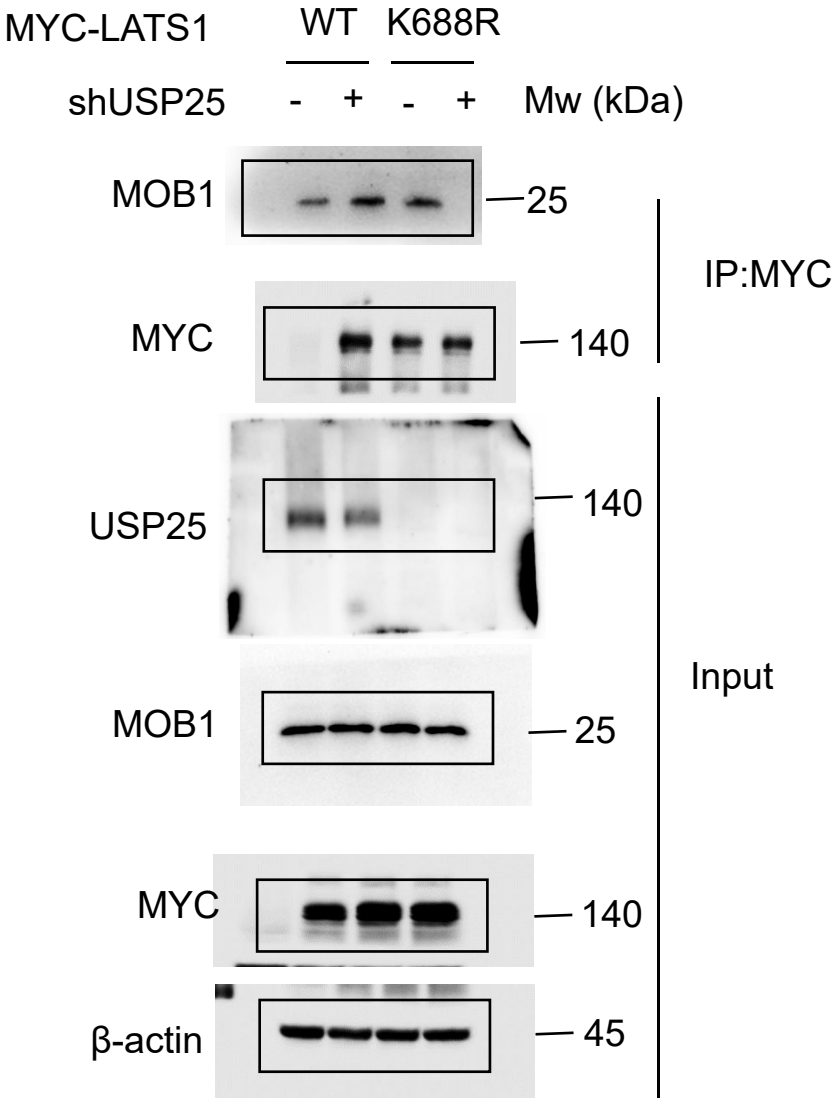

Supplement: Supplementary file 5 — Source data Fig. 4 [file 44319_2026_749_MOESM5_ESM.zip › Figure 4/4F/Western blots 4F.pdf]

Figure 4G

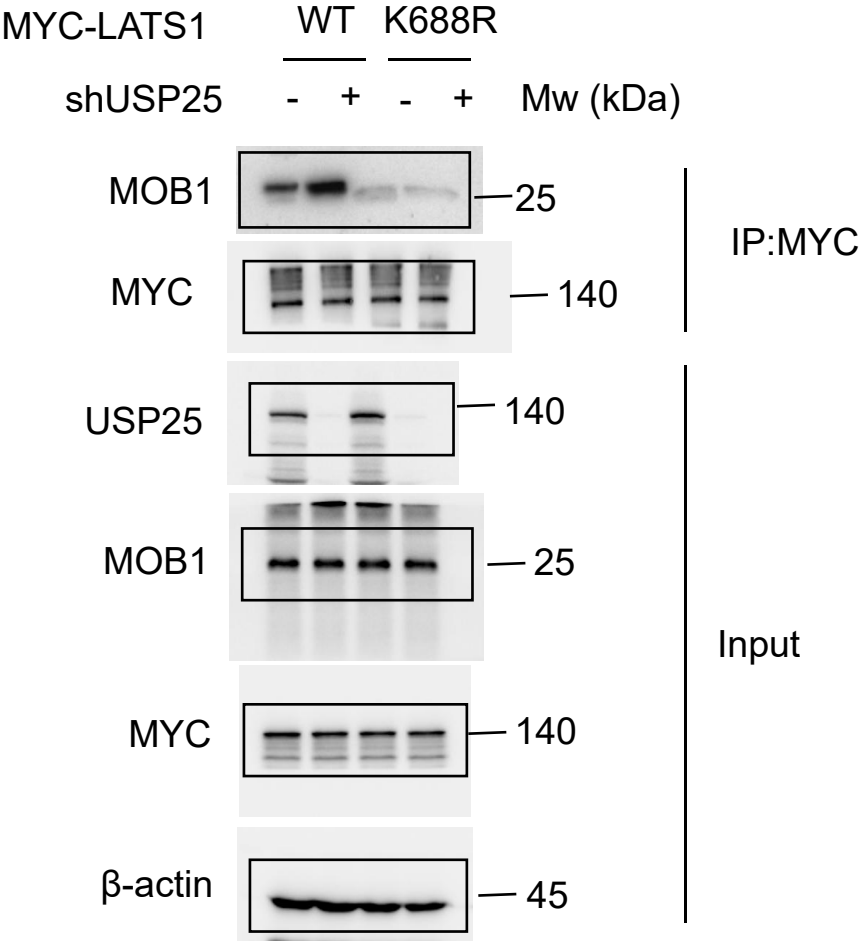

Supplement: Supplementary file 5 — Source data Fig. 4 [file 44319_2026_749_MOESM5_ESM.zip › Figure 4/4G/Western blots 4G.pdf]

Figure 4H

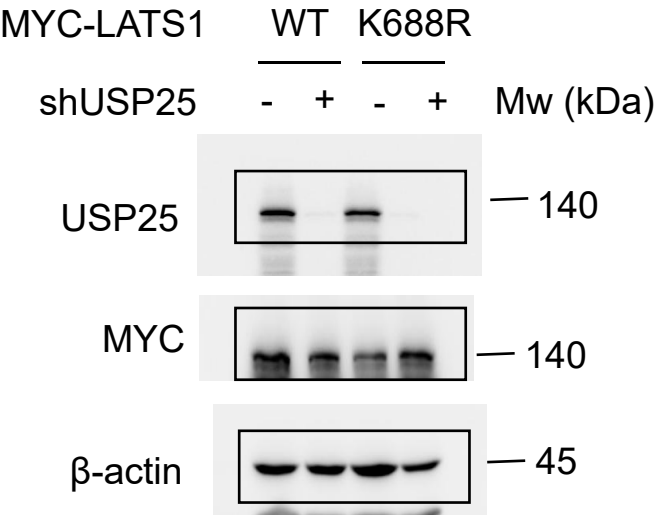

Supplement: Supplementary file 5 — Source data Fig. 4 [file 44319_2026_749_MOESM5_ESM.zip › Figure 4/4H/Western blots 4H.pdf]

Figure 5A

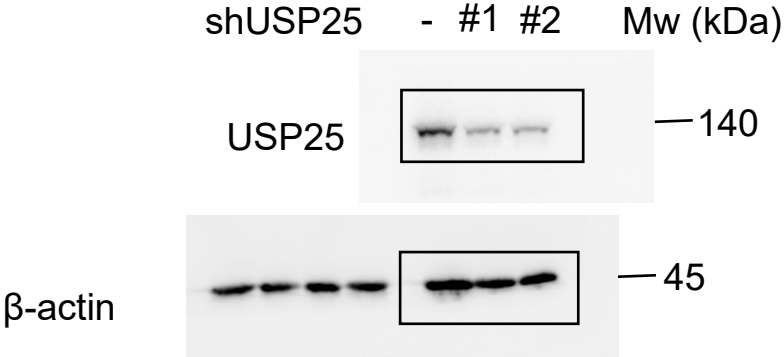

Supplement: Supplementary file 6 — Source data Fig. 5 [file 44319_2026_749_MOESM6_ESM.zip › Figure 5/5A/Western blots 5A.pdf]

Figure 5D

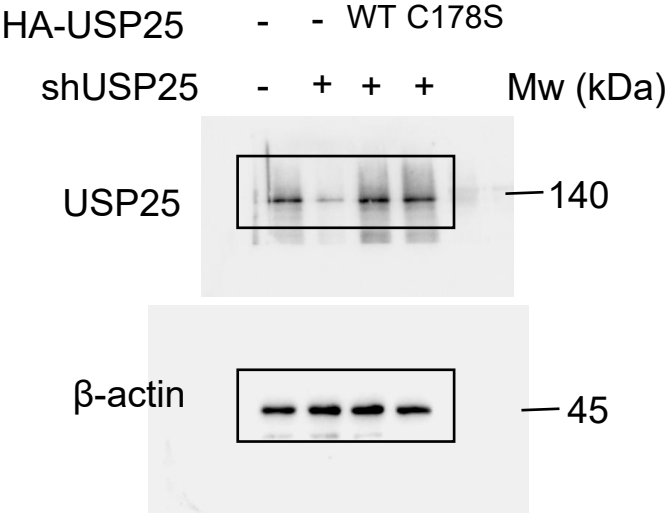

Supplement: Supplementary file 6 — Source data Fig. 5 [file 44319_2026_749_MOESM6_ESM.zip › Figure 5/5D/Western blots 5D.pdf]

Figure 5F

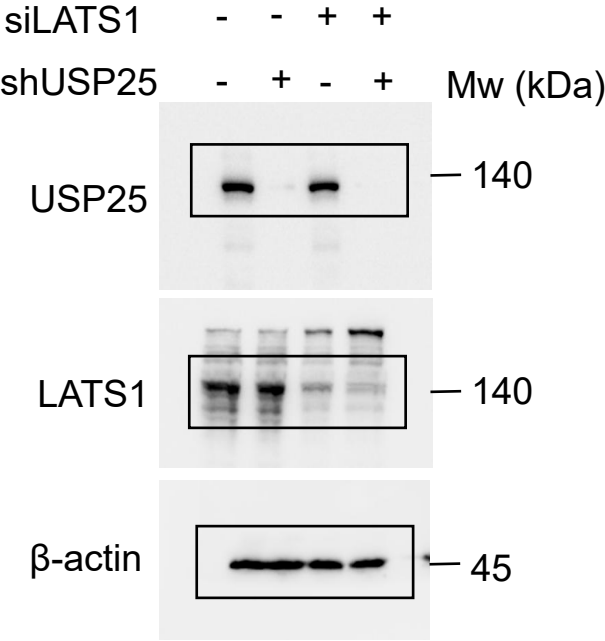

Supplement: Supplementary file 6 — Source data Fig. 5 [file 44319_2026_749_MOESM6_ESM.zip › Figure 5/5F/Western blots 5F.pdf]

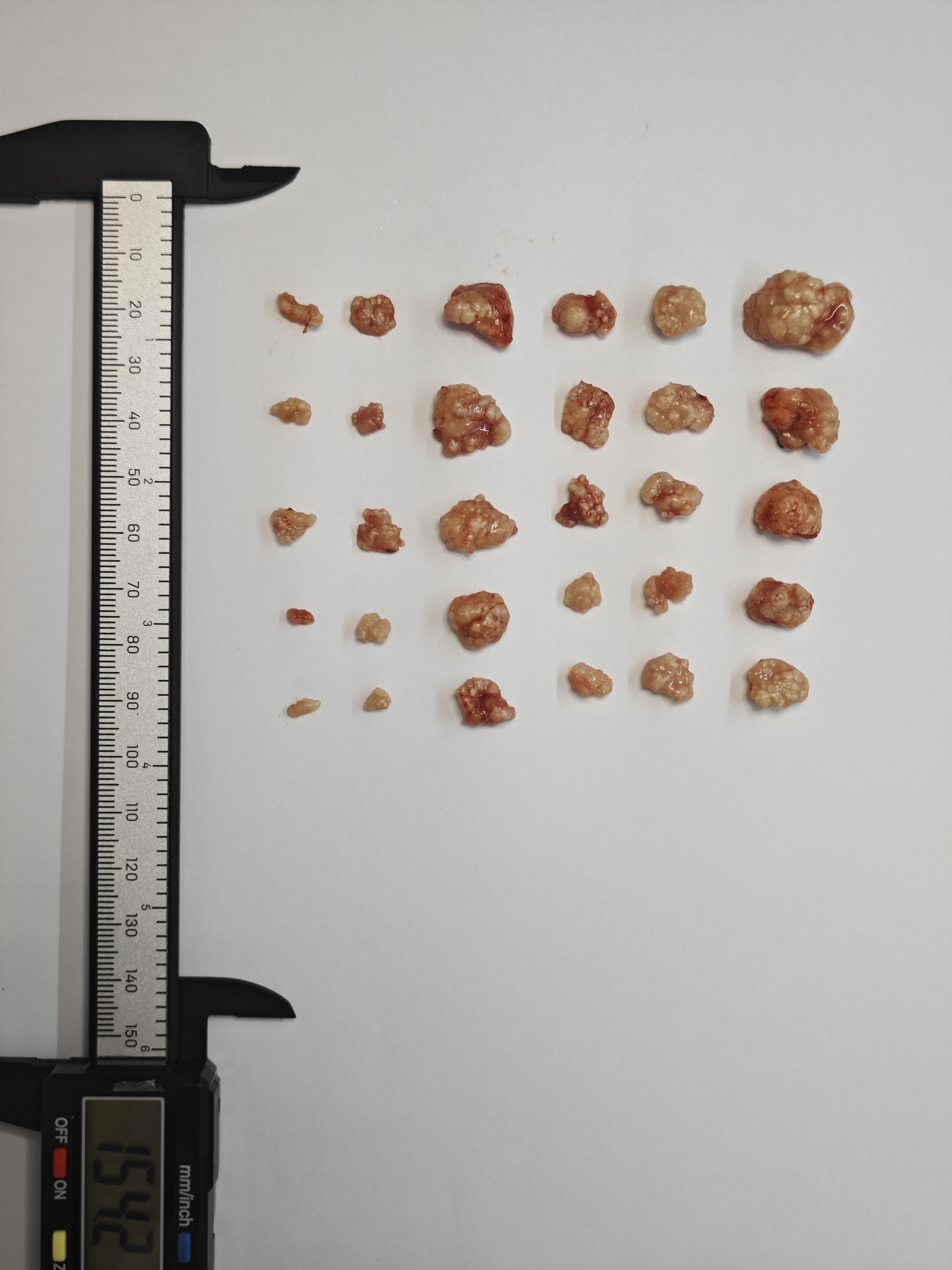

Supplement: Supplementary file 6 — Source data Fig. 5 [file 44319_2026_749_MOESM6_ESM.zip › Figure 5/5H/USP25敲除索拉菲尼 代表性图.jpg]

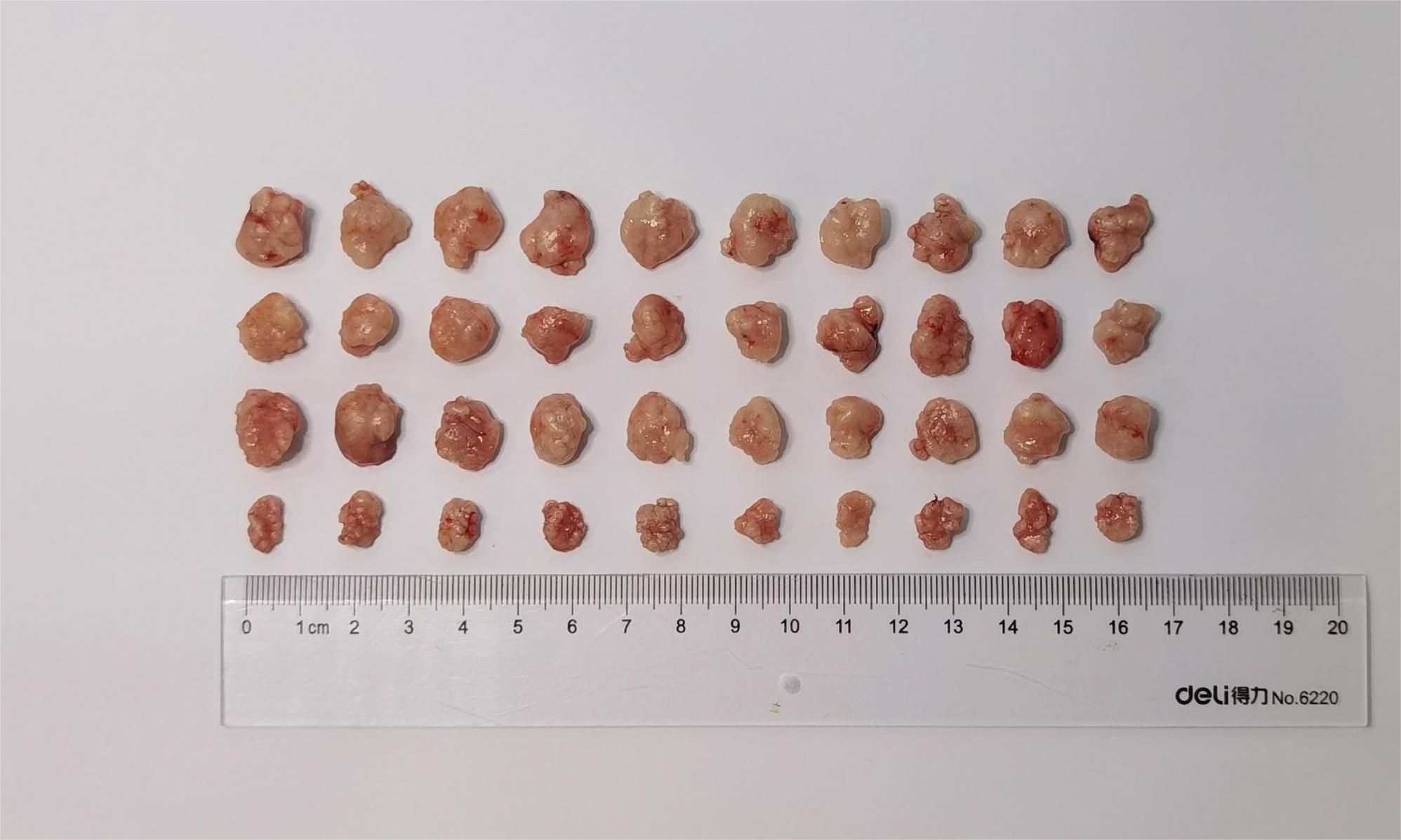

Supplement: Supplementary file 6 — Source data Fig. 5 [file 44319_2026_749_MOESM6_ESM.zip › Figure 5/5L/AZ1 索拉菲尼 代表性图.png]

**Figure 6B**

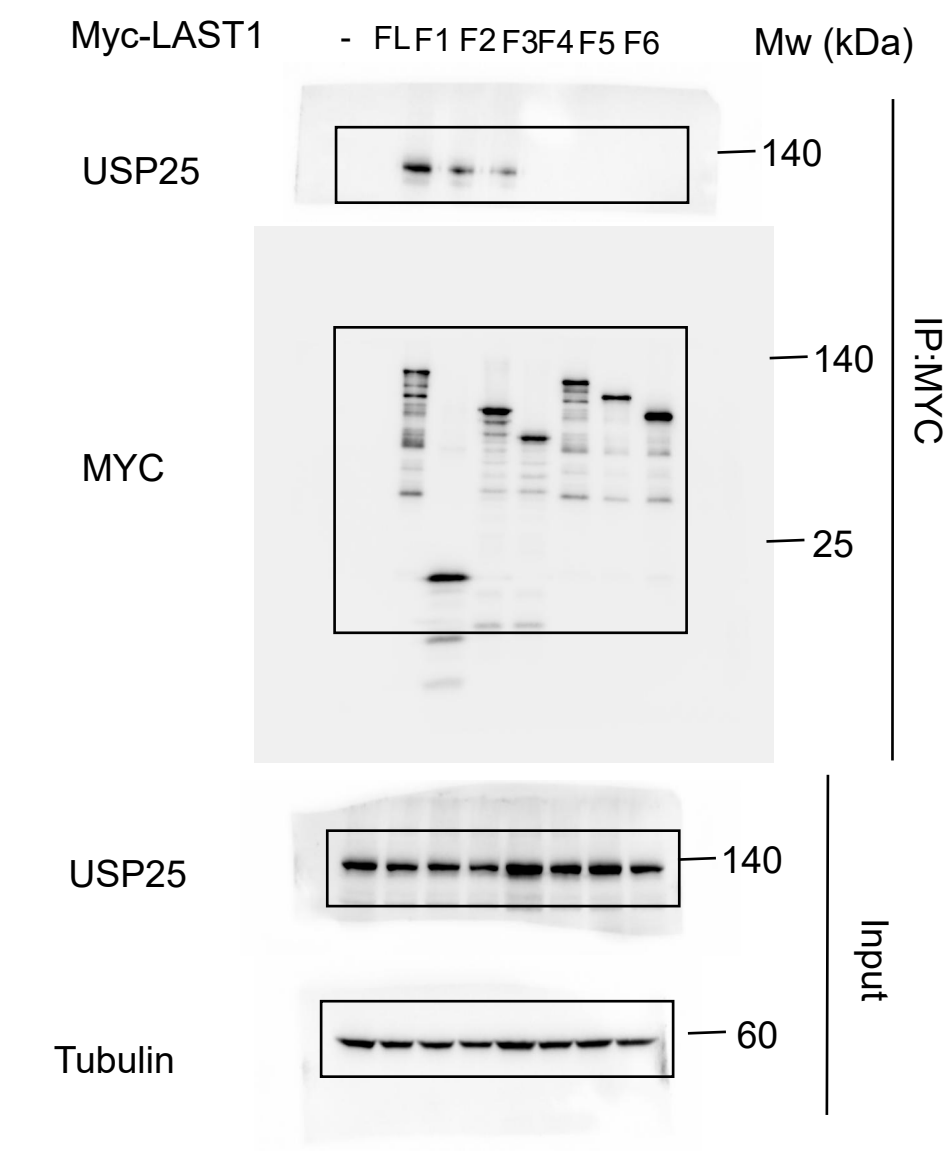

Supplement: Supplementary file 7 — Source data Fig. 6 [file 44319_2026_749_MOESM7_ESM.zip › Figure 6/6B/Western blots 6B.pdf]

Figure 6D

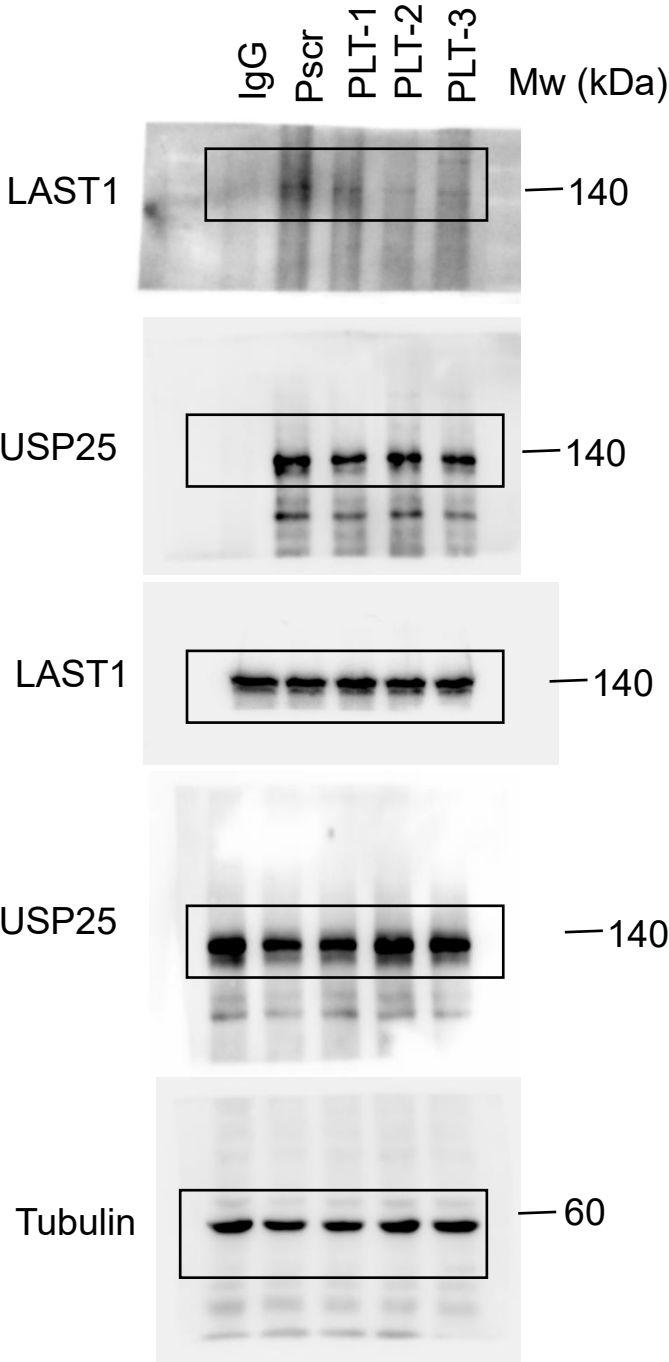

Supplement: Supplementary file 7 — Source data Fig. 6 [file 44319_2026_749_MOESM7_ESM.zip › Figure 6/6D/Western blots 6D.pdf]

Figure 6E

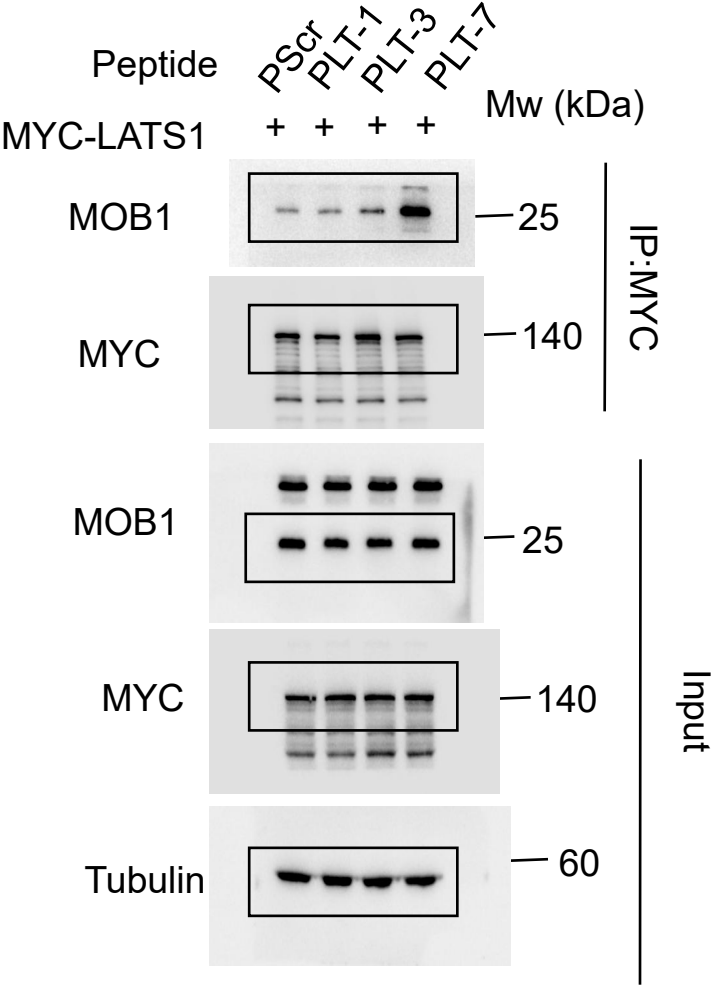

Supplement: Supplementary file 7 — Source data Fig. 6 [file 44319_2026_749_MOESM7_ESM.zip › Figure 6/6E/Western blots 6E.pdf]

Figure 6F

| Peptide   | PScf | PLT-1 | PLT-3 | PLT-7 | Mw (kDa) |
|-----------|------|-------|-------|-------|----------|
| MYC-LATS1 | +    | +     | +     | +     |          |
| His-ub    | +    | +     | +     | +     |          |

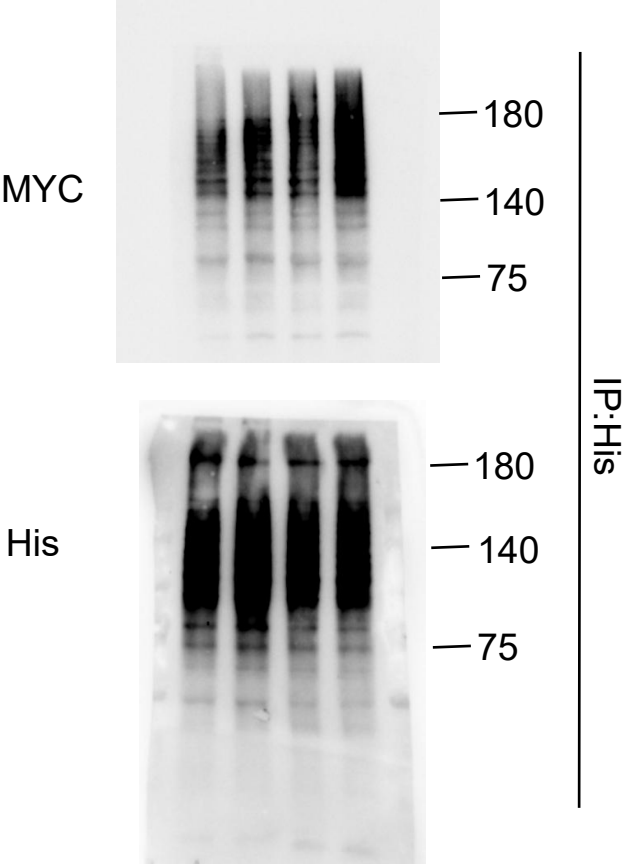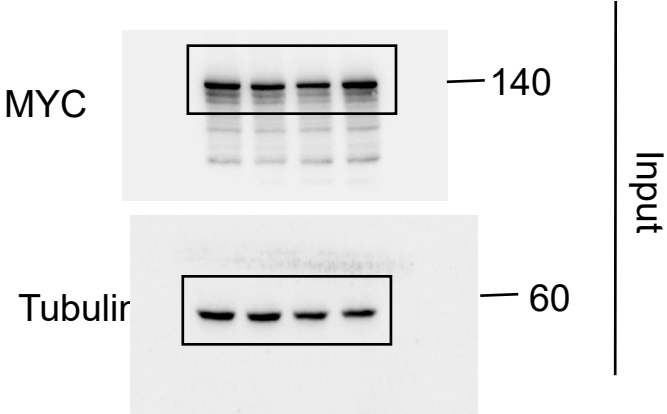

Supplement: Supplementary file 7 — Source data Fig. 6 [file 44319_2026_749_MOESM7_ESM.zip › Figure 6/6F/Western blots 6F.pdf]

Figure 6G

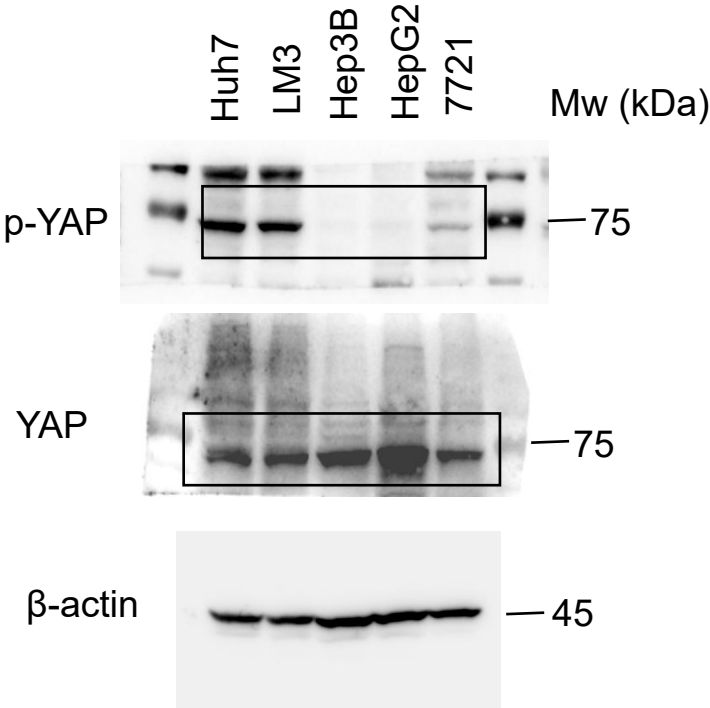

Supplement: Supplementary file 7 — Source data Fig. 6 [file 44319_2026_749_MOESM7_ESM.zip › Figure 6/6G/Western blots 6G.pdf]

Figure 6H

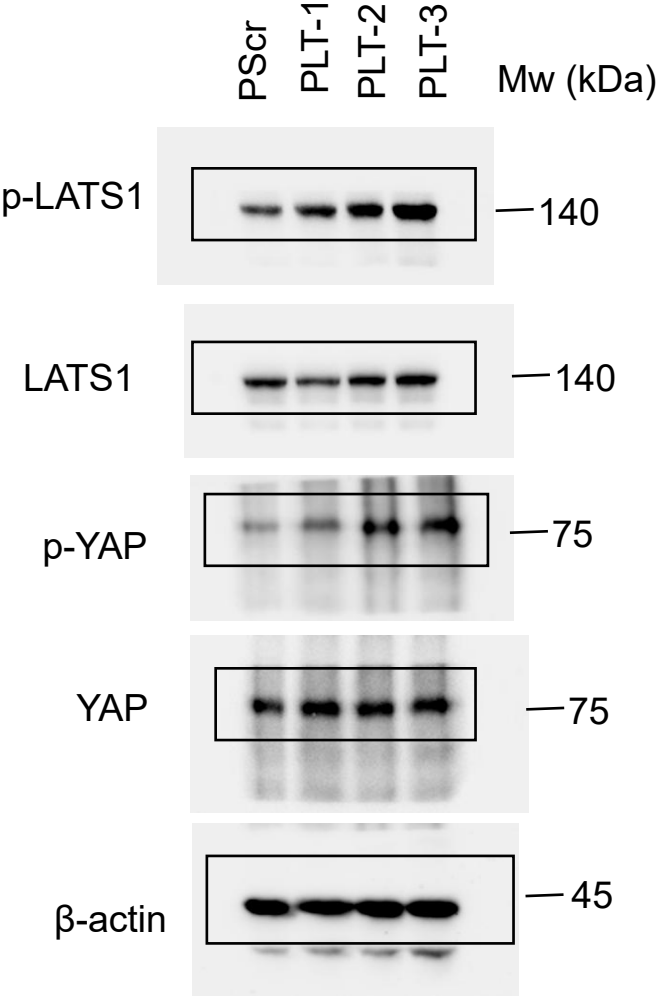

Supplement: Supplementary file 7 — Source data Fig. 6 [file 44319_2026_749_MOESM7_ESM.zip › Figure 6/6H/Western blots 6H.pdf]

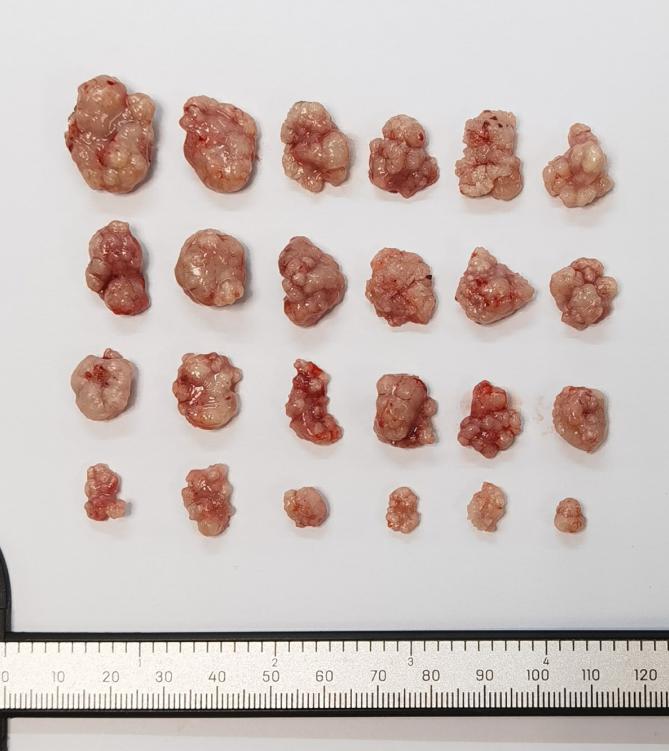

Supplement: Supplementary file 7 — Source data Fig. 6 [file 44319_2026_749_MOESM7_ESM.zip › Figure 6/6J/tumor.jpg]

Figure 7A

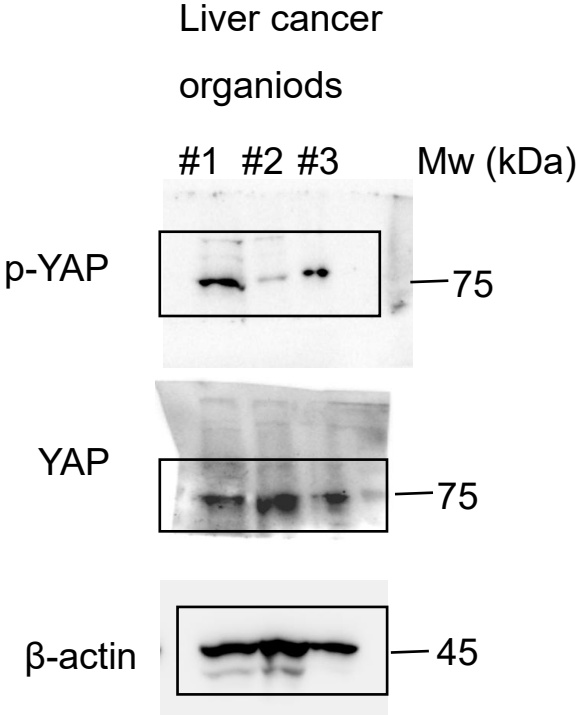

Supplement: Supplementary file 8 — Source data Fig. 7 [file 44319_2026_749_MOESM8_ESM.zip › Figure 7/7A/Western blots 7A.pdf]

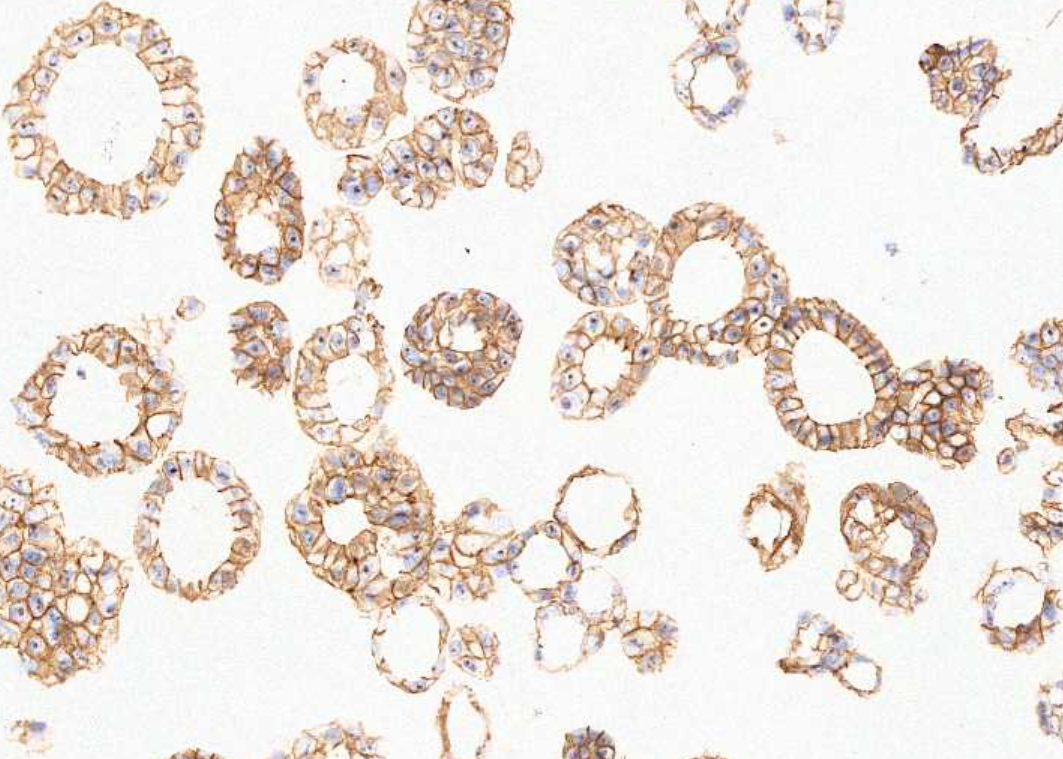

Supplement: Supplementary file 8 — Source data Fig. 7 [file 44319_2026_749_MOESM8_ESM.zip › Figure 7/7B/#1 E-cadherin.tif]

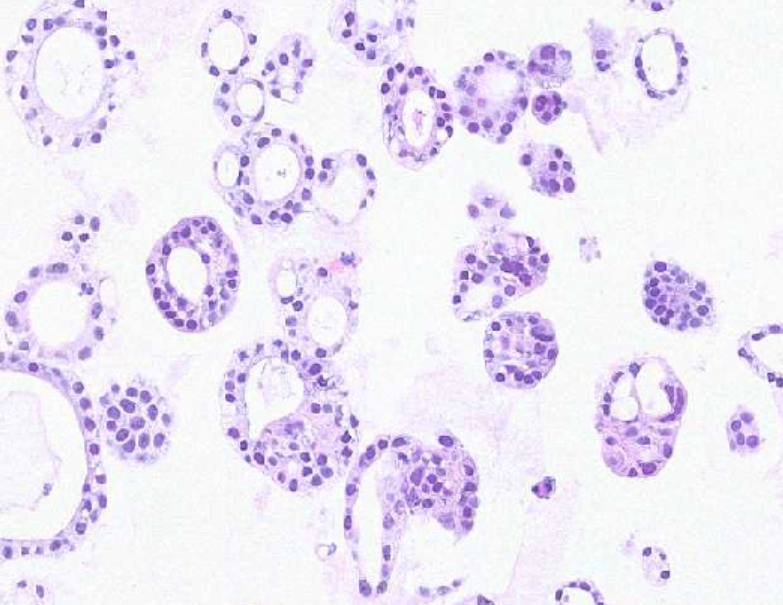

Supplement: Supplementary file 8 — Source data Fig. 7 [file 44319_2026_749_MOESM8_ESM.zip › Figure 7/7B/#1 HE.tif]

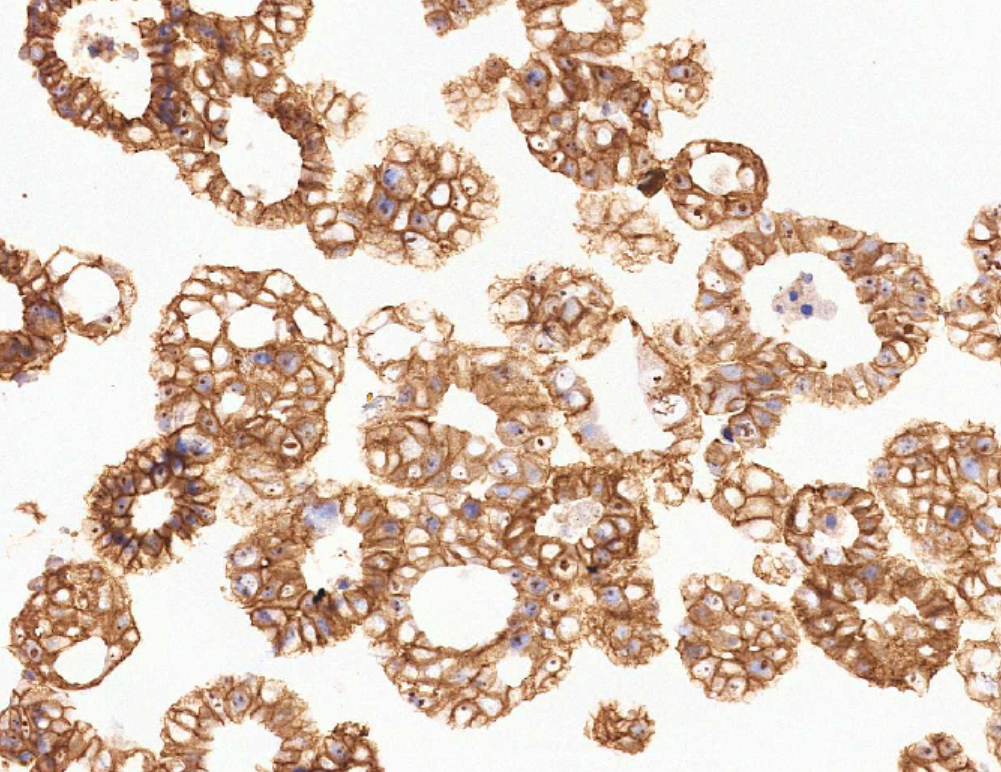

Supplement: Supplementary file 8 — Source data Fig. 7 [file 44319_2026_749_MOESM8_ESM.zip › Figure 7/7B/#2 E-cadherin.tif]

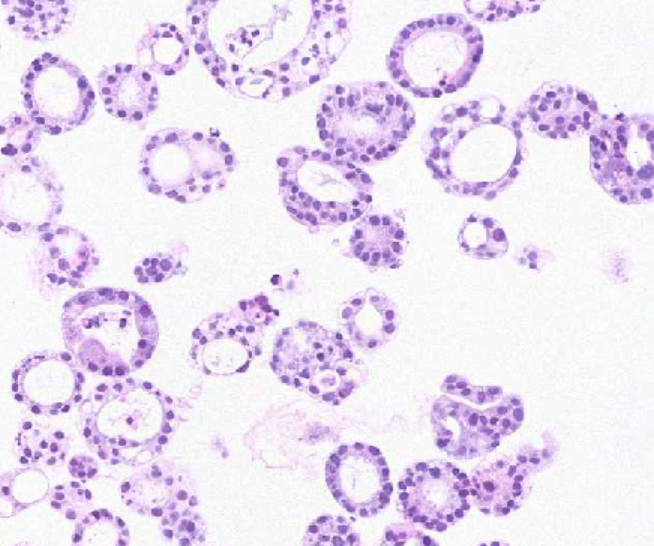

Supplement: Supplementary file 8 — Source data Fig. 7 [file 44319_2026_749_MOESM8_ESM.zip › Figure 7/7B/#2 HE.tif]

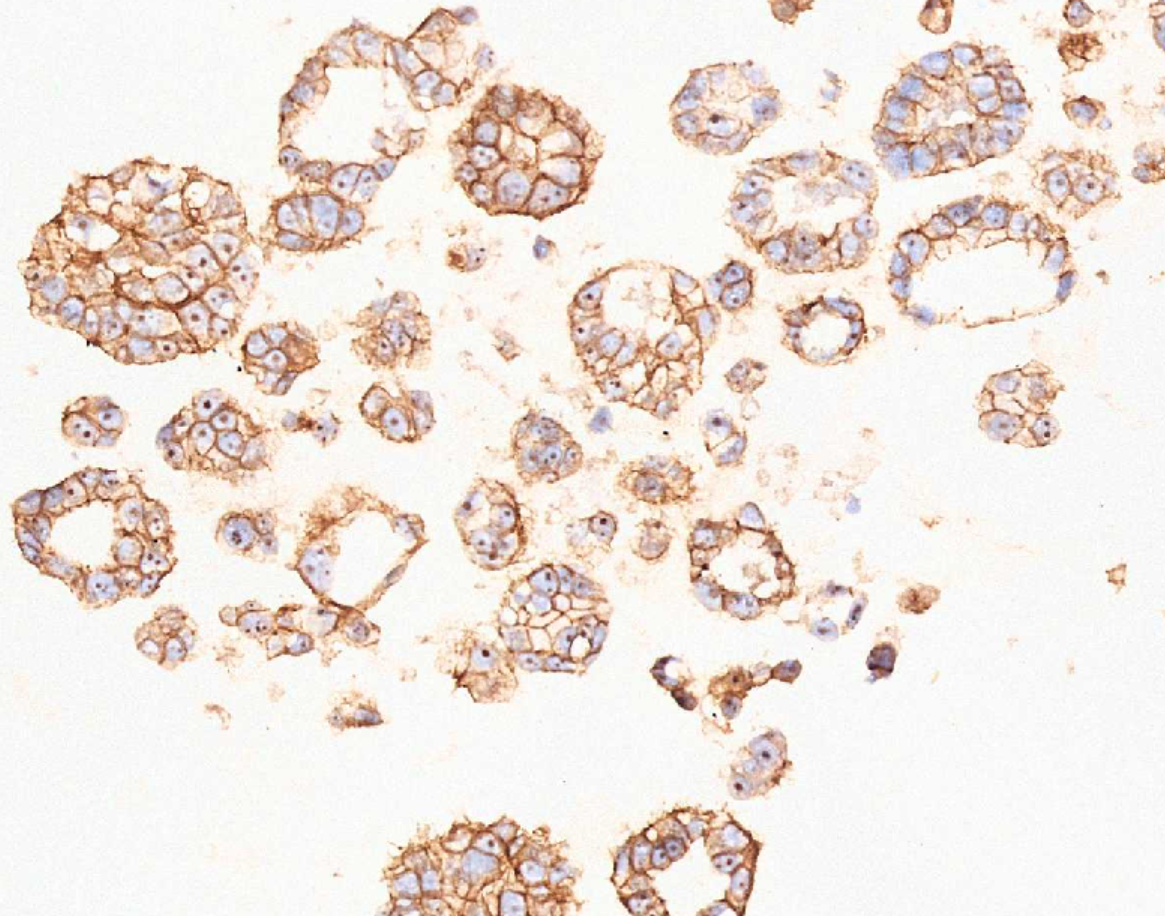

Supplement: Supplementary file 8 — Source data Fig. 7 [file 44319_2026_749_MOESM8_ESM.zip › Figure 7/7B/#3 E-cadherin.tif]

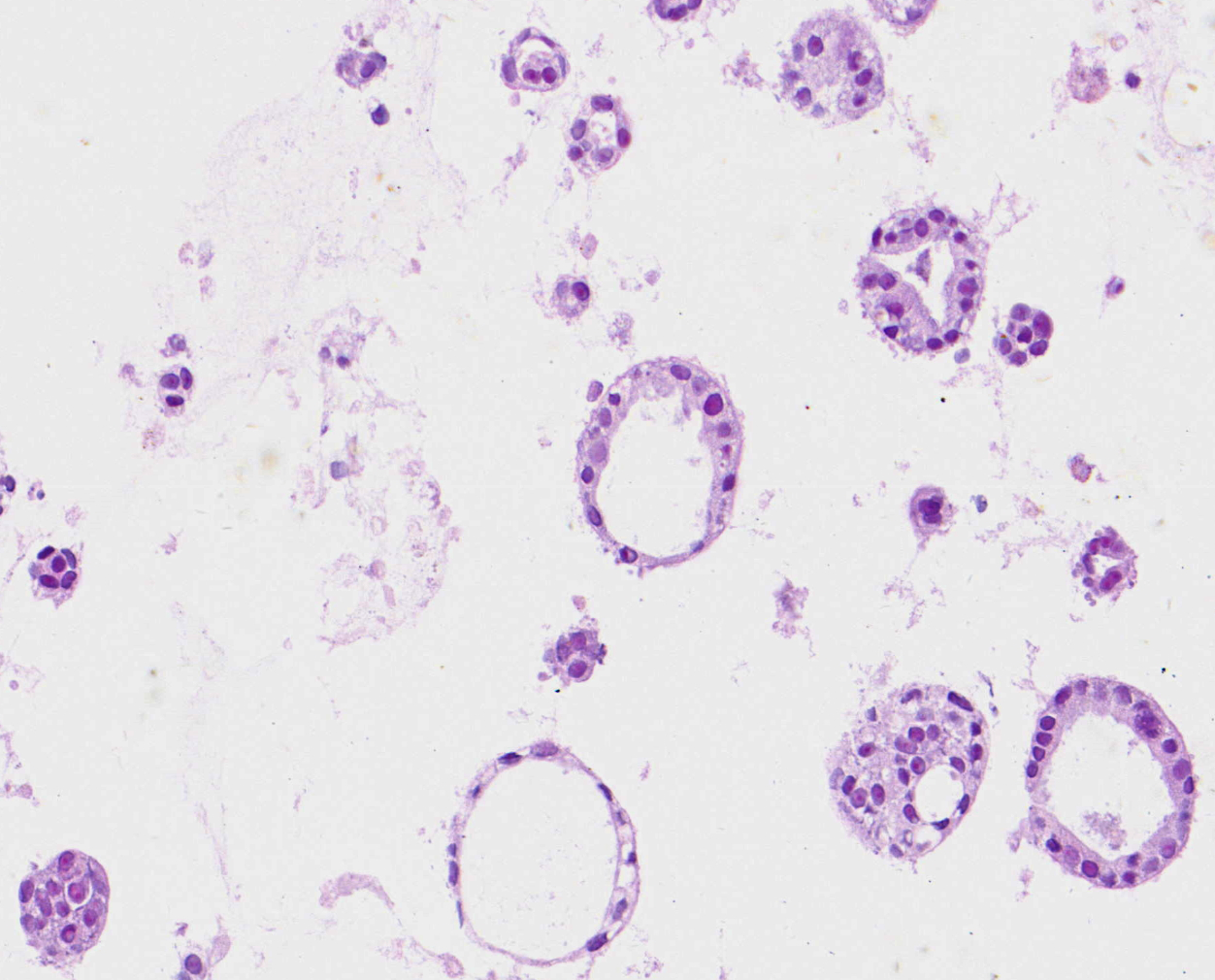

Supplement: Supplementary file 8 — Source data Fig. 7 [file 44319_2026_749_MOESM8_ESM.zip › Figure 7/7B/#3 HE.tif]

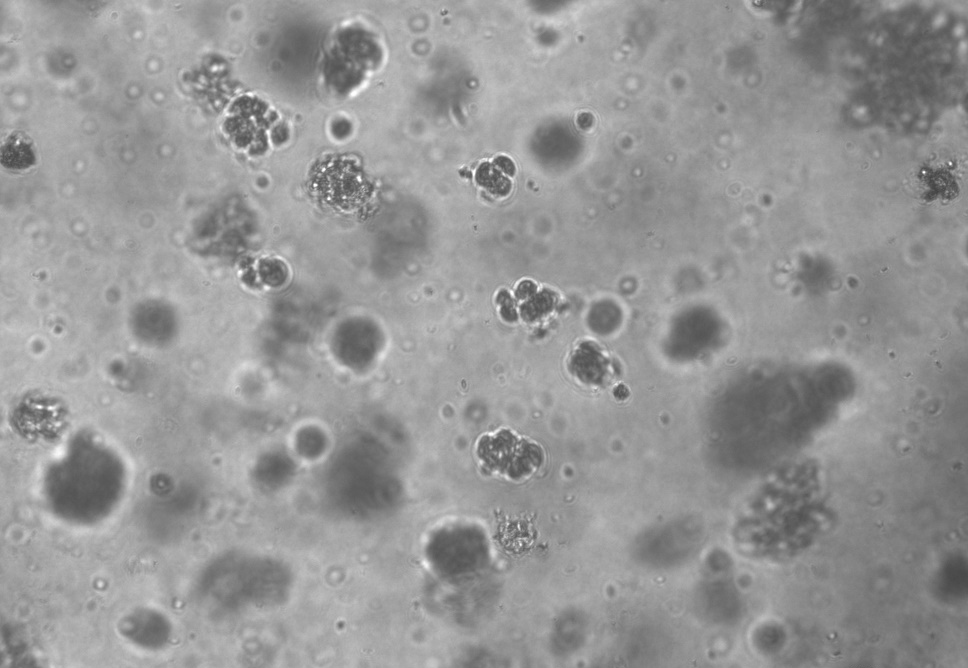

Supplement: Supplementary file 8 — Source data Fig. 7 [file 44319_2026_749_MOESM8_ESM.zip › Figure 7/7C/PLT-3+sorafenib.jpg]

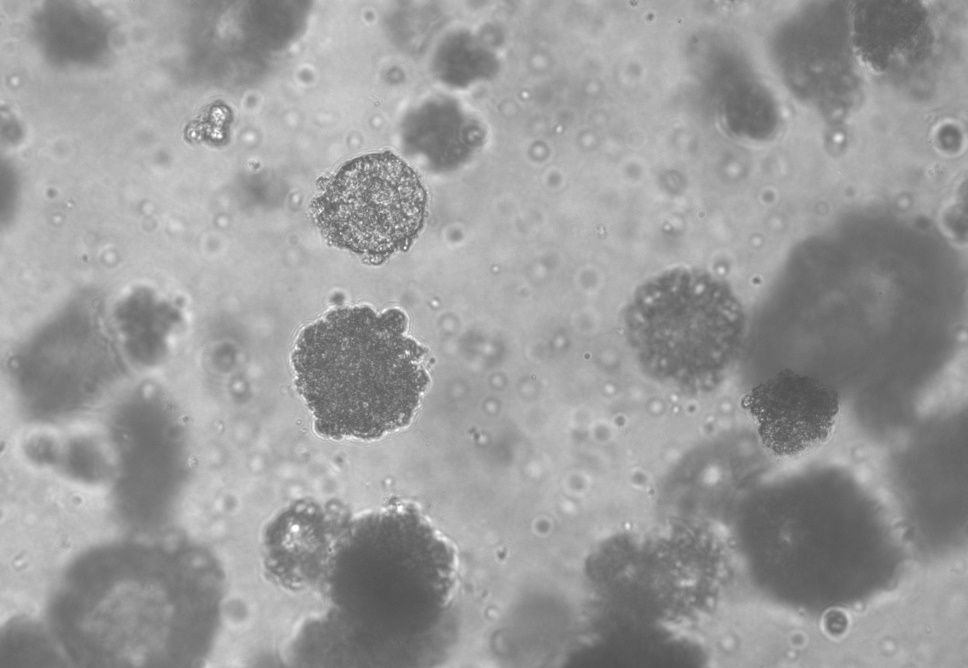

Supplement: Supplementary file 8 — Source data Fig. 7 [file 44319_2026_749_MOESM8_ESM.zip › Figure 7/7C/PLT-3.jpg]

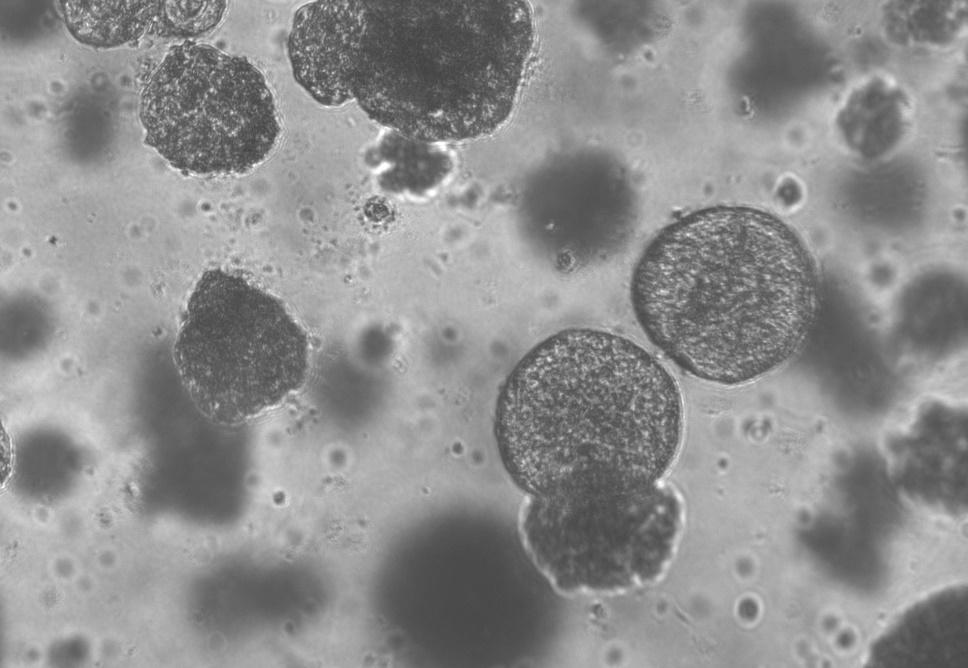

Supplement: Supplementary file 8 — Source data Fig. 7 [file 44319_2026_749_MOESM8_ESM.zip › Figure 7/7C/PScr.jpg]

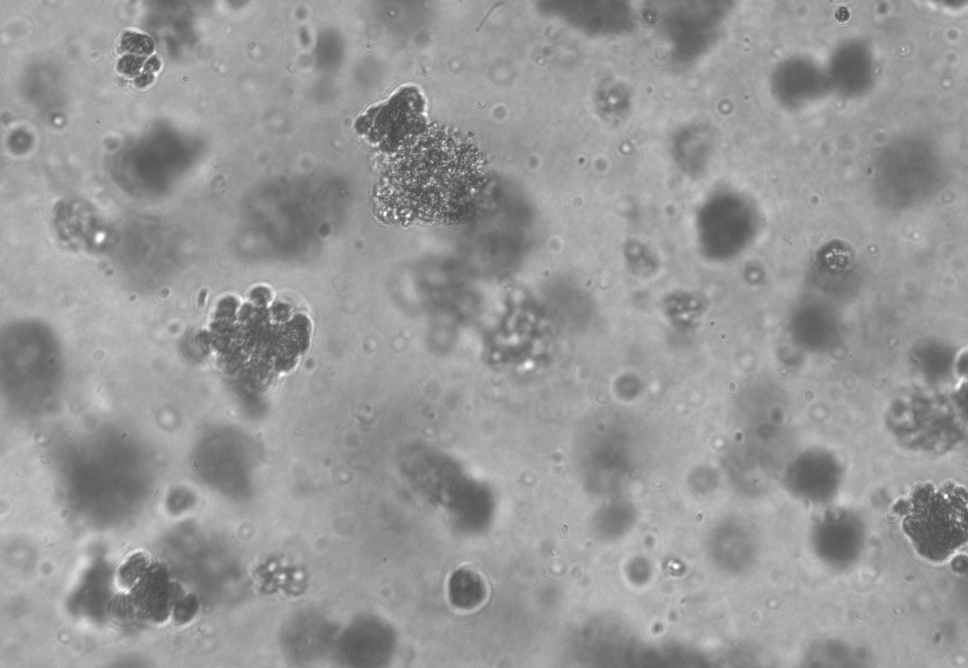

Supplement: Supplementary file 8 — Source data Fig. 7 [file 44319_2026_749_MOESM8_ESM.zip › Figure 7/7C/sorafenib.jpg]

Figure 7E

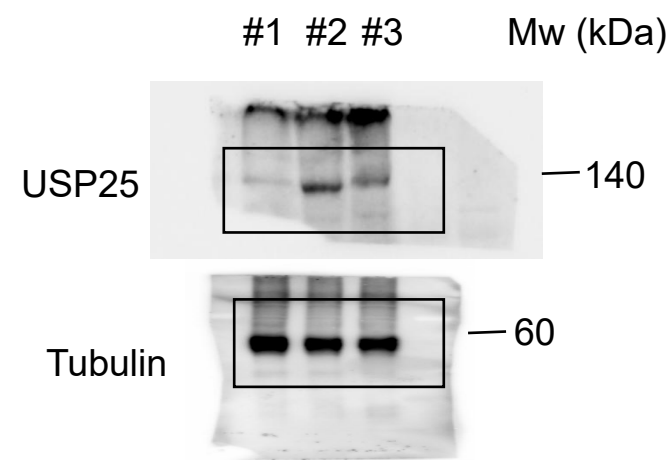

Supplement: Supplementary file 8 — Source data Fig. 7 [file 44319_2026_749_MOESM8_ESM.zip › Figure 7/7E/Western blots 7E.pdf]

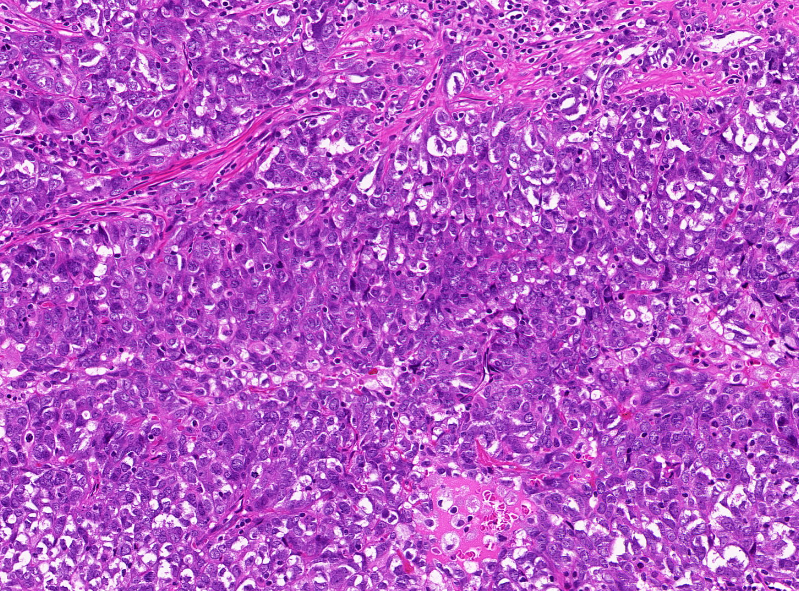

Supplement: Supplementary file 8 — Source data Fig. 7 [file 44319_2026_749_MOESM8_ESM.zip › Figure 7/7F/P1 HE.tif]

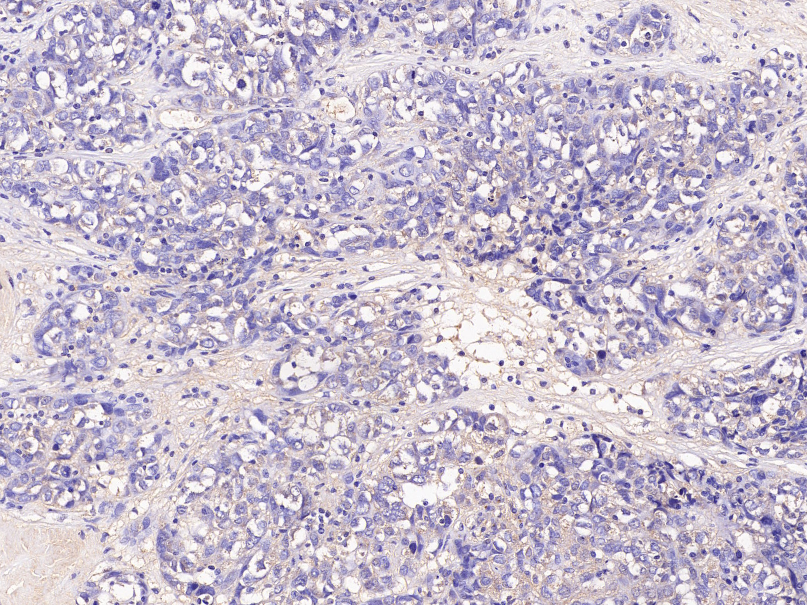

Supplement: Supplementary file 8 — Source data Fig. 7 [file 44319_2026_749_MOESM8_ESM.zip › Figure 7/7F/P1 USP25.tif]

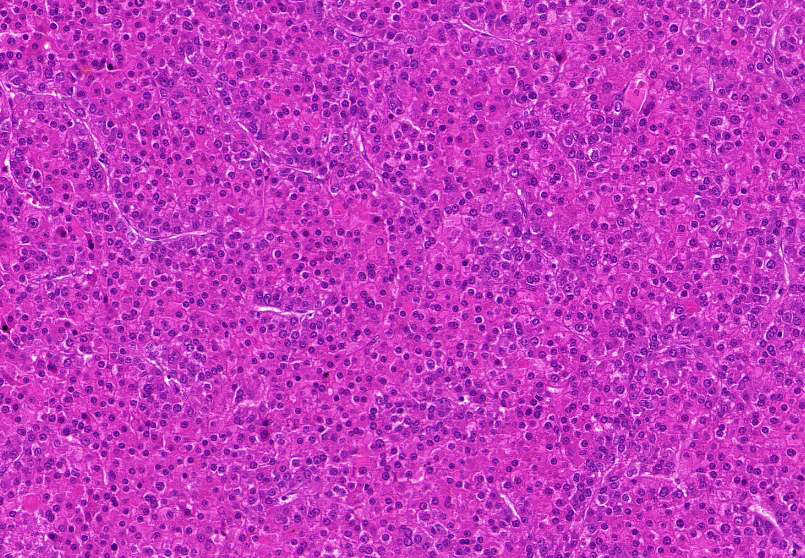

Supplement: Supplementary file 8 — Source data Fig. 7 [file 44319_2026_749_MOESM8_ESM.zip › Figure 7/7F/P2 HE.tif]

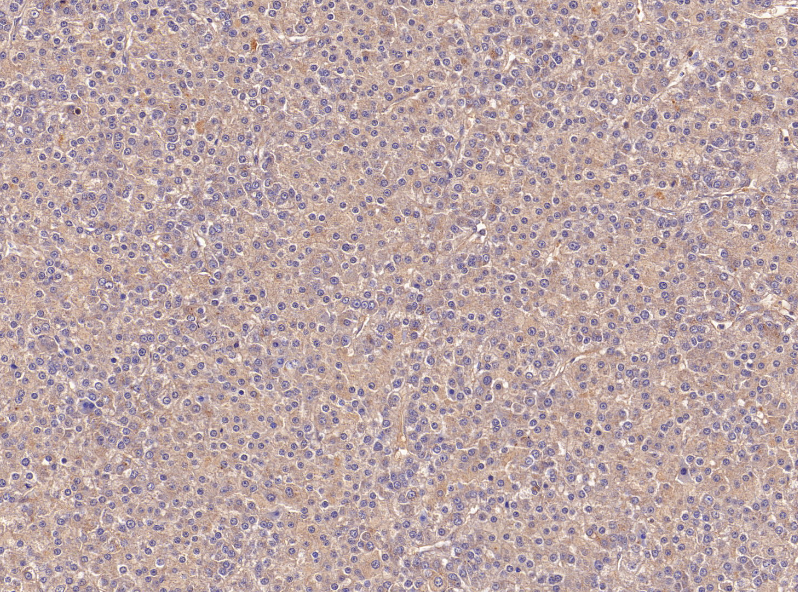

Supplement: Supplementary file 8 — Source data Fig. 7 [file 44319_2026_749_MOESM8_ESM.zip › Figure 7/7F/P2 USP25.tif]

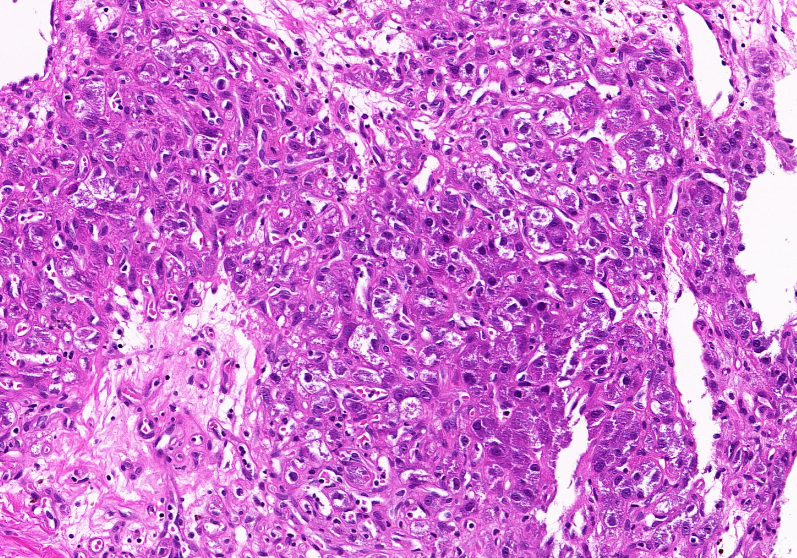

Supplement: Supplementary file 8 — Source data Fig. 7 [file 44319_2026_749_MOESM8_ESM.zip › Figure 7/7F/P3 HE.tif]

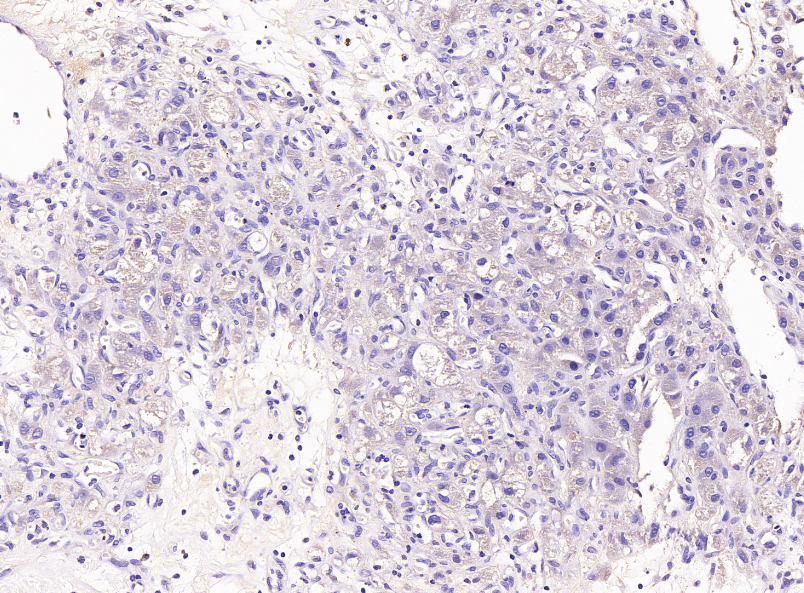

Supplement: Supplementary file 8 — Source data Fig. 7 [file 44319_2026_749_MOESM8_ESM.zip › Figure 7/7F/P3 USP25.tif]

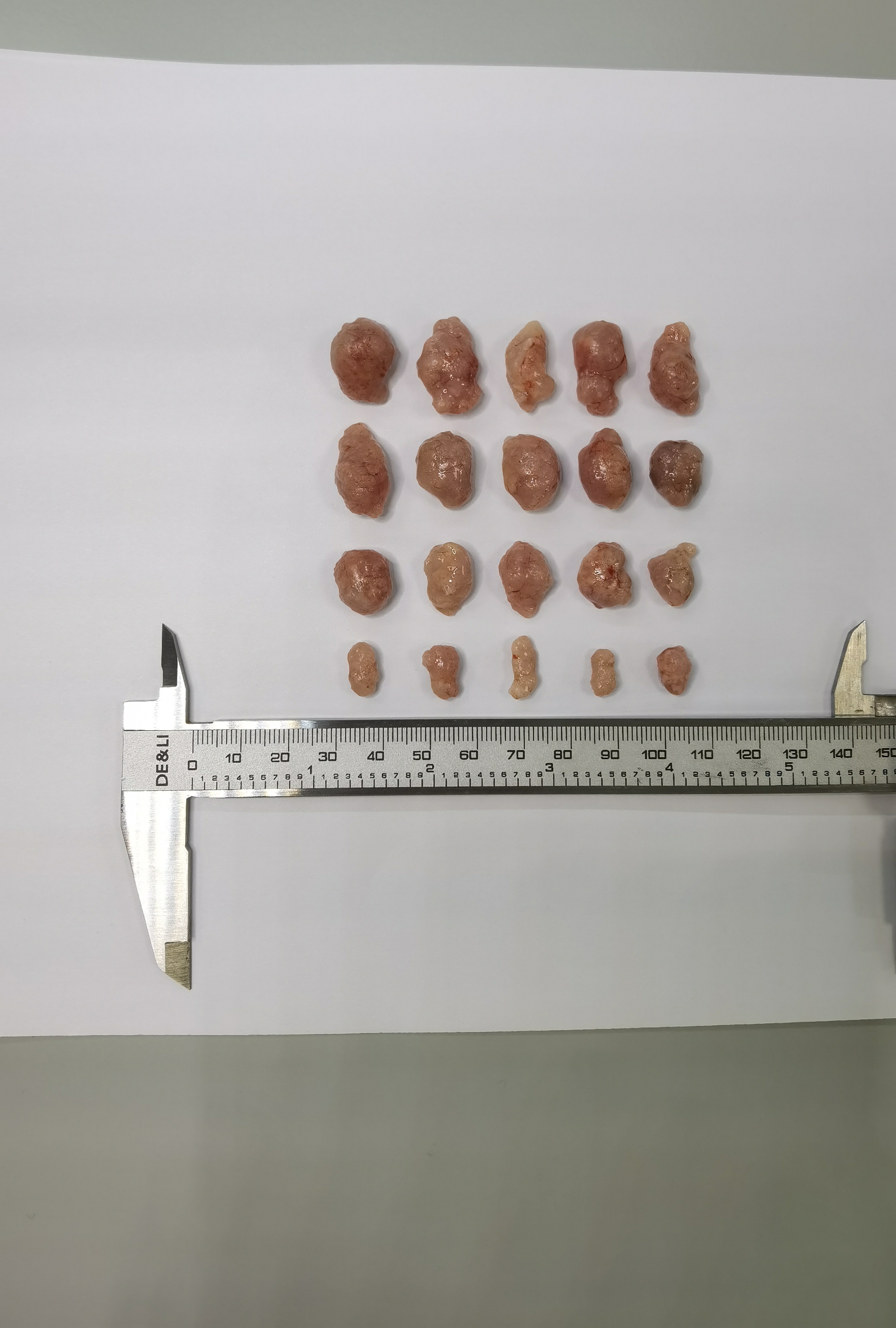

Supplement: Supplementary file 8 — Source data Fig. 7 [file 44319_2026_749_MOESM8_ESM.zip › Figure 7/7G/IMG_20250321_173406.jpg]

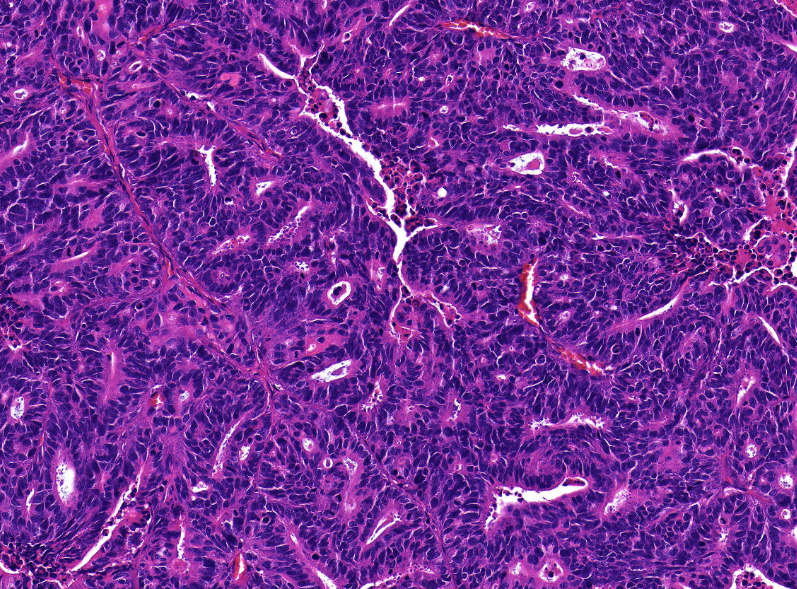

Supplement: Supplementary file 8 — Source data Fig. 7 [file 44319_2026_749_MOESM8_ESM.zip › Figure 7/7J/PLT-3 HE.tif]

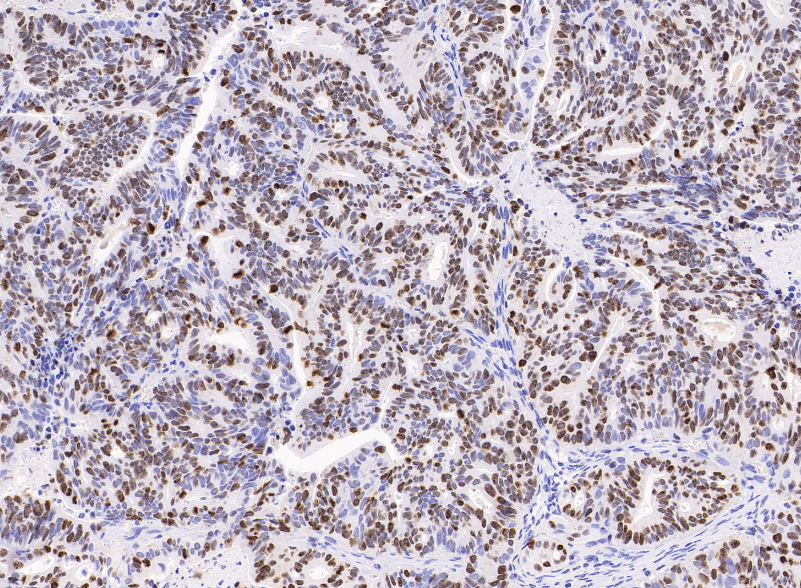

Supplement: Supplementary file 8 — Source data Fig. 7 [file 44319_2026_749_MOESM8_ESM.zip › Figure 7/7J/PLT-3 Ki67.tif]

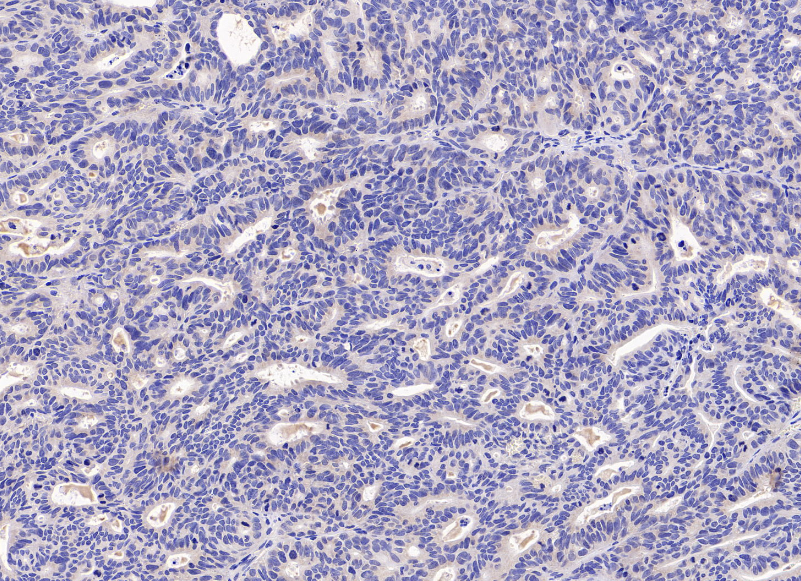

Supplement: Supplementary file 8 — Source data Fig. 7 [file 44319_2026_749_MOESM8_ESM.zip › Figure 7/7J/PLT-3 p-YAP.tif]

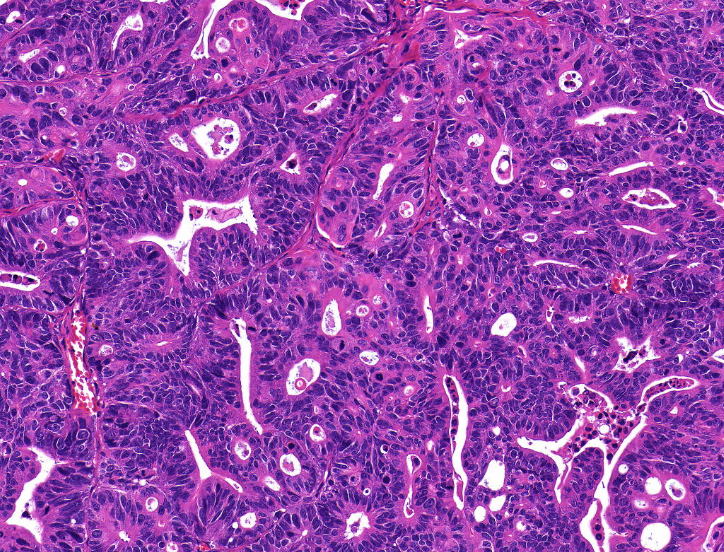

Supplement: Supplementary file 8 — Source data Fig. 7 [file 44319_2026_749_MOESM8_ESM.zip › Figure 7/7J/PLT-3 Sorafenib HE.tif]

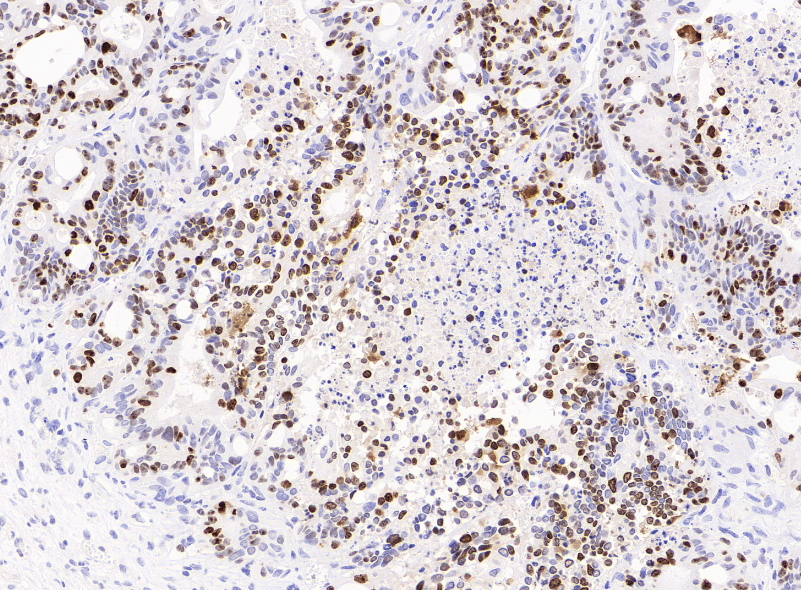

Supplement: Supplementary file 8 — Source data Fig. 7 [file 44319_2026_749_MOESM8_ESM.zip › Figure 7/7J/PLT3+Sorafenib Ki67.tif]

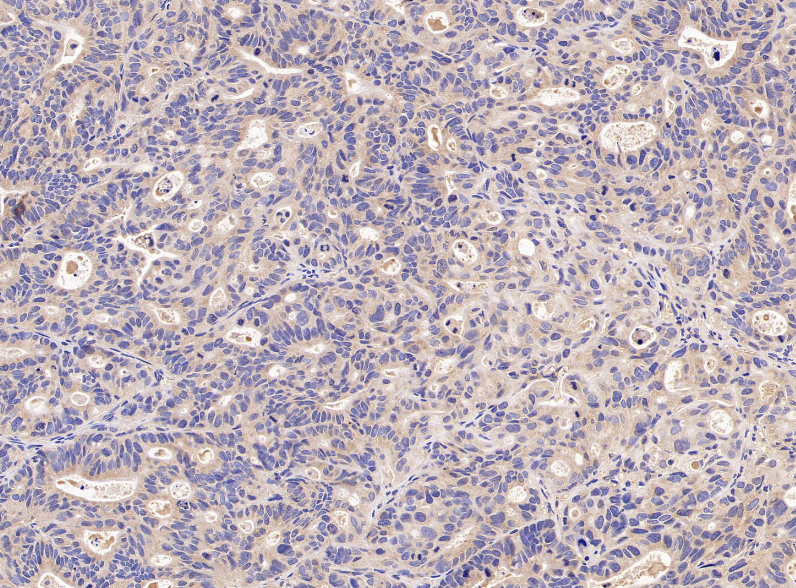

Supplement: Supplementary file 8 — Source data Fig. 7 [file 44319_2026_749_MOESM8_ESM.zip › Figure 7/7J/PLT3+Sorafenib pYAP.tif]

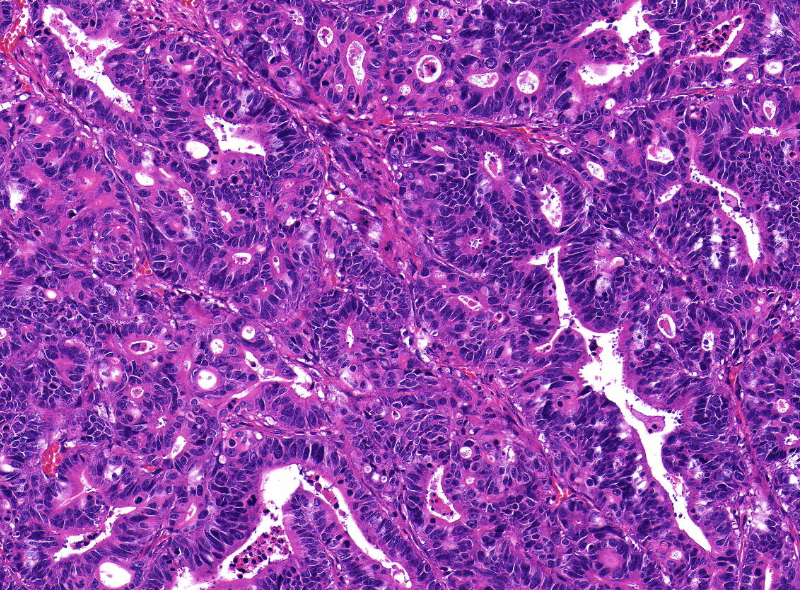

Supplement: Supplementary file 8 — Source data Fig. 7 [file 44319_2026_749_MOESM8_ESM.zip › Figure 7/7J/pscr HE.tif]

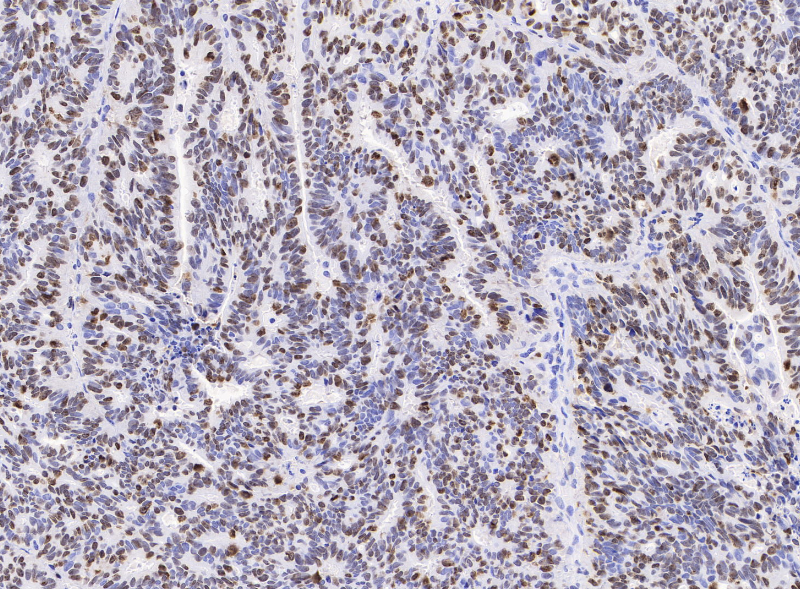

Supplement: Supplementary file 8 — Source data Fig. 7 [file 44319_2026_749_MOESM8_ESM.zip › Figure 7/7J/pscr Ki67.tif]

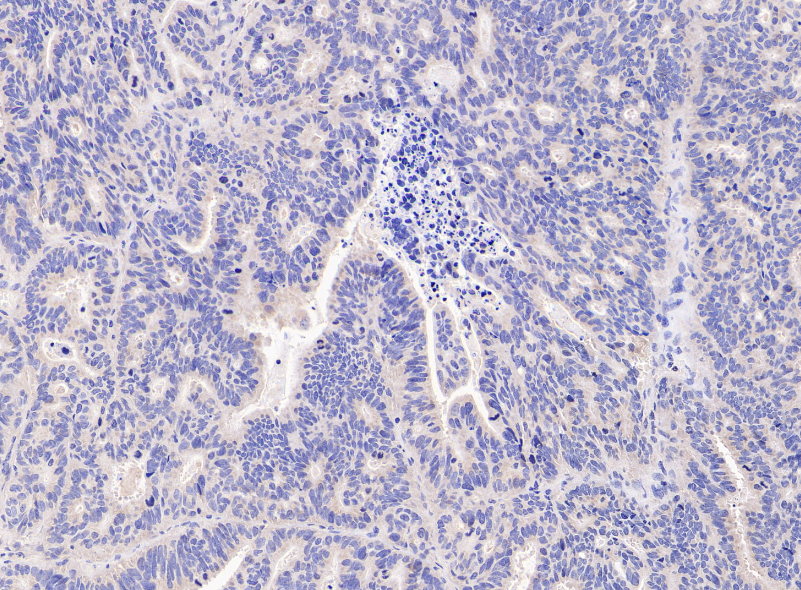

Supplement: Supplementary file 8 — Source data Fig. 7 [file 44319_2026_749_MOESM8_ESM.zip › Figure 7/7J/pscr P-YAP.tif]

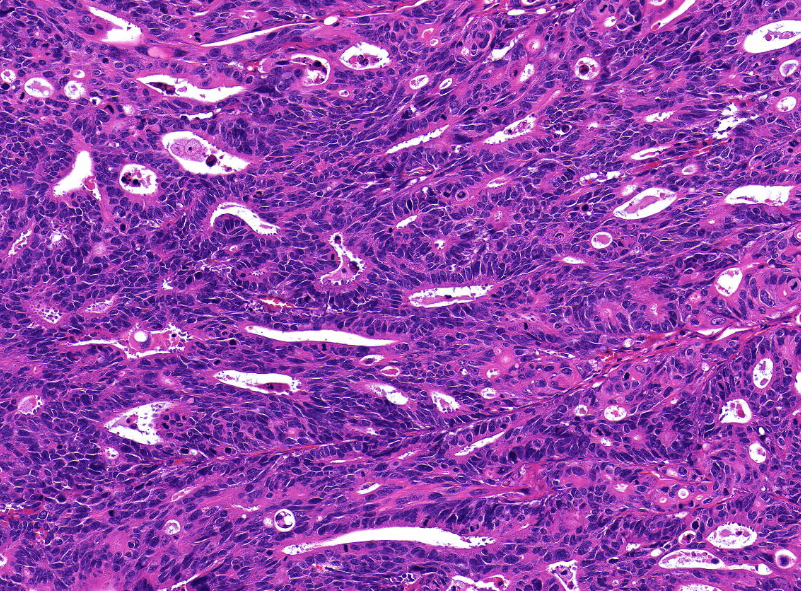

Supplement: Supplementary file 8 — Source data Fig. 7 [file 44319_2026_749_MOESM8_ESM.zip › Figure 7/7J/Sorafenib HE.tif]

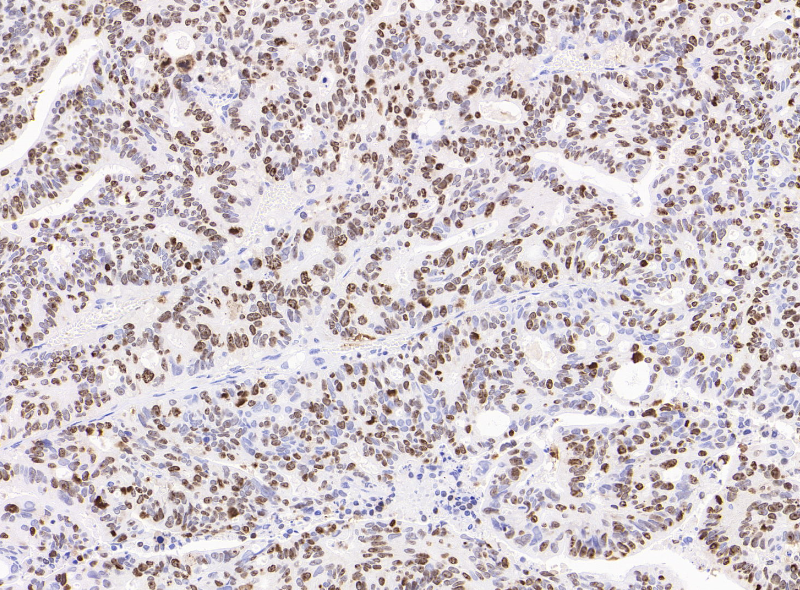

Supplement: Supplementary file 8 — Source data Fig. 7 [file 44319_2026_749_MOESM8_ESM.zip › Figure 7/7J/Sorafenib Ki67.tif]

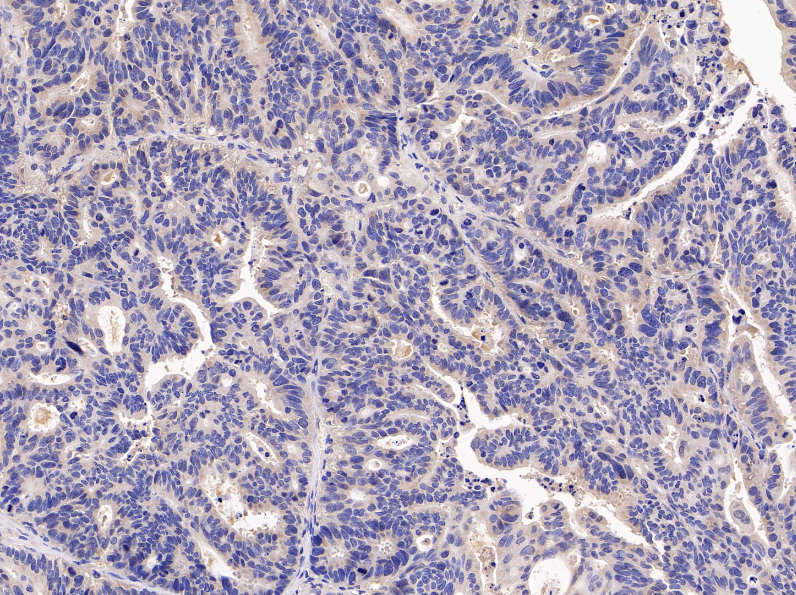

Supplement: Supplementary file 8 — Source data Fig. 7 [file 44319_2026_749_MOESM8_ESM.zip › Figure 7/7J/Sorafenib p-YAP.tif]

## Figure EV1A

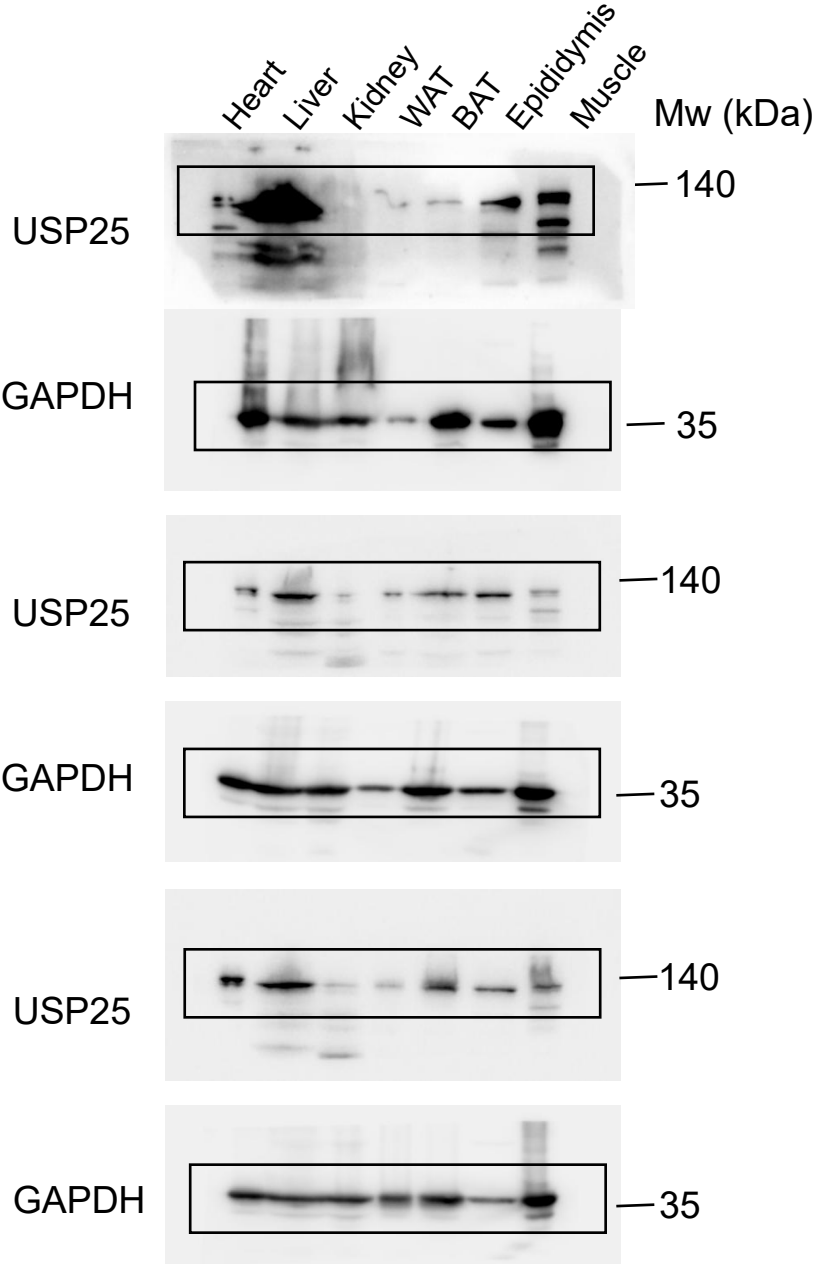

Supplement: Supplementary file 9 — Figure EV1 Source Data [file 44319_2026_749_MOESM9_ESM.zip › Figure EV1/EV 1A/Western blots EV1A.pdf]

Figure EV1B

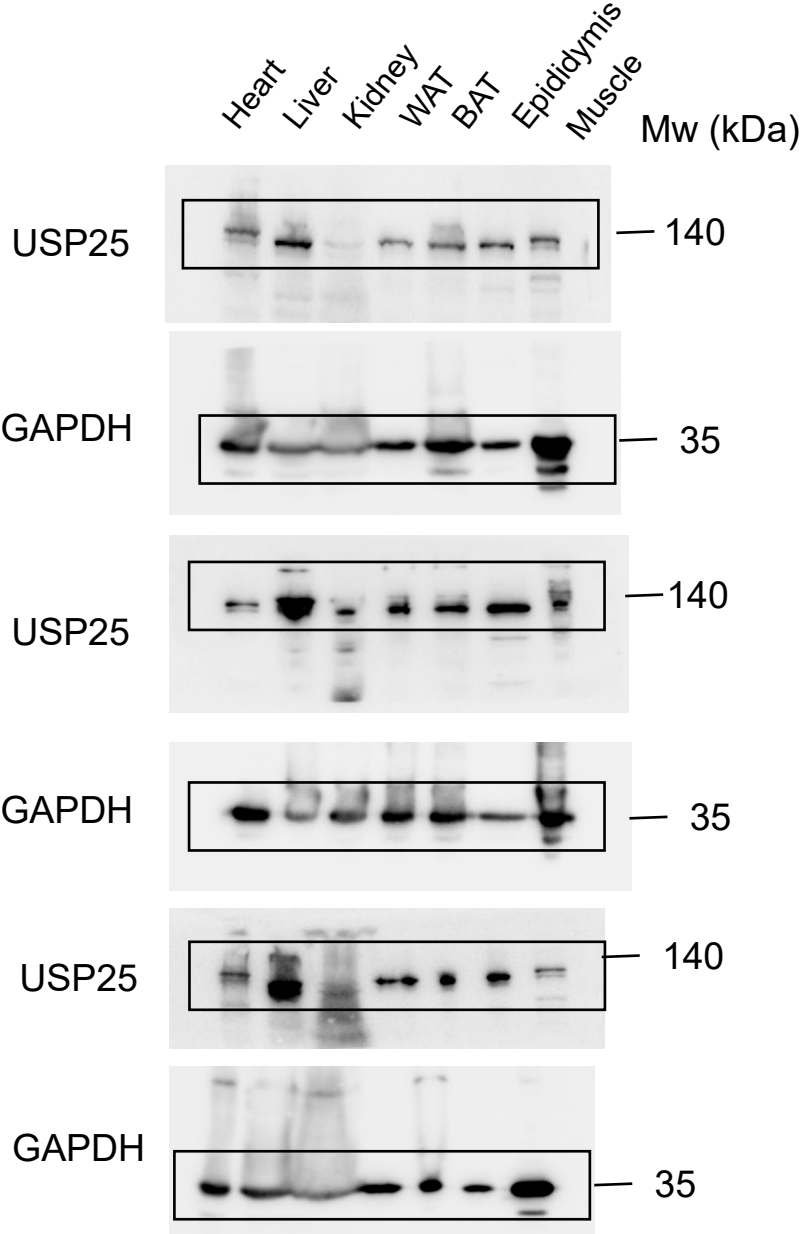

Supplement: Supplementary file 9 — Figure EV1 Source Data [file 44319_2026_749_MOESM9_ESM.zip › Figure EV1/EV 1B/Western blots EV1B.pdf]

**Figure EV1E**

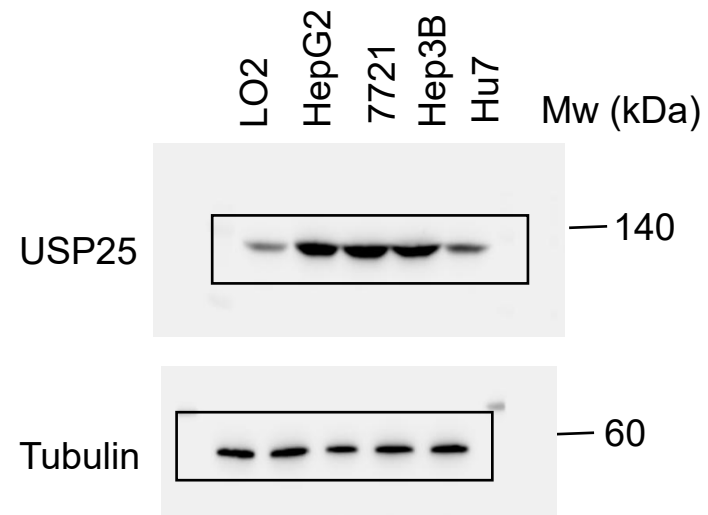

Supplement: Supplementary file 9 — Figure EV1 Source Data [file 44319_2026_749_MOESM9_ESM.zip › Figure EV1/EV 1E/Western blots EV1E.pdf]

**Figure EV1F**

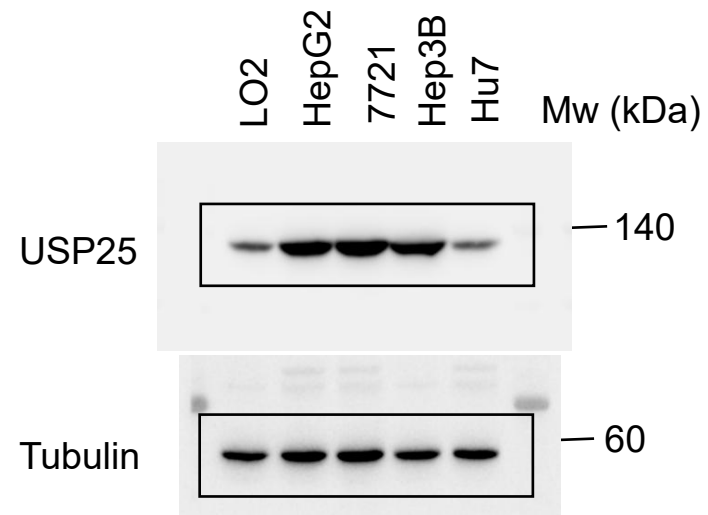

Supplement: Supplementary file 9 — Figure EV1 Source Data [file 44319_2026_749_MOESM9_ESM.zip › Figure EV1/EV 1F/Western blots EV1F.pdf]

**Figure. EV1H**

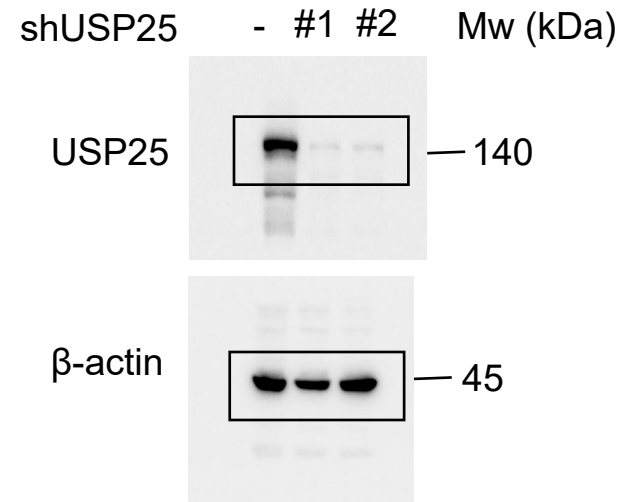

Supplement: Supplementary file 9 — Figure EV1 Source Data [file 44319_2026_749_MOESM9_ESM.zip › Figure EV1/EV 1H/Western blots EV1H.pdf]

**Figure EV1I**

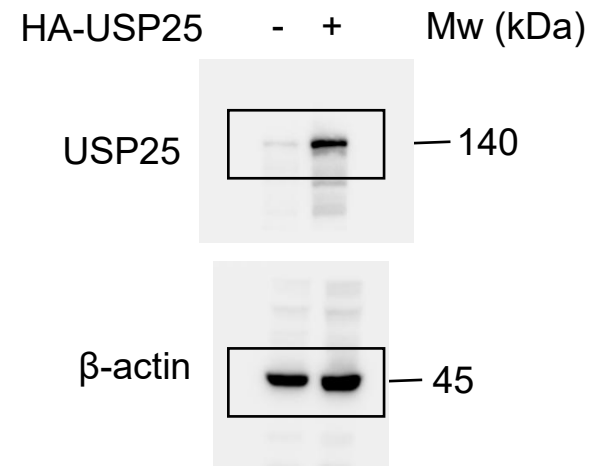

Supplement: Supplementary file 9 — Figure EV1 Source Data [file 44319_2026_749_MOESM9_ESM.zip › Figure EV1/EV 1I/Western blots EV1I.pdf]

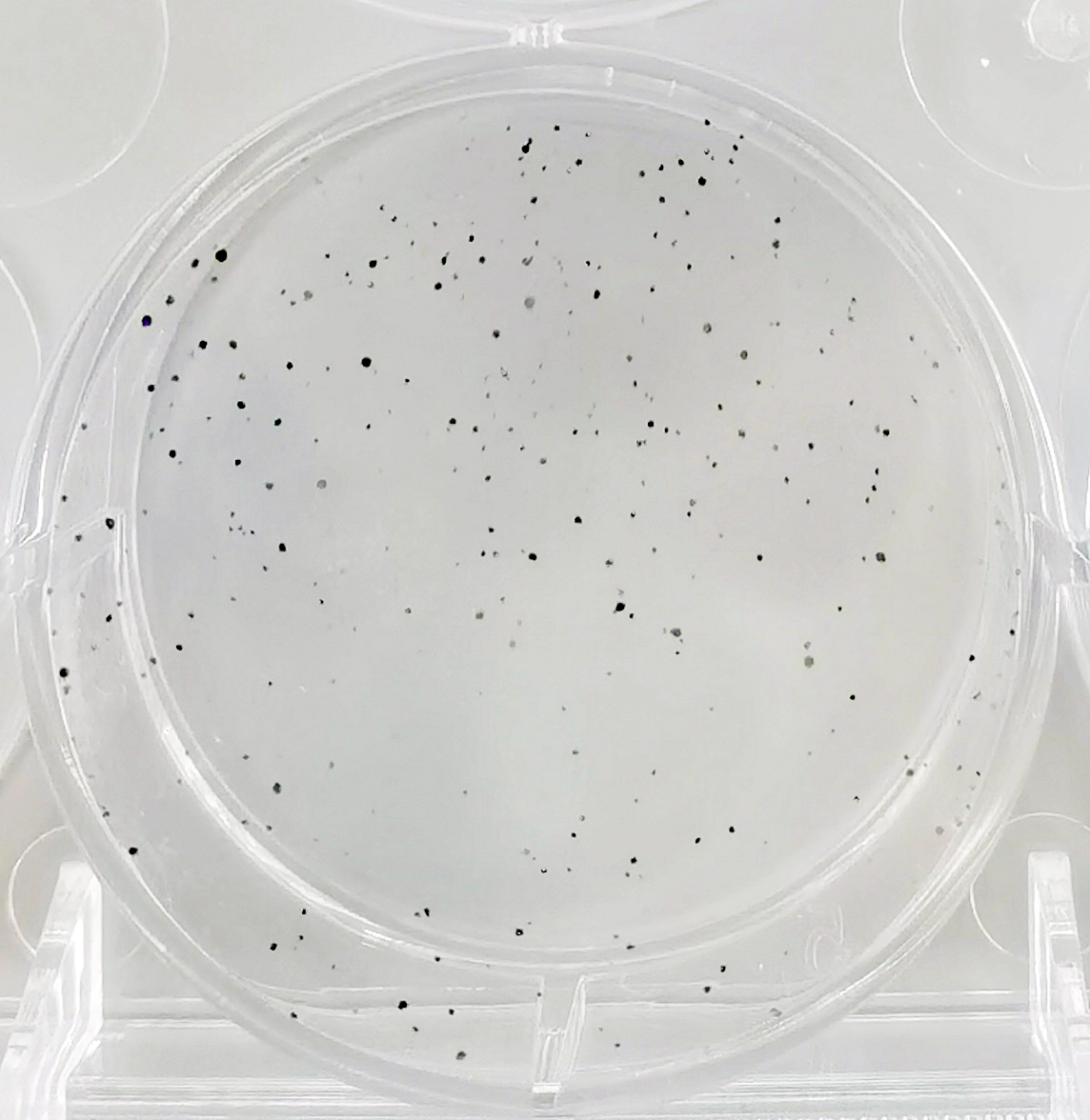

Supplement: Supplementary file 9 — Figure EV1 Source Data [file 44319_2026_749_MOESM9_ESM.zip › Figure EV1/EV 1L/25SH1.jpg]

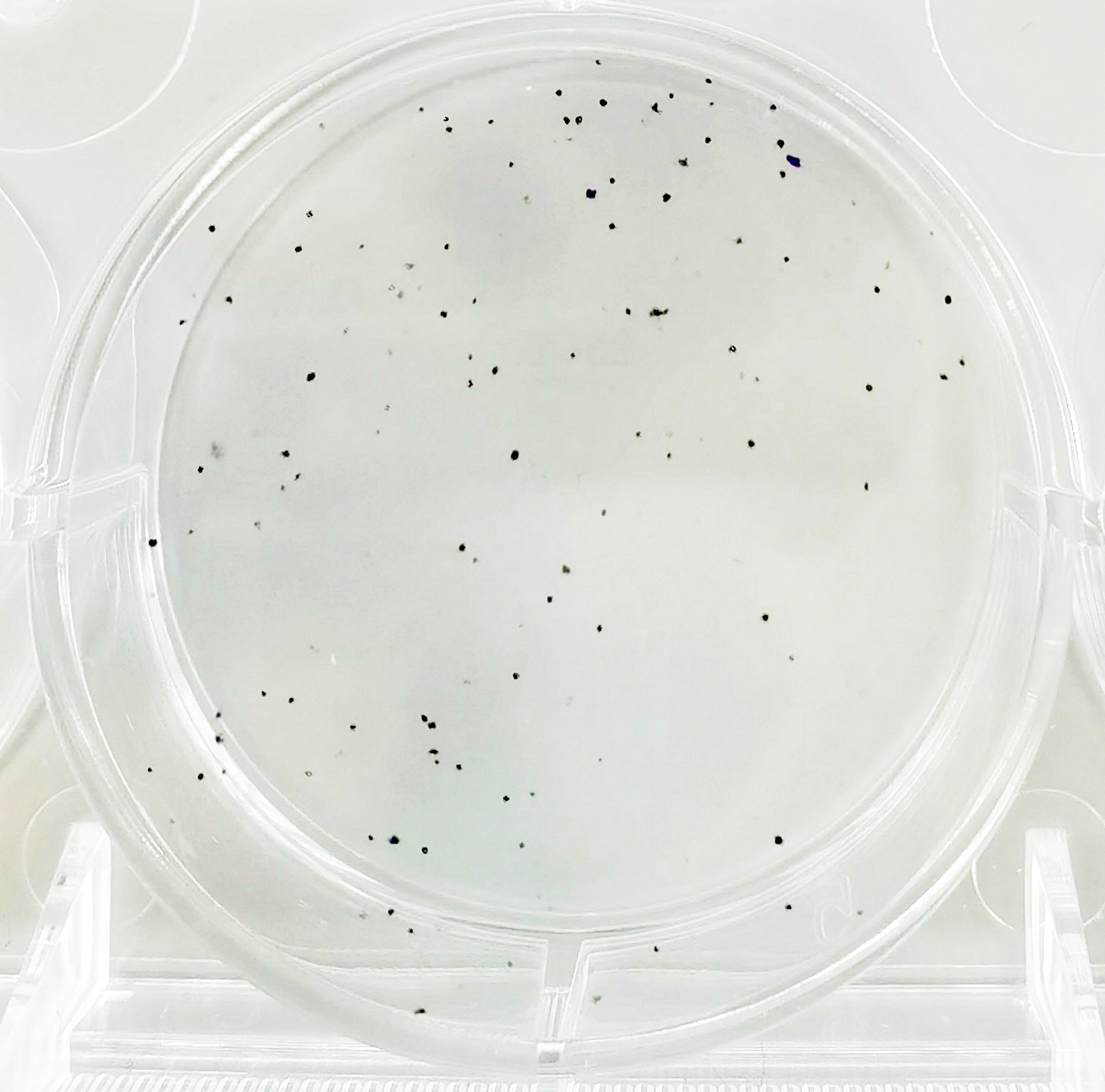

Supplement: Supplementary file 9 — Figure EV1 Source Data [file 44319_2026_749_MOESM9_ESM.zip › Figure EV1/EV 1L/25SH2-1.jpg]

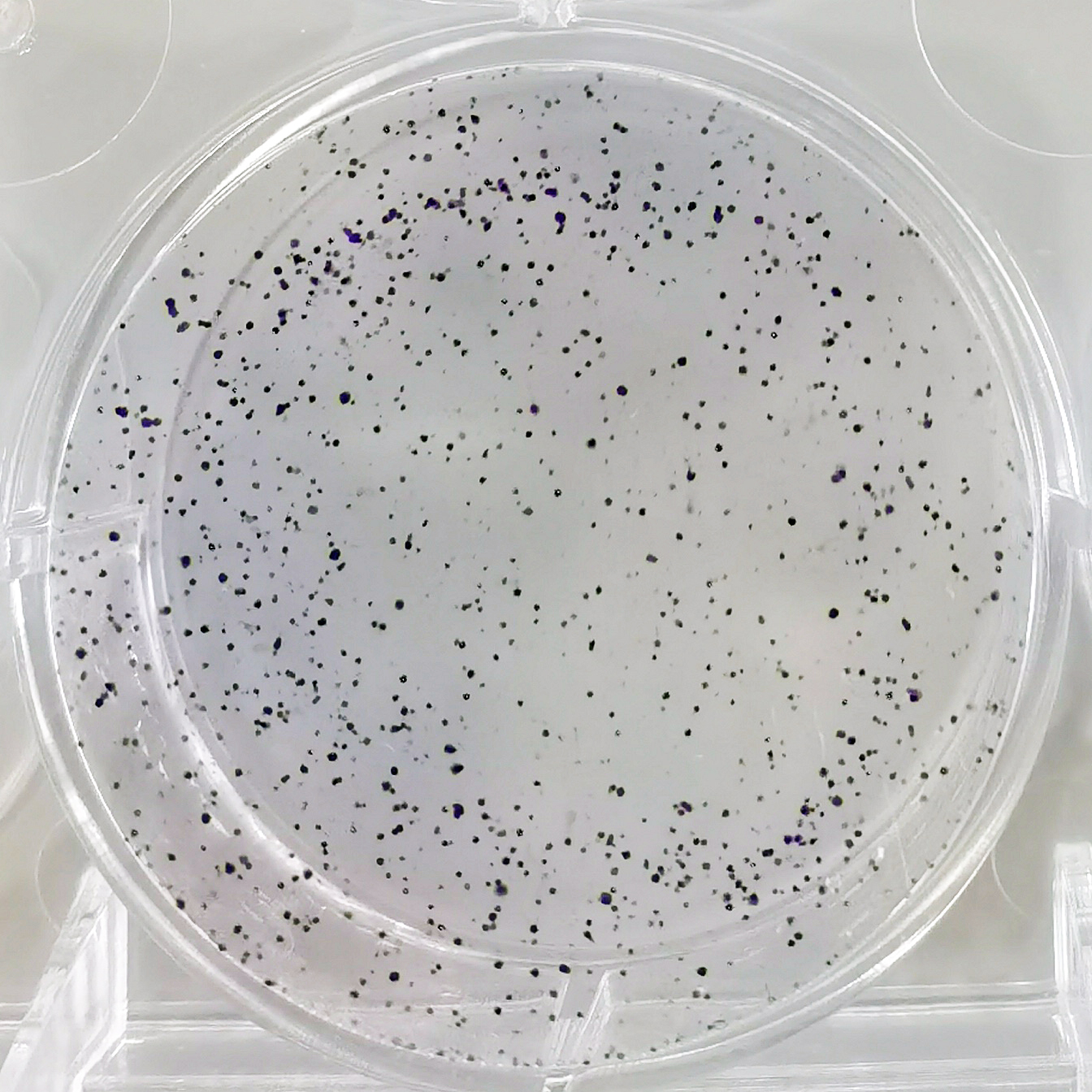

Supplement: Supplementary file 9 — Figure EV1 Source Data [file 44319_2026_749_MOESM9_ESM.zip › Figure EV1/EV 1L/CTRL.jpg]

Figure EV2A

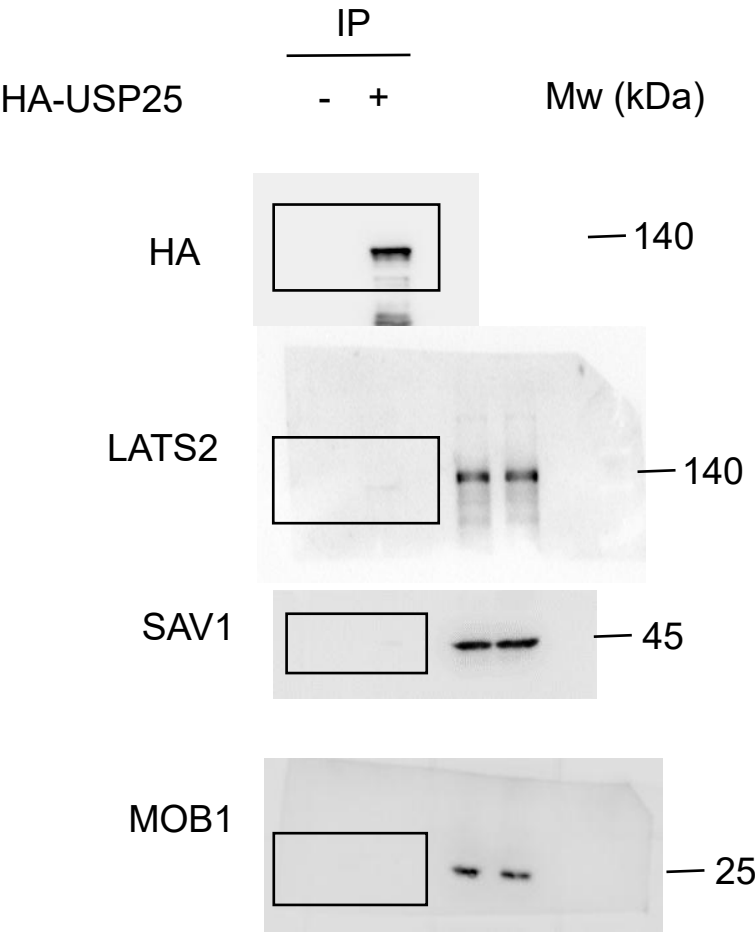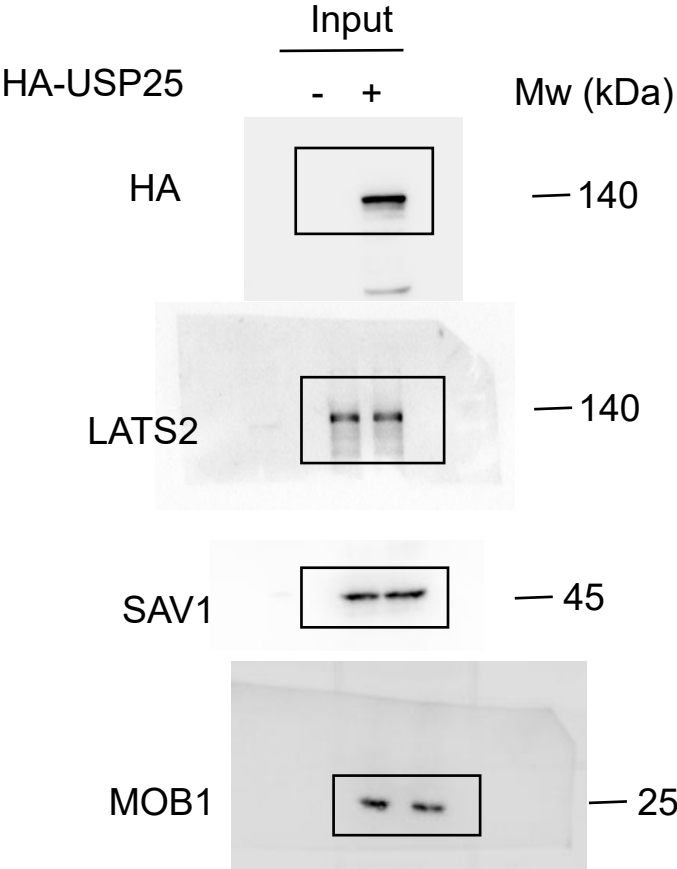

Supplement: Supplementary file 10 — Figure EV2 Source Data [file 44319_2026_749_MOESM10_ESM.zip › Figure EV2/EV 2A/Western blots EV2A.pdf]

Figure EV2B

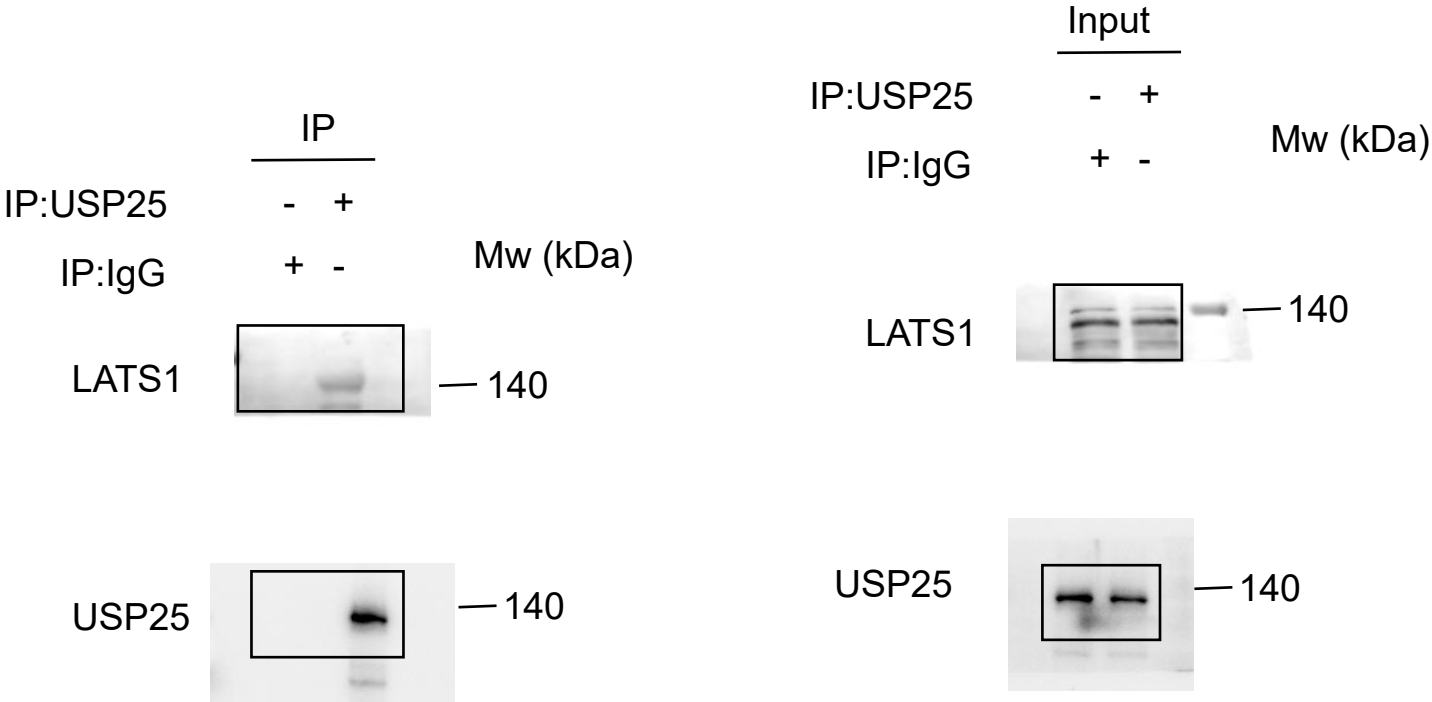

Supplement: Supplementary file 10 — Figure EV2 Source Data [file 44319_2026_749_MOESM10_ESM.zip › Figure EV2/EV 2B/Western blots EV2B.pdf]

Figure EV2C

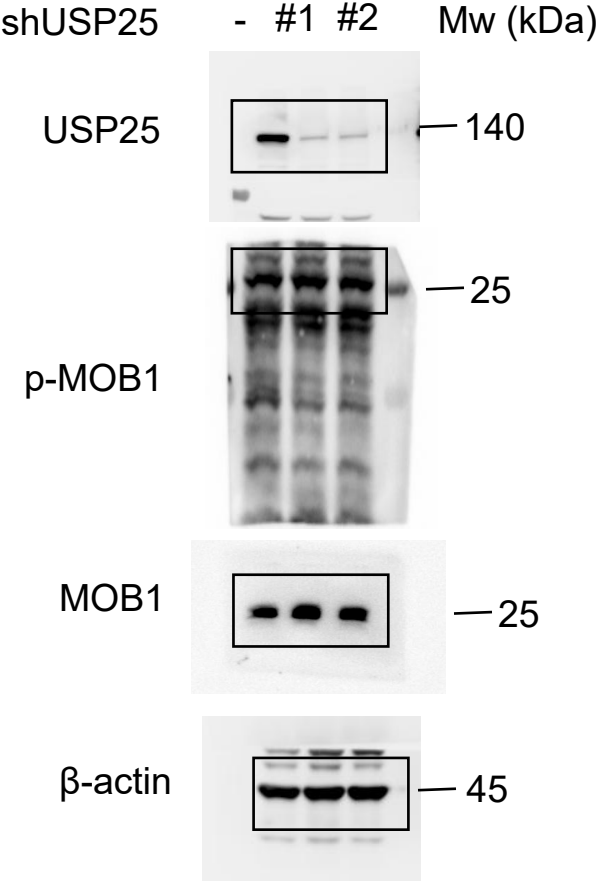

Supplement: Supplementary file 10 — Figure EV2 Source Data [file 44319_2026_749_MOESM10_ESM.zip › Figure EV2/EV 2C/Western blots EV2C.pdf]

Figure EV2D

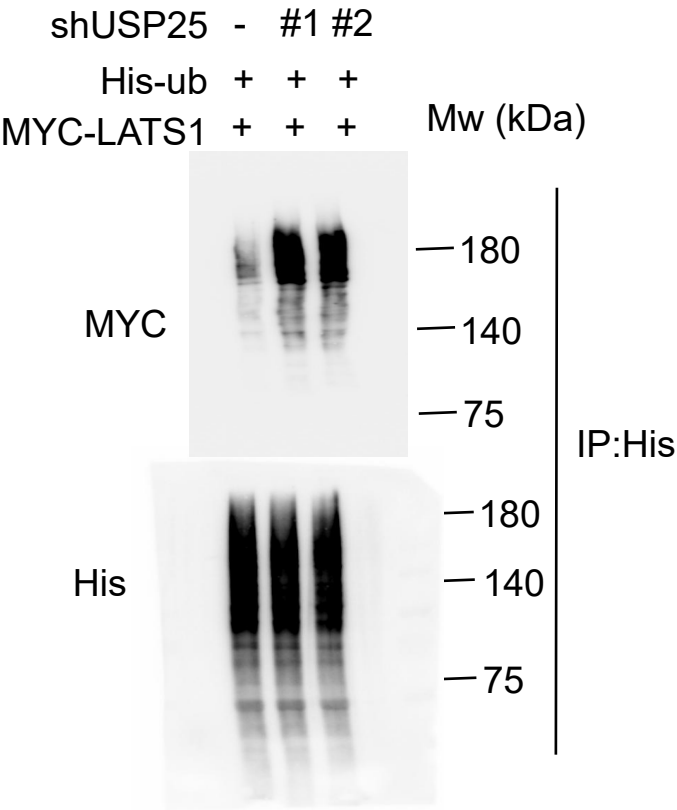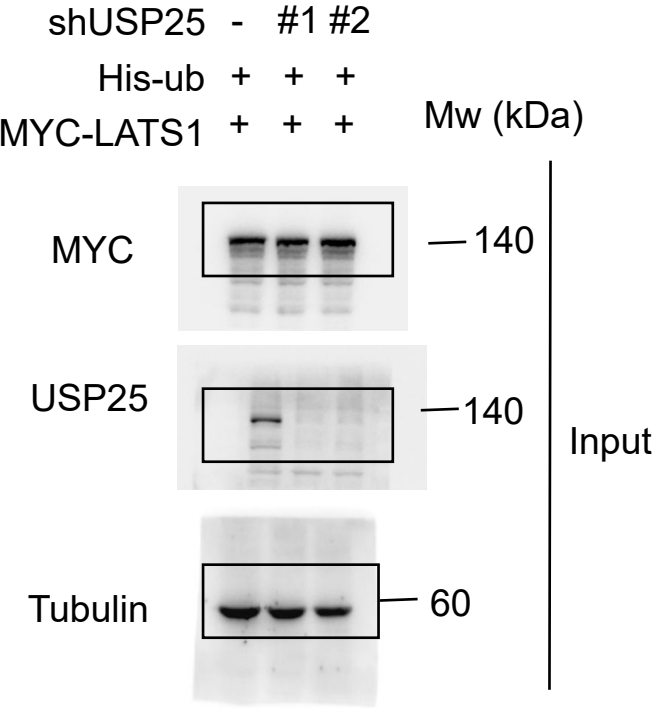

Supplement: Supplementary file 10 — Figure EV2 Source Data [file 44319_2026_749_MOESM10_ESM.zip › Figure EV2/EV 2D/Western blots EV2D.pdf]

Figure EV2E

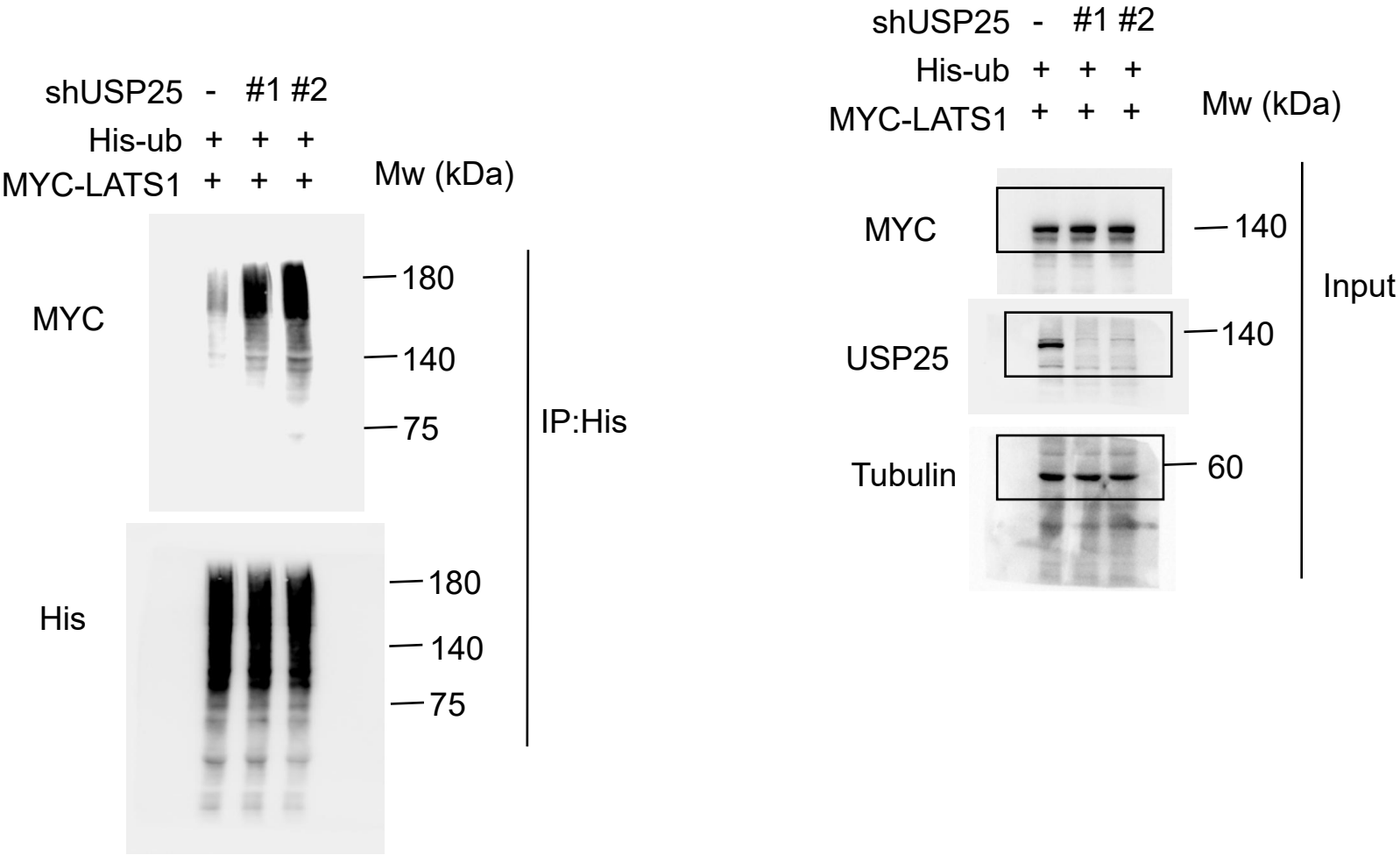

Supplement: Supplementary file 10 — Figure EV2 Source Data [file 44319_2026_749_MOESM10_ESM.zip › Figure EV2/EV 2E/Western blots EV2E.pdf]

Figure EV2G

HA-USP25 - WTCS  
His-ub + + +  
MYC-LATS1 + + +      Mw (kDa)

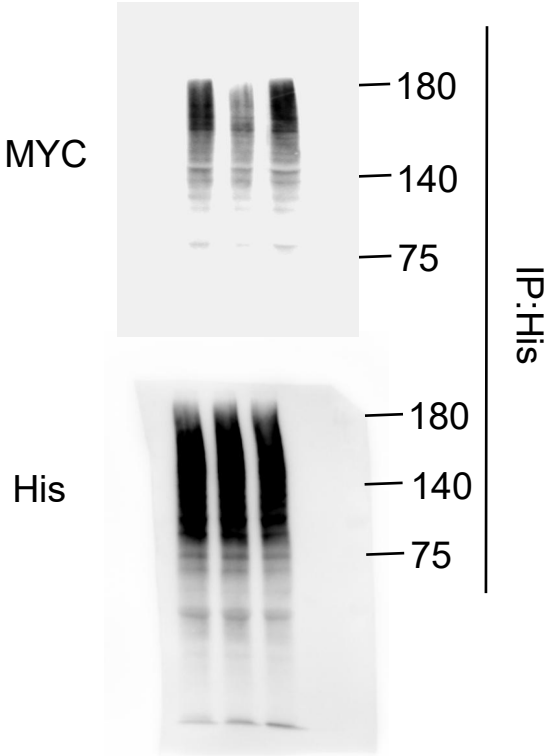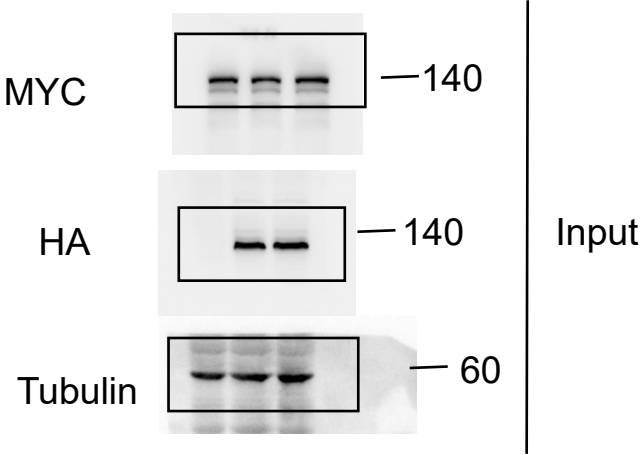

Supplement: Supplementary file 10 — Figure EV2 Source Data [file 44319_2026_749_MOESM10_ESM.zip › Figure EV2/EV 2G/Western blots EV2G.pdf]

Figure EV2H

|           |   |    |    |          |
|-----------|---|----|----|----------|
| HA-USP25  | - | WT | CS |          |
| His-ub    | + | +  | +  |          |
| MYC-LATS1 | + | +  | +  | Mw (kDa) |

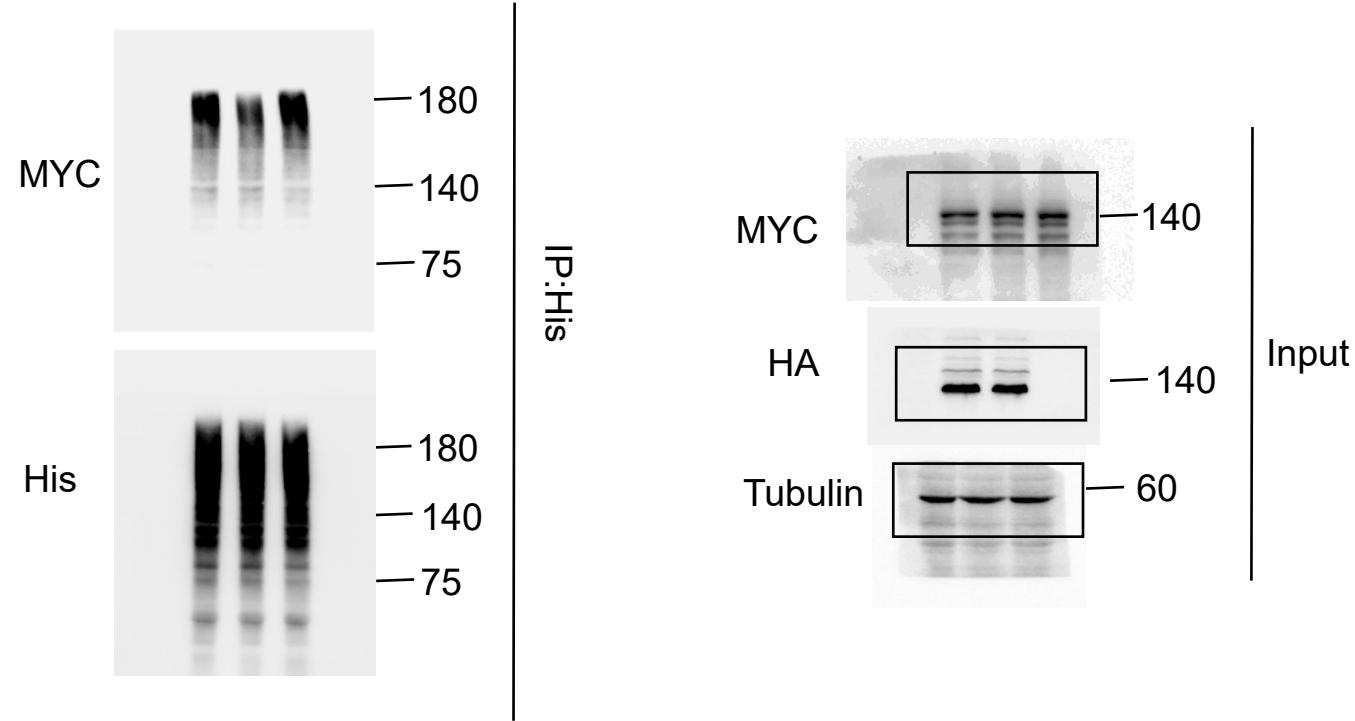

Supplement: Supplementary file 10 — Figure EV2 Source Data [file 44319_2026_749_MOESM10_ESM.zip › Figure EV2/EV 2H/Western blots EV2H.pdf]

Figure EV2J

|           |    |   |     |   |     |   |          |
|-----------|----|---|-----|---|-----|---|----------|
| His-ub    | WT |   | K48 |   | K63 |   |          |
| shUSP25   | -  | + | -   | + | -   | + |          |
| MYC-LATS1 | +  | + | +   | + | +   | + | Mw (kDa) |

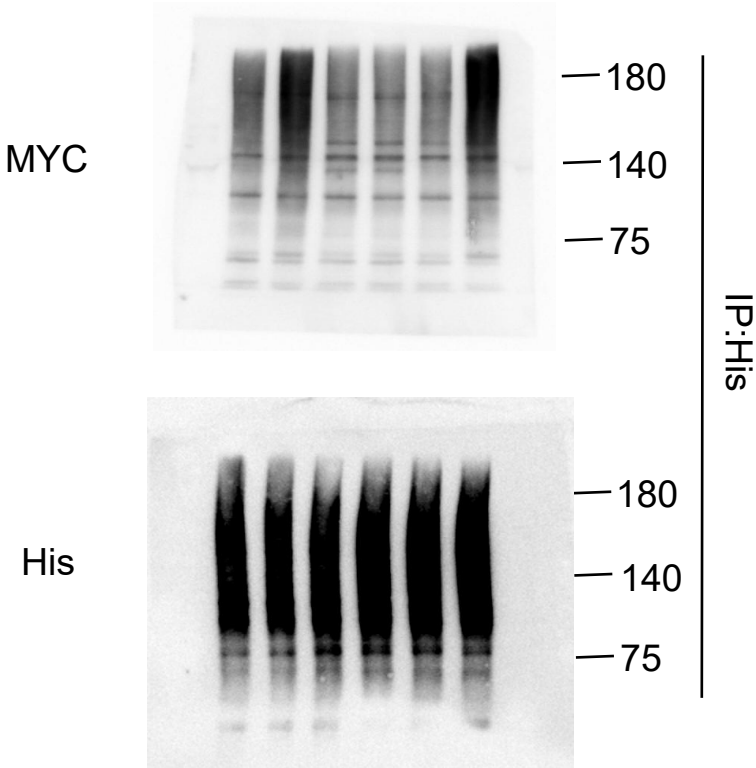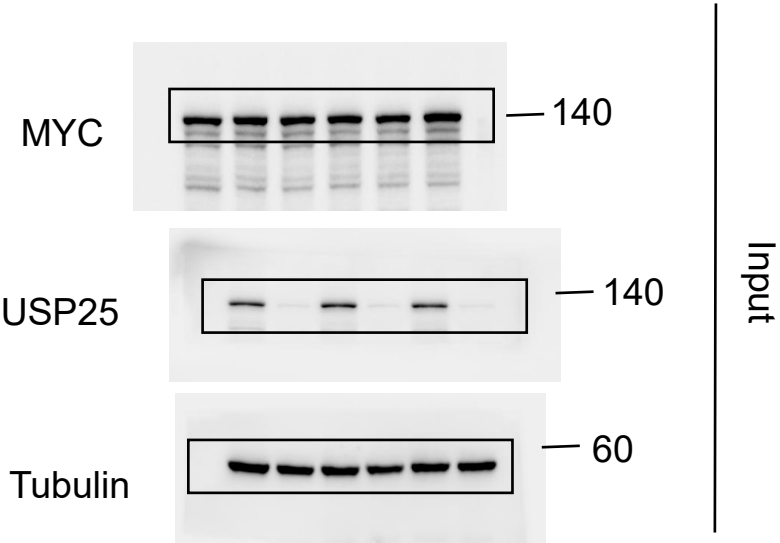

Supplement: Supplementary file 10 — Figure EV2 Source Data [file 44319_2026_749_MOESM10_ESM.zip › Figure EV2/EV 2J/Western blots EV2J.pdf]

Figure EV2K

|           |    |   |     |   |     |   |          |
|-----------|----|---|-----|---|-----|---|----------|
| His-ub    | WT |   | K48 |   | K63 |   |          |
| shUSP25   | -  | + | -   | + | -   | + |          |
| MYC-LATS1 | +  | + | +   | + | +   | + | Mw (kDa) |

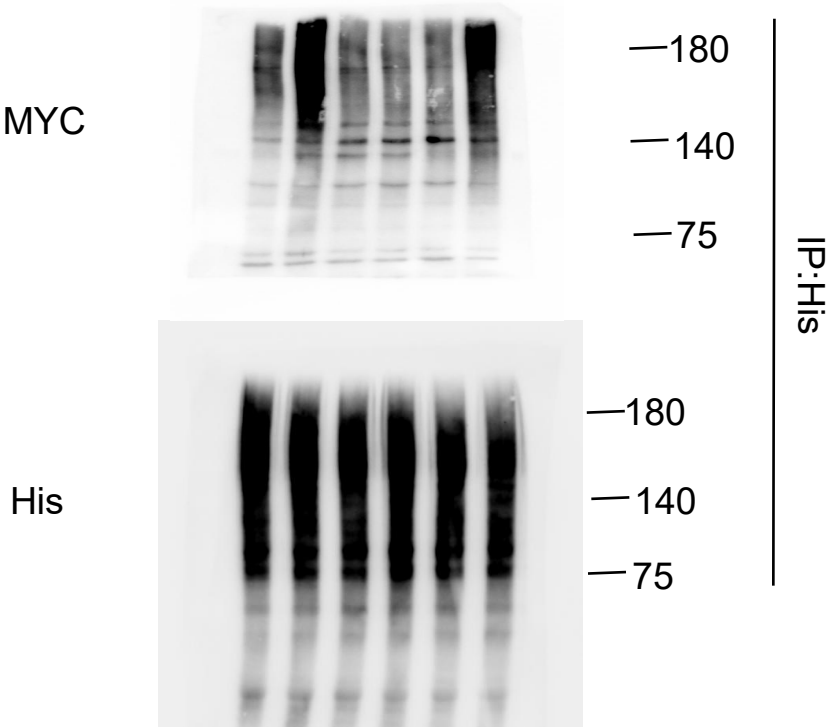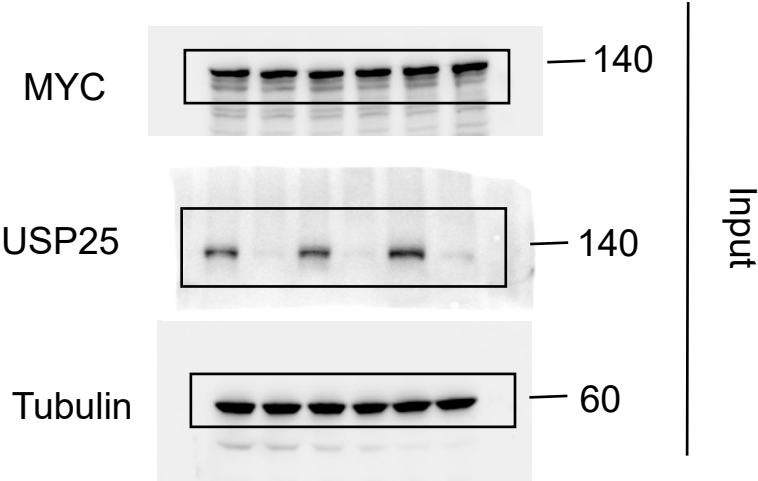

Supplement: Supplementary file 10 — Figure EV2 Source Data [file 44319_2026_749_MOESM10_ESM.zip › Figure EV2/EV 2K/Western blots EV2K.pdf]

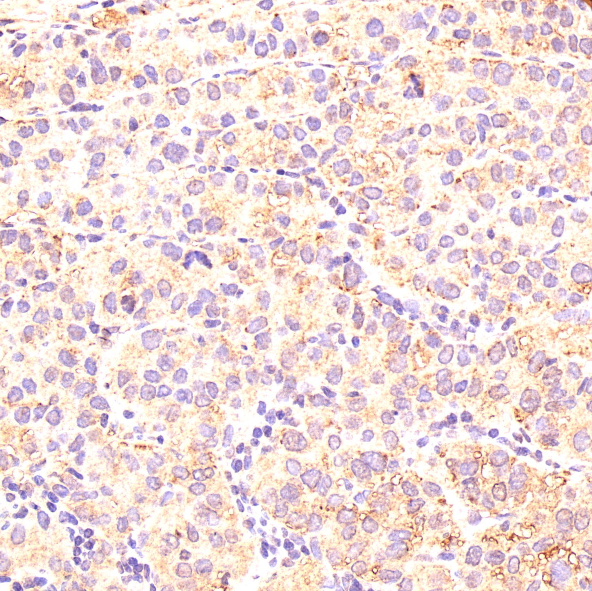

Supplement: Supplementary file 11 — Figure EV3 Source Data [file 44319_2026_749_MOESM11_ESM.zip › Figure EV3/EV 3C/USP25 ADJ.tif]

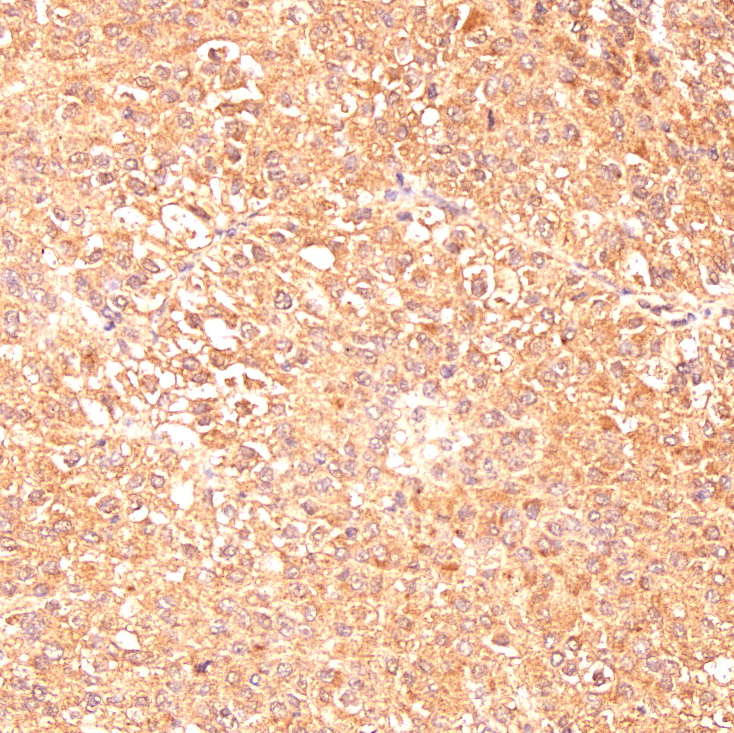

Supplement: Supplementary file 11 — Figure EV3 Source Data [file 44319_2026_749_MOESM11_ESM.zip › Figure EV3/EV 3C/USP25 HCC.tif]
