# Supplementary figures and images for: USP25 aggravates liver cancer development and impairs chemosensitivity by limiting LATS1 activation (part 2 of 2)
Source: EMBO Rep. 2026 Mar 25;27(9):2406–33. doi: 10.1038/s44319-026-00749-w (PMC13172046; doi:10.1038/s44319-026-00749-w)

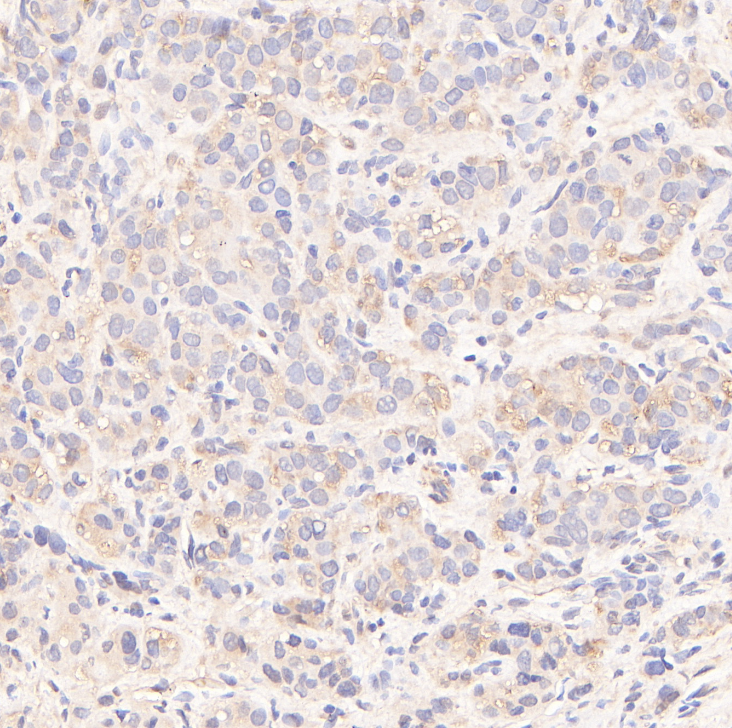

Supplement: Supplementary file 11 — Figure EV3 Source Data [file 44319_2026_749_MOESM11_ESM.zip › Figure EV3/EV 3C/YAP ADJ.tif]

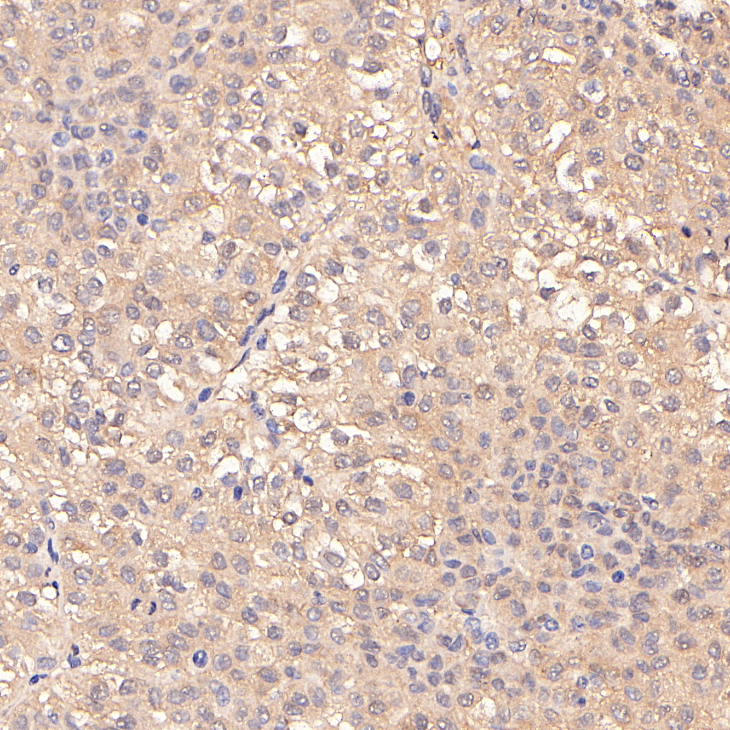

Supplement: Supplementary file 11 — Figure EV3 Source Data [file 44319_2026_749_MOESM11_ESM.zip › Figure EV3/EV 3C/YAP HCC.tif]

Figure EV3E

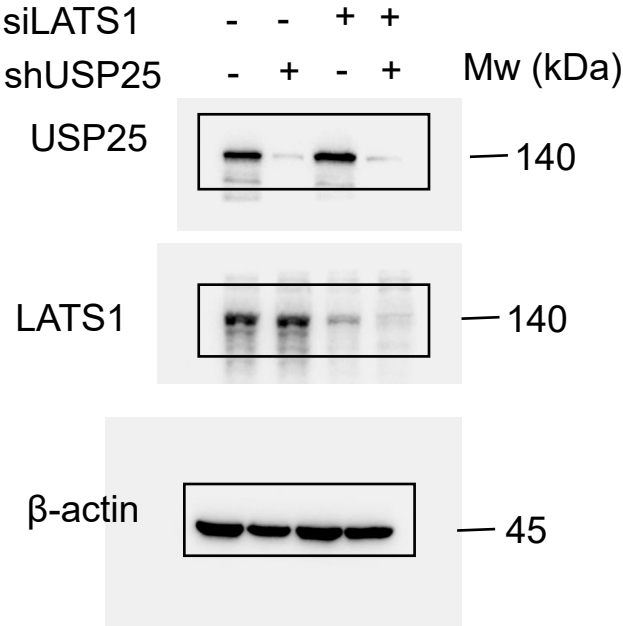

Supplement: Supplementary file 11 — Figure EV3 Source Data [file 44319_2026_749_MOESM11_ESM.zip › Figure EV3/EV 3E/Western blots EV3E.pdf]

Figure EV3I

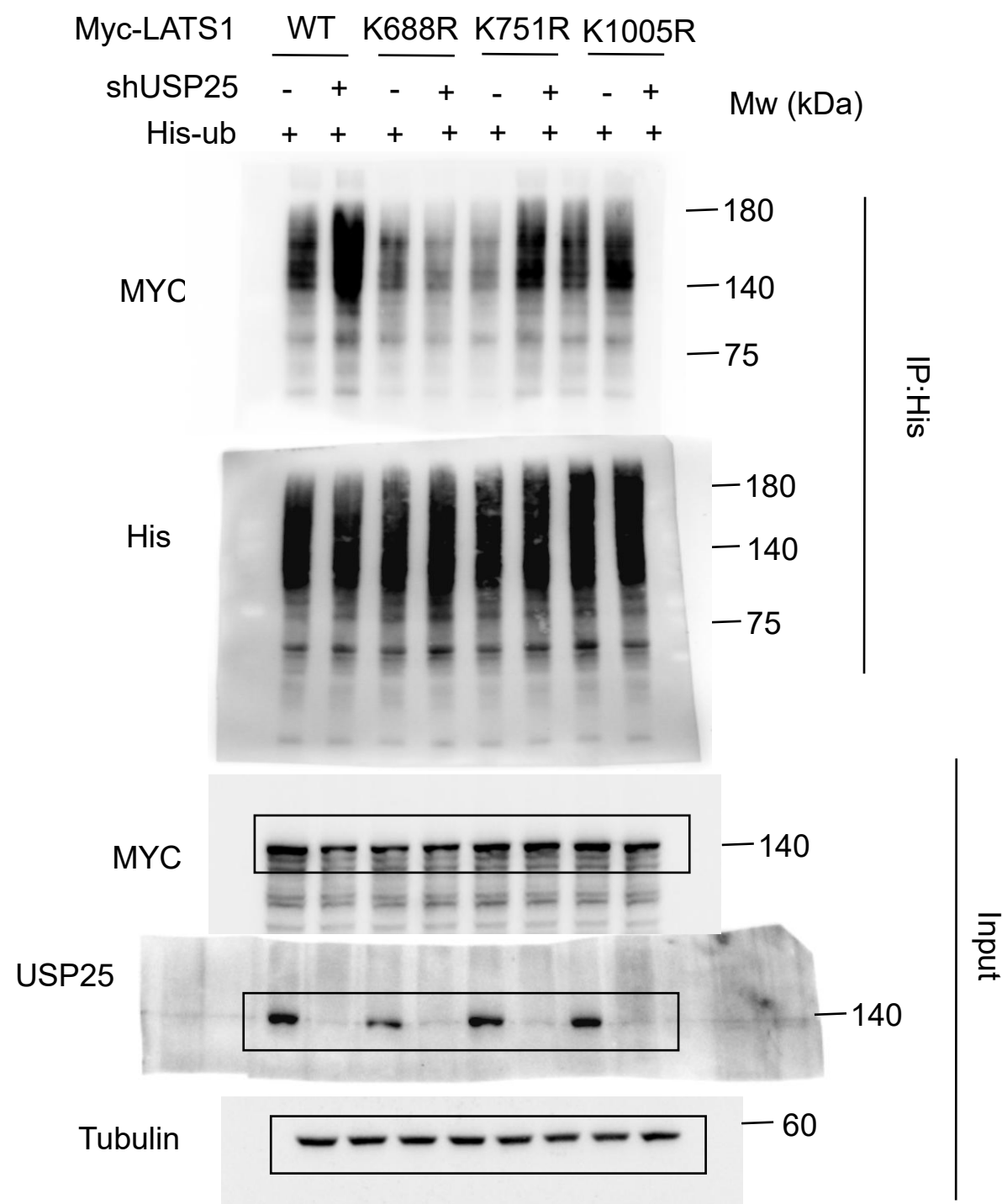

Supplement: Supplementary file 11 — Figure EV3 Source Data [file 44319_2026_749_MOESM11_ESM.zip › Figure EV3/EV 3I/Western blots EV3I.pdf]

Figure EV3J

|           |    |   |       |   |       |   |        |   |          |
|-----------|----|---|-------|---|-------|---|--------|---|----------|
| Myc-LAST1 | WT |   | K688R |   | K751R |   | K1005R |   |          |
| shUSP25   | -  | + | -     | + | -     | + | -      | + |          |
| His-ub    | +  | + | +     | + | +     | + | +      | + | Mw (kDa) |

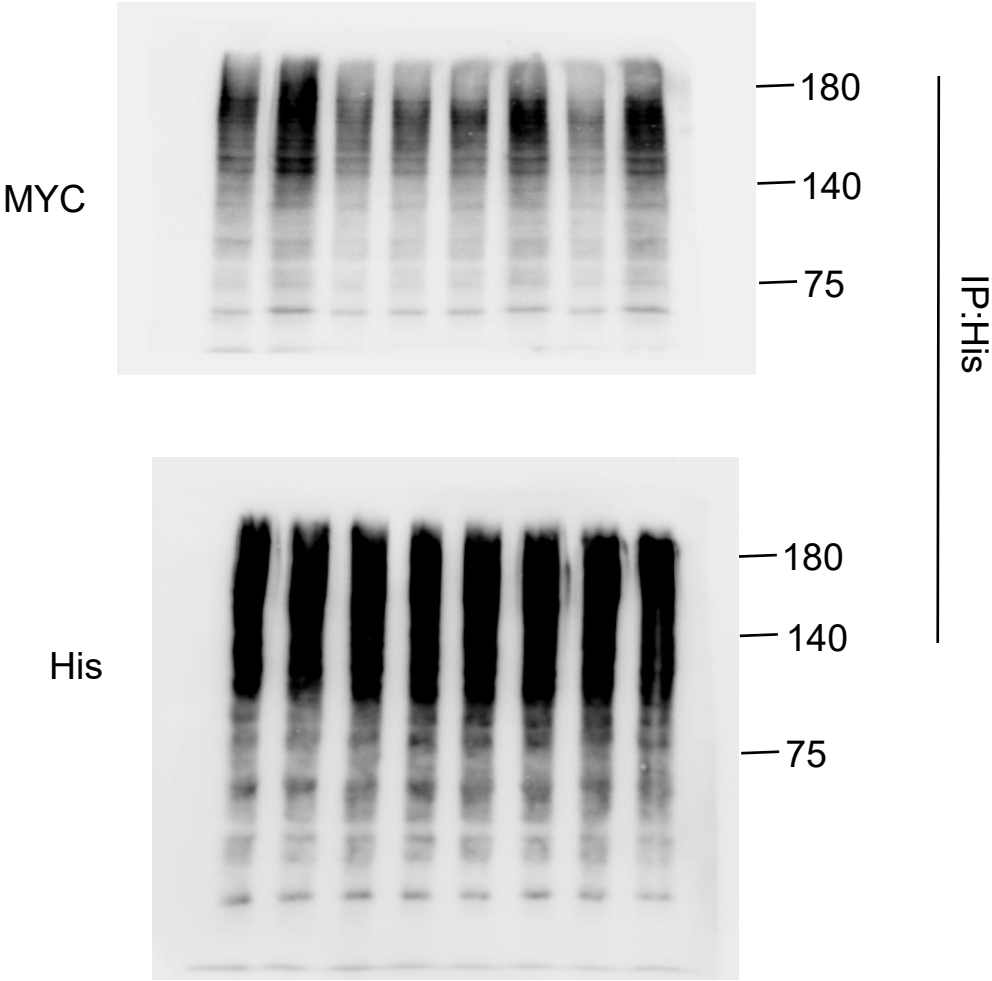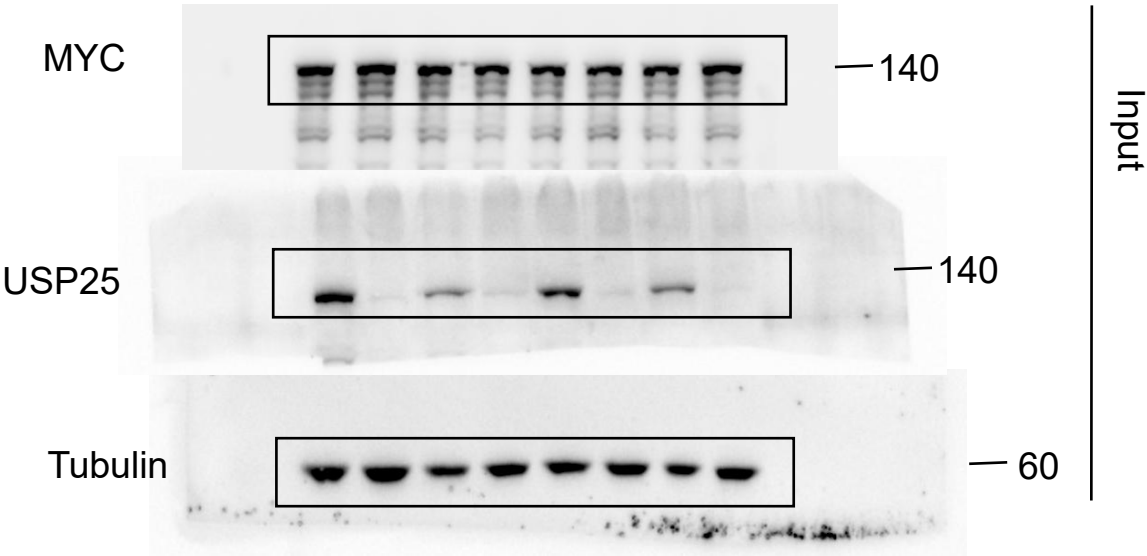

Supplement: Supplementary file 11 — Figure EV3 Source Data [file 44319_2026_749_MOESM11_ESM.zip › Figure EV3/EV 3J/Western blots EV3J.pdf]

Figure EV3L

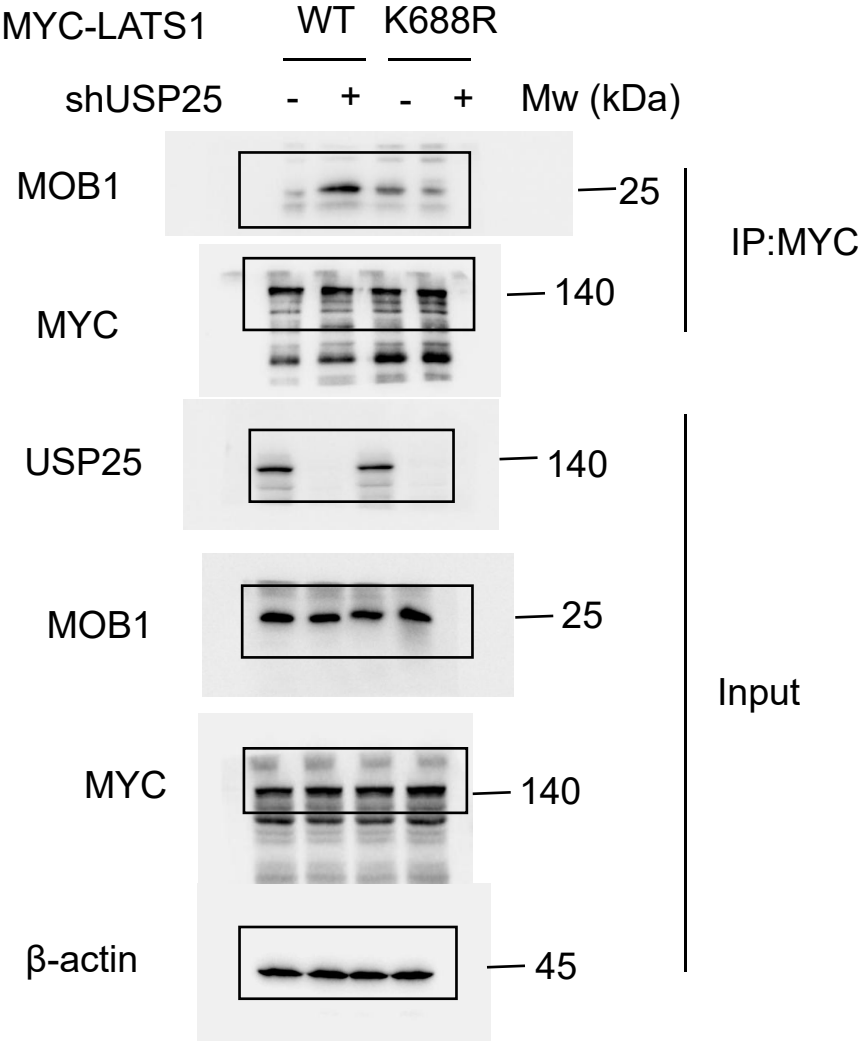

Supplement: Supplementary file 11 — Figure EV3 Source Data [file 44319_2026_749_MOESM11_ESM.zip › Figure EV3/EV 3L/Western blots EV3L.pdf]

Figure EV3M

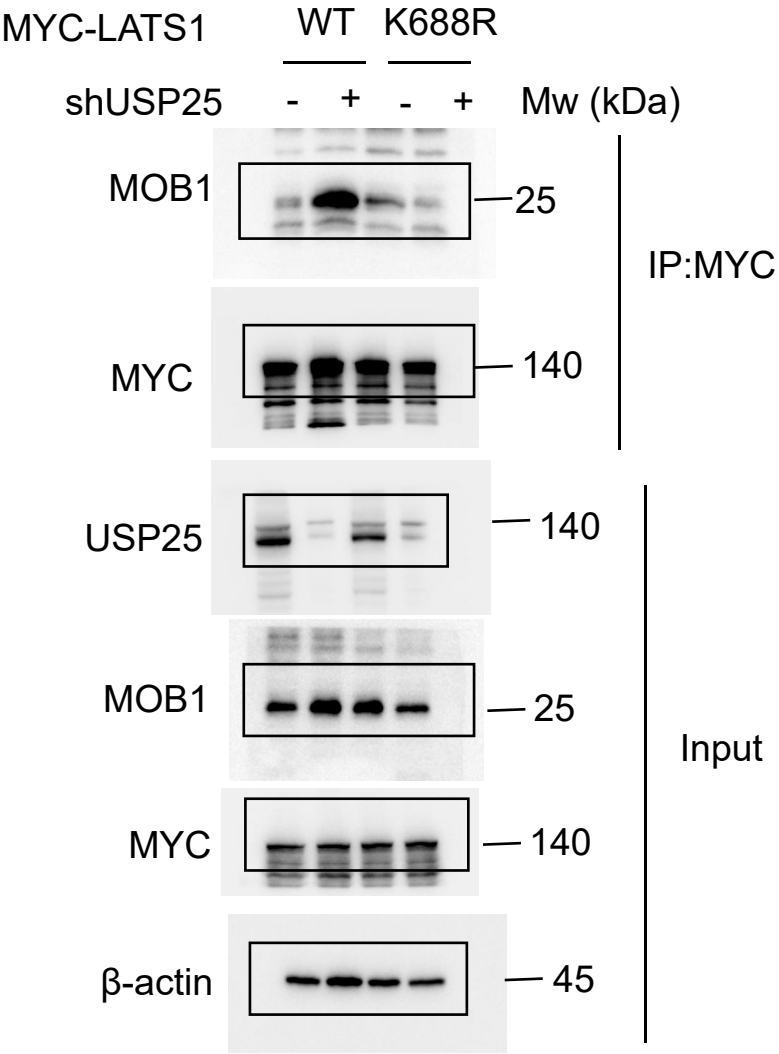

Supplement: Supplementary file 11 — Figure EV3 Source Data [file 44319_2026_749_MOESM11_ESM.zip › Figure EV3/EV 3M/Western blots EV3M.pdf]

Figure EV4F

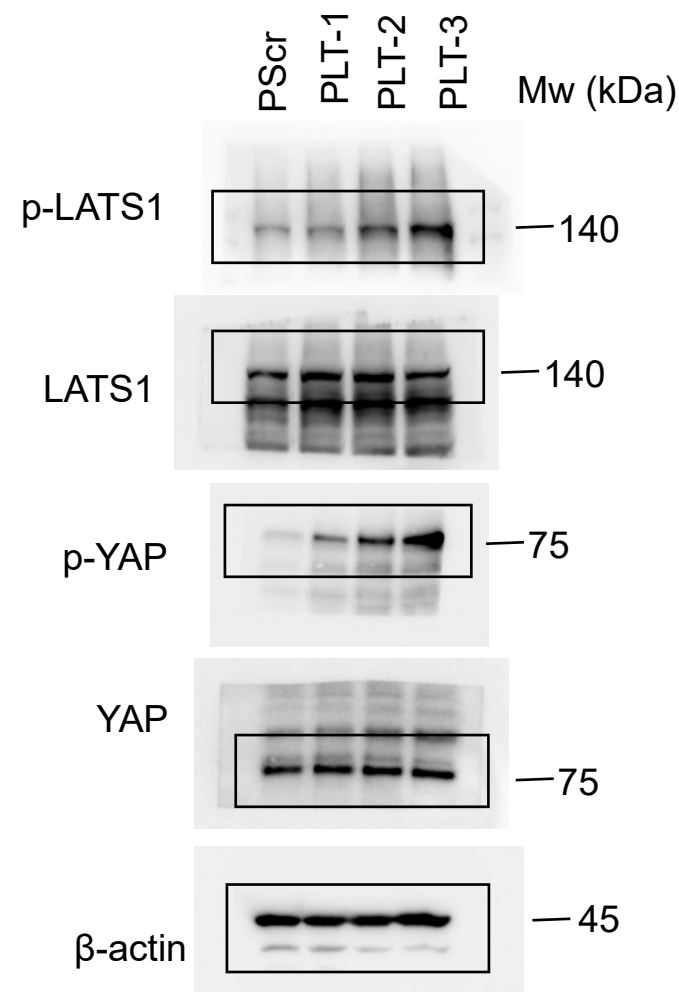

Supplement: Supplementary file 12 — Figure EV4 Source Data [file 44319_2026_749_MOESM12_ESM.zip › Figure EV4/EV 4F/Western blots EV4F.pdf]

Figure EV4G

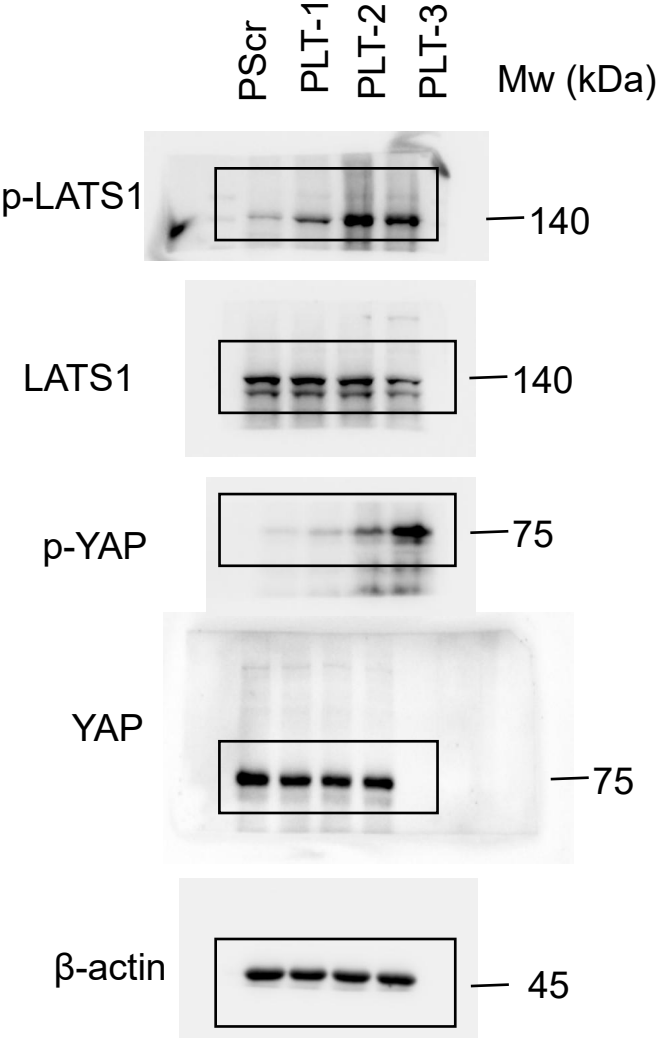

Supplement: Supplementary file 12 — Figure EV4 Source Data [file 44319_2026_749_MOESM12_ESM.zip › Figure EV4/EV 4G/Western blots EV4G.pdf]

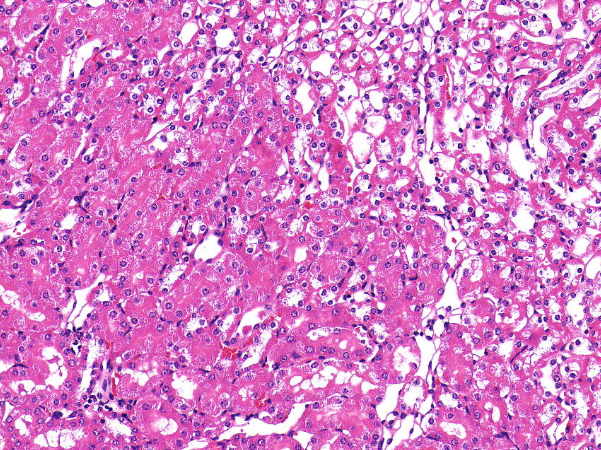

Supplement: Supplementary file 13 — Figure EV5 Source Data [file 44319_2026_749_MOESM13_ESM.zip › Figure EV5/EV 5F/Peptide kidney.tif]

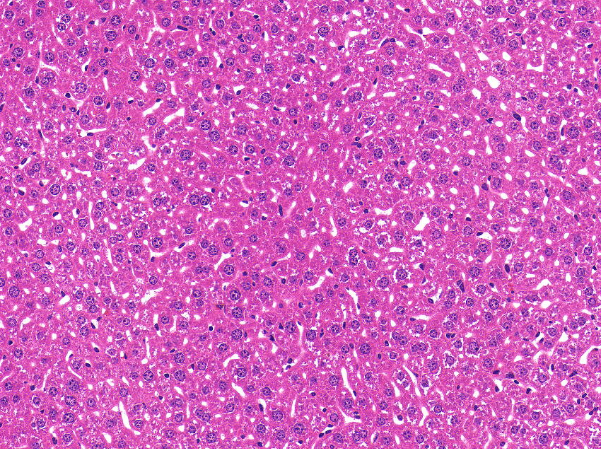

Supplement: Supplementary file 13 — Figure EV5 Source Data [file 44319_2026_749_MOESM13_ESM.zip › Figure EV5/EV 5F/Peptide liver.tif]

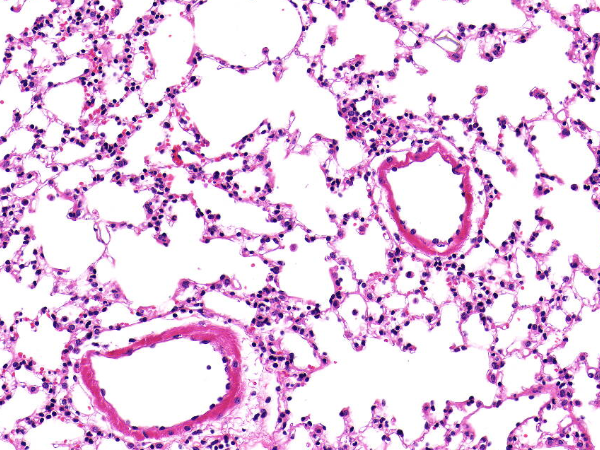

Supplement: Supplementary file 13 — Figure EV5 Source Data [file 44319_2026_749_MOESM13_ESM.zip › Figure EV5/EV 5F/Peptide lung.tif]

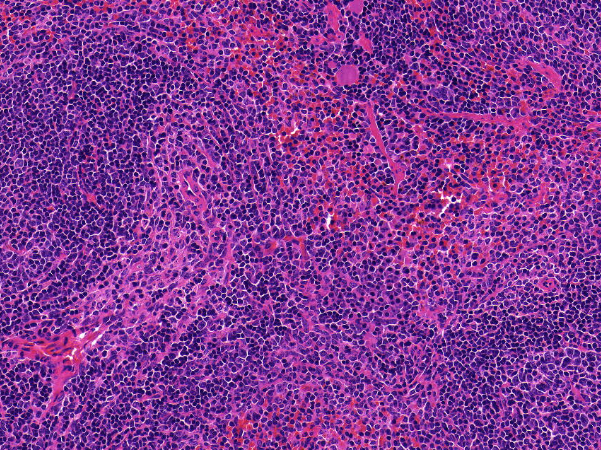

Supplement: Supplementary file 13 — Figure EV5 Source Data [file 44319_2026_749_MOESM13_ESM.zip › Figure EV5/EV 5F/Peptide spleen.tif]

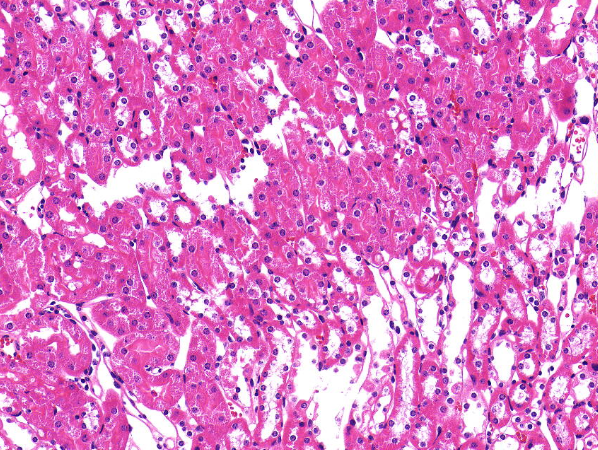

Supplement: Supplementary file 13 — Figure EV5 Source Data [file 44319_2026_749_MOESM13_ESM.zip › Figure EV5/EV 5F/peptide+Sorafenib kidney.tif]

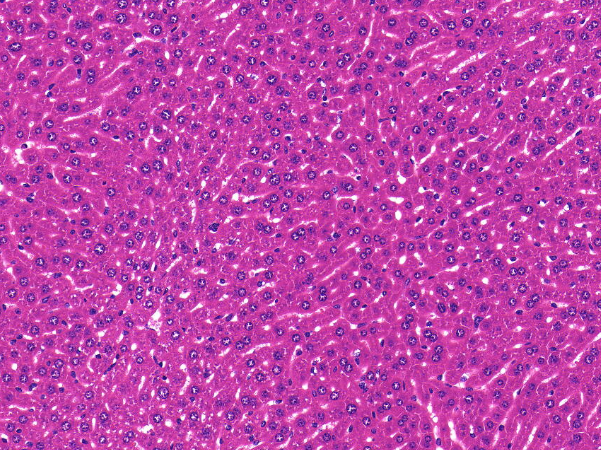

Supplement: Supplementary file 13 — Figure EV5 Source Data [file 44319_2026_749_MOESM13_ESM.zip › Figure EV5/EV 5F/peptide+Sorafenib liver.tif]

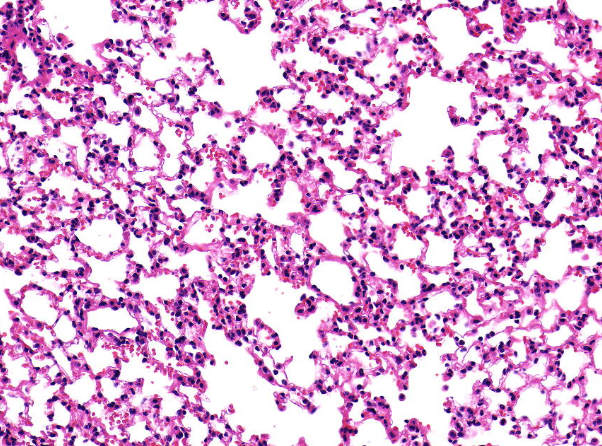

Supplement: Supplementary file 13 — Figure EV5 Source Data [file 44319_2026_749_MOESM13_ESM.zip › Figure EV5/EV 5F/peptide+Sorafenib lung.tif]

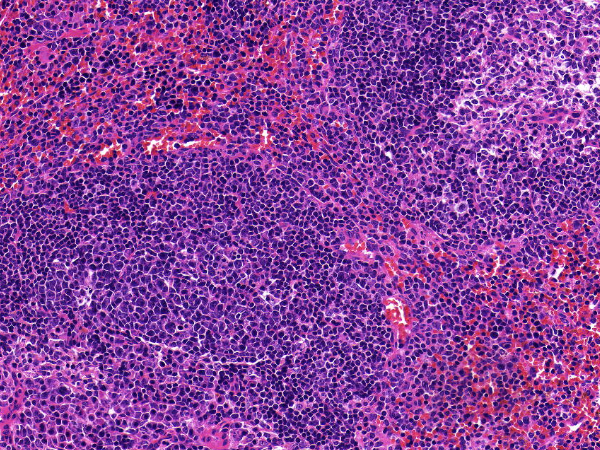

Supplement: Supplementary file 13 — Figure EV5 Source Data [file 44319_2026_749_MOESM13_ESM.zip › Figure EV5/EV 5F/peptide+Sorafenib spleen.tif]

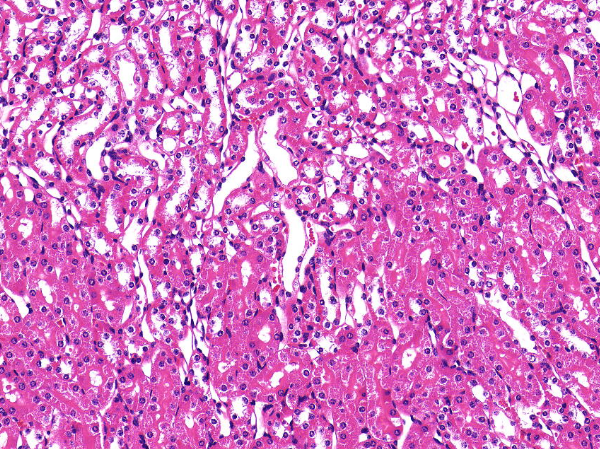

Supplement: Supplementary file 13 — Figure EV5 Source Data [file 44319_2026_749_MOESM13_ESM.zip › Figure EV5/EV 5F/PScr kidney.tif]

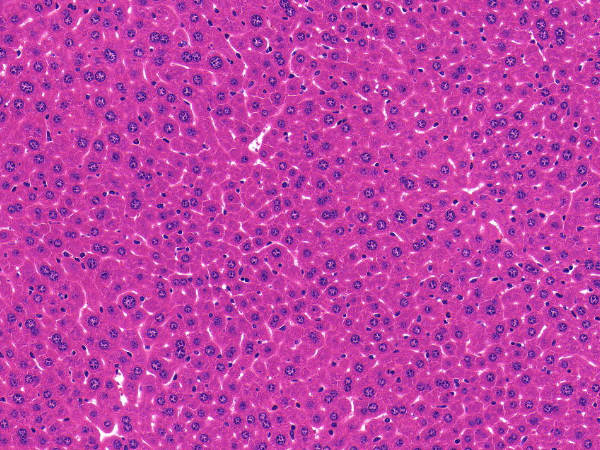

Supplement: Supplementary file 13 — Figure EV5 Source Data [file 44319_2026_749_MOESM13_ESM.zip › Figure EV5/EV 5F/PScr liver.tif]

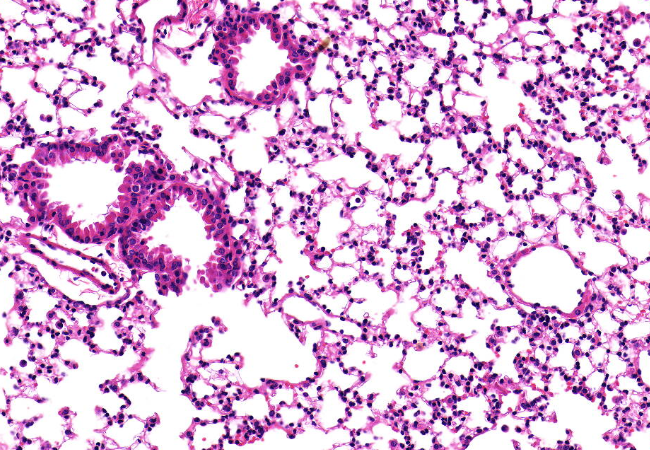

Supplement: Supplementary file 13 — Figure EV5 Source Data [file 44319_2026_749_MOESM13_ESM.zip › Figure EV5/EV 5F/PScr lung.tif]

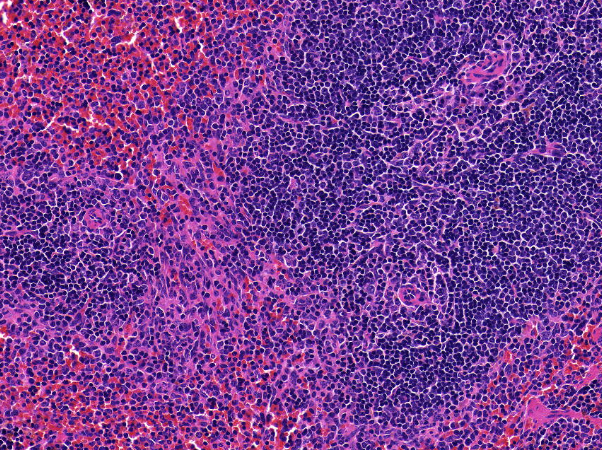

Supplement: Supplementary file 13 — Figure EV5 Source Data [file 44319_2026_749_MOESM13_ESM.zip › Figure EV5/EV 5F/PScr spleen.tif]

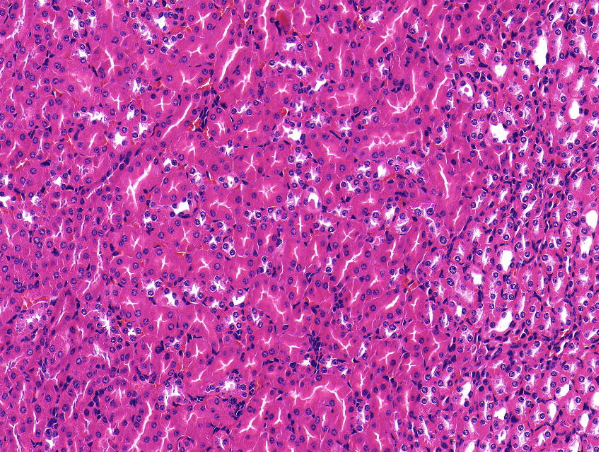

Supplement: Supplementary file 13 — Figure EV5 Source Data [file 44319_2026_749_MOESM13_ESM.zip › Figure EV5/EV 5F/Sorafenib kidney.tif]

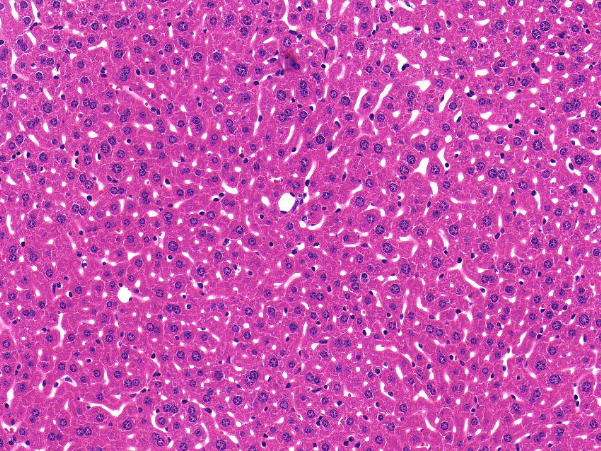

Supplement: Supplementary file 13 — Figure EV5 Source Data [file 44319_2026_749_MOESM13_ESM.zip › Figure EV5/EV 5F/Sorafenib liver.tif]

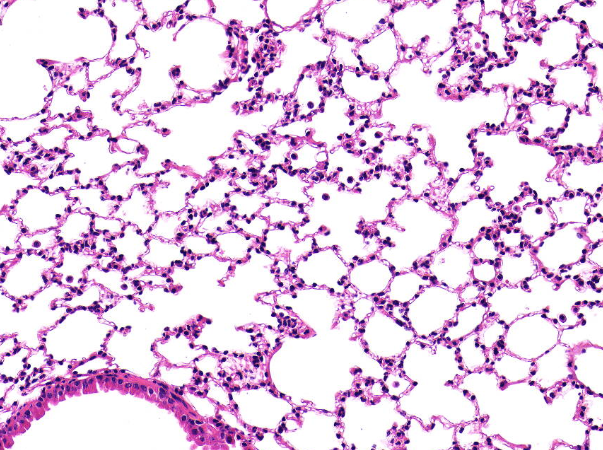

Supplement: Supplementary file 13 — Figure EV5 Source Data [file 44319_2026_749_MOESM13_ESM.zip › Figure EV5/EV 5F/Sorafenib lung.tif]

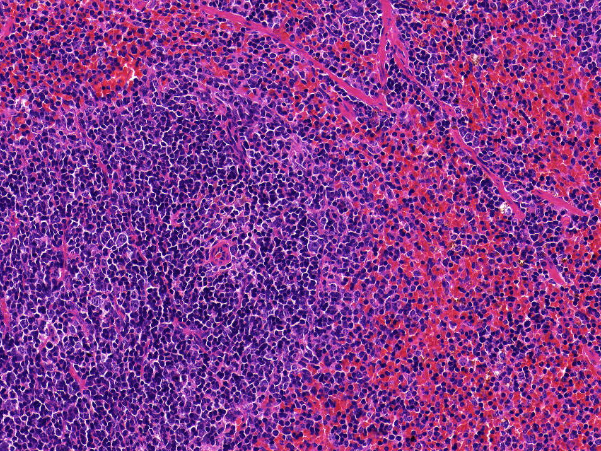

Supplement: Supplementary file 13 — Figure EV5 Source Data [file 44319_2026_749_MOESM13_ESM.zip › Figure EV5/EV 5F/Sorafenib spleen.tif]
