# Supplementary material for: USP25 aggravates liver cancer development and impairs chemosensitivity by limiting LATS1 activation
Source: EMBO Rep. 2026 Mar 25;27(9):2406–33. doi: 10.1038/s44319-026-00749-w (PMC13172046; doi:10.1038/s44319-026-00749-w)
Supplement: Supplementary file 14 — Expanded View Figures [file 44319_2026_749_MOESM14_ESM.pdf]

Expanded View Figures

Figure EV1. USP25 protein levels are high in various organs, and USP25 promotes HCC progression in vitro.

(**A**, **B**) USP25 protein levels in the heart, liver, kidney, WAT, BAT, epididymis and muscle were measured by western blotting and quantified by densitometric analysis with ImageJ. The quantification results are presented in Fig. 1G. (**C**, **D**) Blood biochemistry results are shown for 2-month-old mice (**C**) and 4-month-old mice (**D**). Representative data (mean ± SD) are shown from 7 biologically independent animals. ALT (2 M), *Usp25*$^{+/+}$ vs. *Usp25*$^{-/-}$ ($P = 0.9617$), AST (2 M), *Usp25*$^{+/+}$ vs. *Usp25*$^{-/-}$ ($P = 0.7125$), TC (2 M), *Usp25*$^{+/+}$ vs. *Usp25*$^{-/-}$ ($P = 0.8708$), TG (2 M), *Usp25*$^{+/+}$ vs. *Usp25*$^{-/-}$ ($P = 0.1748$), HDL-C (2 M), *Usp25*$^{+/+}$ vs. *Usp25*$^{-/-}$ ($P = 0.6143$), LDL-C (2 M), *Usp25*$^{+/+}$ vs. *Usp25*$^{-/-}$ ($P = 0.2528$), NEFA (2 M), and *Usp25*$^{+/+}$ vs. *Usp25*$^{-/-}$ ($P = 0.4035$). ALT (4 M), *Usp25*$^{+/+}$ vs. *Usp25*$^{-/-}$ ($P = 0.6054$), AST (4 M), *Usp25*$^{+/+}$ vs. *Usp25*$^{-/-}$ ($P = 0.666$), TC (4 M), *Usp25*$^{+/+}$ vs. *Usp25*$^{-/-}$ ($P = 0.3391$), TG (4 M), *Usp25*$^{+/+}$ vs. *Usp25*$^{-/-}$ ($P = 0.8997$), HDL-C (4 M), *Usp25*$^{+/+}$ vs. *Usp25*$^{-/-}$ ($P = 0.8071$), LDL-C (4 M), *Usp25*$^{+/+}$ vs. *Usp25*$^{-/-}$ ($P = 0.5813$), NEFA (4 M), and *Usp25*$^{+/+}$ vs. *Usp25*$^{-/-}$ ($P = 0.8623$). (**E–G**) The protein level of USP25 in liver cancer cell lines and normal liver cell lines was determined by western blot. USP25 levels were quantified by densitometric analysis with ImageJ. The quantification results are presented ($n = 3$) (**G**). LO2 vs. HepG2 ($P < 0.0001$), LO2 vs. 7721 ($P < 0.0001$), LO2 vs. Hep3B ($P < 0.0001$), and LO2 vs. Huh7 ($P = 0.9608$). (**H**) USP25 knockdown was achieved in 7721 cells by transfection with lentivirus containing specific short hairpin RNA (control, #1 sh-USP25 or #2 sh-USP25) and confirmed by western blot. (**I**) 7721 cells were infected with USP25 lentivirus and subjected to western blot. (**J**, **K**) The proliferation of USP25-knockdown (**J**) or USP25-overexpressing (**K**) 7721 cells was quantified via a CCK-8 assay. Representative data (mean ± SD) are shown from 3 biologically independent samples. Ctrl vs. shUSP25#1 ($P < 0.0001$), Ctrl vs. shUSP25#2 ($P < 0.0001$), and Ctrl vs. USP25^{WT} ($P < 0.0001$). (**L**, **M**) Colony formation assays of 7721 cells after USP25 knockdown. Representative data (mean ± SD) are shown from 3 biologically independent samples. Ctrl vs. shUSP25#1 ($P < 0.0001$) and Ctrl vs. shUSP25#2 ($P < 0.0001$). Statistical analysis was performed via *t* tests (**C**, **D**), one-way ANOVA (**G**, **M**) or two-way ANOVA (**J**, **K**) followed by Tukey's multiple comparison test. Source data are available online for this figure.

 © The Author(s)

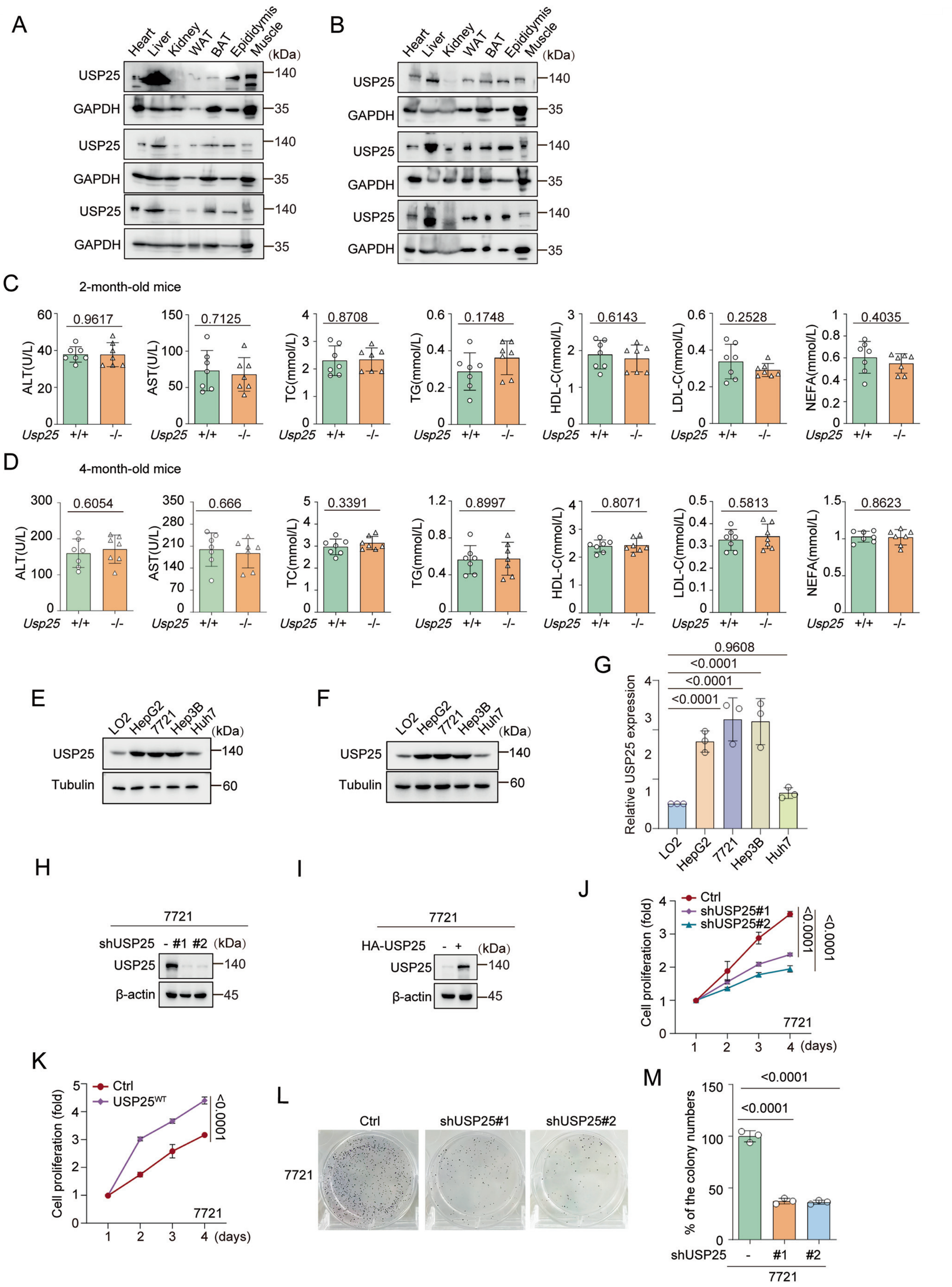
A
Heart Liver Kidney WAT BAT Epididymis Muscle
(kDa)
USP25 140
GAPDH 35
B
Heart Liver Kidney WAT BAT Epididymis Muscle
(kDa)
USP25 140
GAPDH 35
C
2-month-old mice
ALT(U/L) 0.9617
AST(U/L) 0.7125
TC(mmol/L) 0.8708
TG(mmol/L) 0.1748
HDL-C(mmol/L) 0.6143
LDL-C(mmol/L) 0.2528
NEFA(mmol/L) 0.4035
Usp25 +/+ -/-
D
4-month-old mice
ALT(U/L) 0.6054
AST(U/L) 0.666
TC(mmol/L) 0.3391
TG(mmol/L) 0.8997
HDL-C(mmol/L) 0.8071
LDL-C(mmol/L) 0.5813
NEFA(mmol/L) 0.8623
Usp25 +/+ -/-
E
LO2 HepG2 7721 Hep3B Huh7
(kDa)
USP25 140
Tubulin 60
F
LO2 HepG2 7721 Hep3B Huh7
(kDa)
USP25 140
Tubulin 60
G
Relative USP25 expression
0.9608
<0.0001
<0.0001
<0.0001
LO2 HepG2 7721 Hep3B Huh7
H
7721
shUSP25 - #1 #2 (kDa)
USP25 140
β-actin 45
I
7721
HA-USP25 - + (kDa)
USP25 140
β-actin 45
J
Ctrl
shUSP25#1
shUSP25#2
Cell proliferation (fold)
<0.0001
<0.0001
7721
1 2 3 4 (days)
K
Ctrl
USP25WT
Cell proliferation (fold)
<0.0001
7721
1 2 3 4 (days)
L
Ctrl shUSP25#1 shUSP25#2
7721
M
% of the colony numbers
<0.0001
<0.0001
shUSP25 - #1 #2
7721

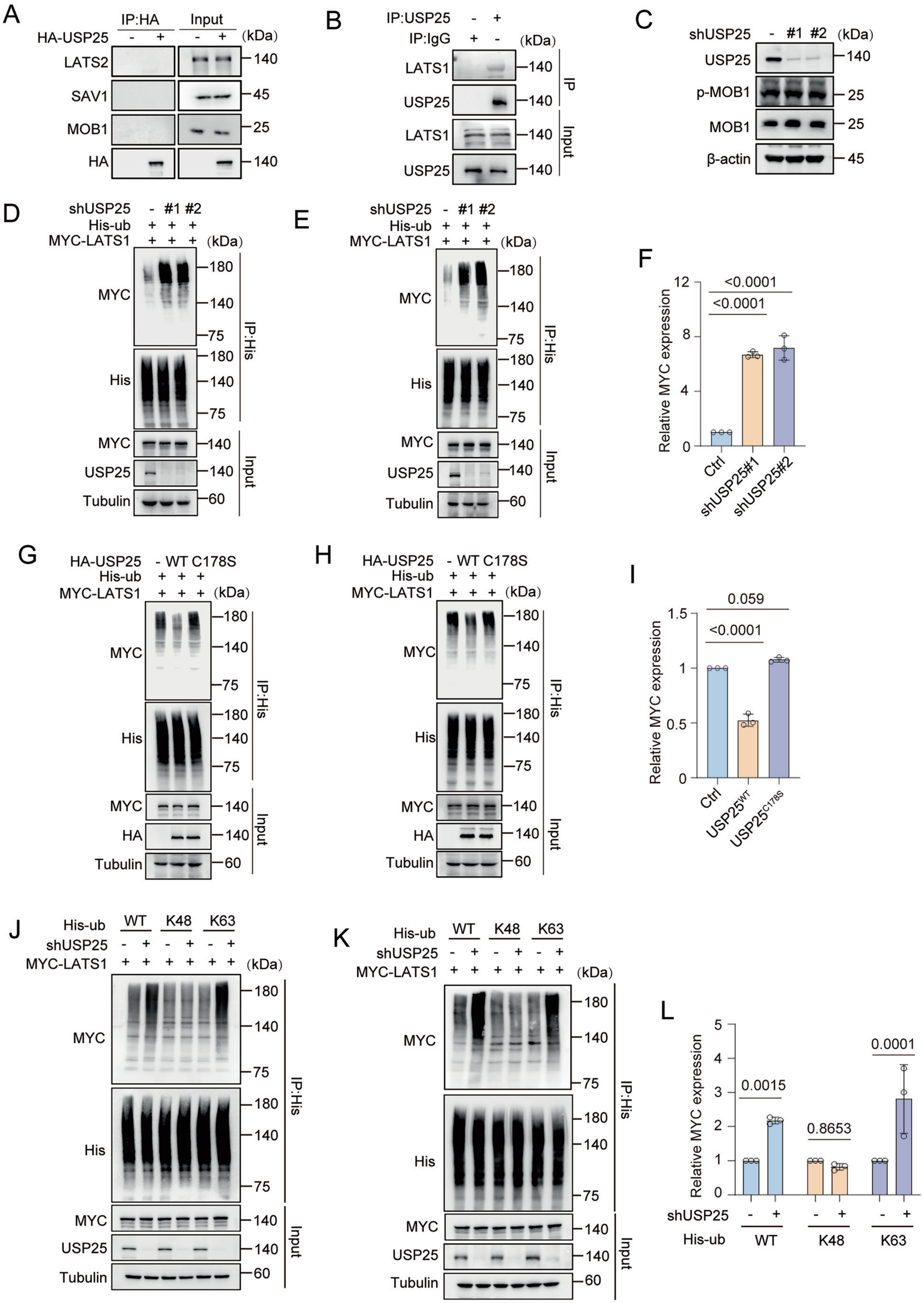
A
IP:HA
Input
HA-USP25 - + - + (kDa)
LATS2 140
SAV1 45
MOB1 25
HA 140
B
IP:USP25 - +
IP:IgG + - (kDa)
LATS1 140
USP25 140
IP
LATS1 140
USP25 140
Input
C
shUSP25 - #1 #2 (kDa)
USP25 140
p-MOB1 25
MOB1 25
β-actin 45
D
shUSP25 - #1 #2
His-ub + + +
MYC-LATS1 + + + (kDa)
MYC 180 140 75
His 180 140 75
IP:His
MYC 140
USP25 140
Tubulin 60
Input
E
shUSP25 - #1 #2
His-ub + + +
MYC-LATS1 + + + (kDa)
MYC 180 140 75
His 180 140 75
IP:His
MYC 140
USP25 140
Tubulin 60
Input
F
Relative MYC expression
12 8 4 0
<0.0001
<0.0001
Ctrl
shUSP25#1
shUSP25#2
G
HA-USP25 - WT C178S
His-ub + + +
MYC-LATS1 + + + (kDa)
MYC 180 140 75
His 180 140 75
IP:His
MYC 140
HA 140
Tubulin 60
Input
H
HA-USP25 - WT C178S
His-ub + + +
MYC-LATS1 + + + (kDa)
MYC 180 140 75
His 180 140 75
IP:His
MYC 140
HA 140
Tubulin 60
Input
I
Relative MYC expression
1.5 1 0.5 0
0.059
<0.0001
Ctrl
USP25WT
USP25C178S
J
His-ub WT K48 K63
shUSP25 - + - + - +
MYC-LATS1 + + + + + + (kDa)
MYC 180 140 75
His 180 140 75
IP:His
MYC 140
USP25 140
Tubulin 60
Input
K
His-ub WT K48 K63
shUSP25 - + - + - +
MYC-LATS1 + + + + + + (kDa)
MYC 180 140 75
His 180 140 75
IP:His
MYC 140
USP25 140
Tubulin 60
Input
L
Relative MYC expression
5 4 3 2 1 0
0.0015
0.8653
0.0001
shUSP25 - + - + - +
His-ub WT K48 K63

 © The Author(s)

Figure EV2. USP25 is a target of the Hippo pathway and deubiquitinates LATS1.

(**A**) HEK293T cells were transfected with control or HA-USP25 plasmids. Forty-eight hours after transfection, the cells were harvested. After HA immunoprecipitation, the blots were probed with the indicated antibodies. (**B**) Co-IP assay of the interaction between USP25 and LATS1 in HEK293T cells. Lysates from cells were prepared for co-IP experiments with an anti-USP25 antibody and then blotted with the indicated antibodies. (**C**) HEK293T cells were infected with lentivirus expressing control shRNA or USP25 shRNA. The blots were probed with the indicated antibodies. (**D–F**) USP25-knockdown HEK293T cells were transfected with the indicated plasmids. After His immunoprecipitation, the blots were probed with the indicated antibodies. MYC levels were quantified by densitometric analysis with ImageJ. The quantification results are presented ($n = 3$) (**F**). Ctrl vs. shUSP25#1 ($P =$ <0.0001) and Ctrl vs. shUSP25#2 ($P =$ <0.0001). (**G–I**) HEK293T cells were infected with $USP25^{WT}$ or $USP25^{C178S}$ lentiviral plasmids. After His immunoprecipitation, the blots were probed with the indicated antibodies. MYC levels were quantified by densitometric analysis with ImageJ. The quantification results are presented ($n = 3$) (**I**). Ctrl vs. $USP25^{WT}$ ($P < 0.0001$) and Ctrl vs. $USP25^{C178S}$ ($P = 0.059$). (**J–L**) His-Ub-lysine-specific mutant constructs were transfected into control or USP25-knockdown cells. Blots were probed with the indicated antibodies. MYC levels were quantified by densitometric analysis with ImageJ. The data are presented ($n = 3$) (**L**). WT, Ctrl vs. shUSP25 ($P = 0.0015$), K48, Ctrl vs. shUSP25 ($P = 0.8653$), and K63, Ctrl vs. shUSP25 ($P = 0.0001$). Statistical analysis was performed via one-way ANOVA (**F**, **I**) or two-way ANOVA (**L**) followed by Tukey's multiple comparison test. Source data are available online for this figure.

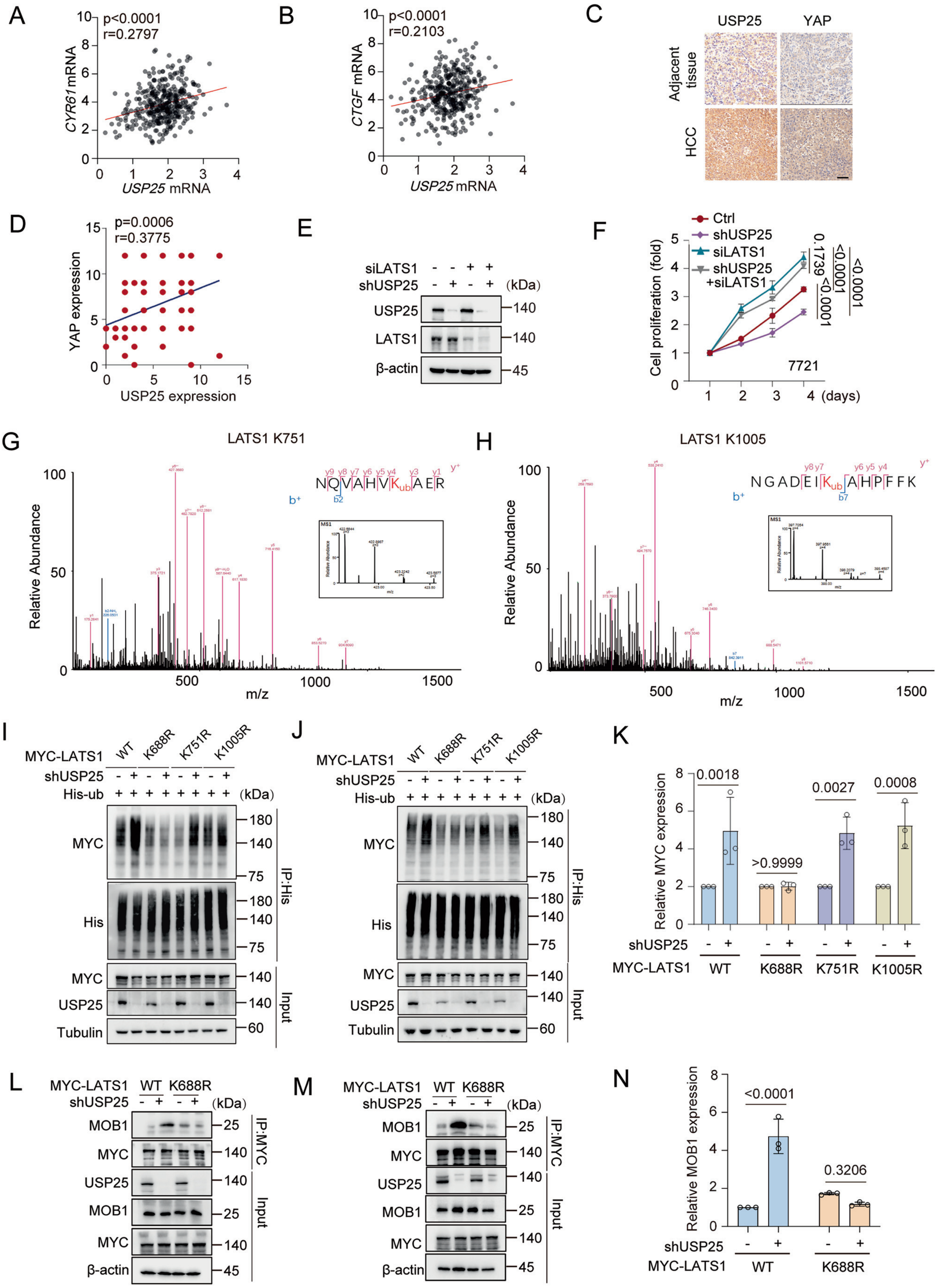
A
p<0.0001
r=0.2797
CYR61 mRNA
USP25 mRNA
B
p<0.0001
r=0.2103
CTGF mRNA
USP25 mRNA
C
USP25
YAP
Adjacent tissue
HCC
D
p=0.0006
r=0.3775
YAP expression
USP25 expression
E
siLATS1
shUSP25
(kDa)
USP25
LATS1
β-actin
140
140
45
F
Ctrl
shUSP25
siLATS1
shUSP25 +siLATS1
Cell proliferation (fold)
0.1739
<0.0001
<0.0001
<0.0001
7721
(days)
G
LATS1 K751
Relative Abundance
m/z
NQVAHVKubAER
H
LATS1 K1005
Relative Abundance
m/z
NGADEIKubAHPFFK
I
MYC-LATS1
WT
K688R
K751R
K1005R
shUSP25
His-ub
(kDa)
MYC
His
IP:His
MYC
USP25
Tubulin
Input
J
MYC-LATS1
WT
K688R
K751R
K1005R
shUSP25
His-ub
(kDa)
MYC
His
IP:His
MYC
USP25
Tubulin
Input
K
Relative MYC expression
0.0018
>0.9999
0.0027
0.0008
shUSP25
MYC-LATS1
WT
K688R
K751R
K1005R
L
MYC-LATS1
WT
K688R
shUSP25
(kDa)
MOB1
MYC
IP:MYC
USP25
MOB1
MYC
β-actin
Input
M
MYC-LATS1
WT
K688R
shUSP25
(kDa)
MOB1
MYC
IP:MYC
USP25
MOB1
MYC
β-actin
Input
N
Relative MOB1 expression
<0.0001
0.3206
shUSP25
MYC-LATS1
WT
K688R

 © The Author(s)

Figure EV3. USP25 deubiquitinates LATS1 at K688 to regulate its function.

(**A**, **B**) Correlation of USP25 mRNA expression with *CYR61* (**A**) and *CTGF* (**B**) mRNA expression in adjacent tissue ($n = 50$) and liver cancer samples ($n = 374$) according to a previously published microarray dataset. *CYR61* vs. *USP25* ($P < 0.0001$) and *CTGF* vs. *USP25* ($P < 0.0001$). (**C**, **D**) Tissue microarray with representative IHC staining images showing USP25 or YAP protein expression in HCC tissues. Representative images of the samples (**C**) and correlations between USP25 and YAP staining intensity (**D**). $n = 80$. Scale bars, 50 µm. *YAP* vs. *USP25* ($P = 0.0006$). (**E**, **F**) 7721 cells were infected or transfected with the indicated short hairpin RNA (shRNA) or short interfering RNA (siRNA) against USP25 or LATS1 and subjected to western blot (**E**). 7721 cell proliferation was quantified via a CCK-8 assay (**F**). Representative data (mean ± SD) are shown from 3 biologically independent samples. Ctrl vs. shUSP25 ($P < 0.0001$), Ctrl vs. siLAST1 ($P < 0.0001$), siLAST1 vs. shUSP25+siLAST1 ($P = 0.1739$), and Ctrl vs. shUSP25+siLAST1 ($P < 0.0001$). (**G**, **H**) MS spectra showing that LATS1 is deubiquitinated at the K751 residue (**G**) and K1005 residue (**H**). (**I–K**) Control or USP25-knockdown HEK293T cells were transfected with the indicated plasmids. After His immunoprecipitation, the blots were probed with the indicated antibodies. MYC levels were quantified by densitometric analysis with ImageJ. The quantification results are presented ($n = 3$) (**K**). WT, Ctrl vs. shUSP25 ($P = 0.0018$), K688R, Ctrl vs. shUSP25 ($P > 0.9999$), K751R, Ctrl vs. shUSP25 ($P = 0.0027$), and K1005R, Ctrl vs. shUSP25 ($P = 0.0008$). (**L–N**) Control or USP25-knockdown Hep3B cells were transfected with $LATS1^{WT}$ or the $LATS1^{K688R}$ mutant and subjected to western blot. MOB1 levels were quantified by densitometric analysis with ImageJ. The quantification results are presented ($n = 3$) (**N**). WT, Ctrl vs. shUSP25 ($P < 0.0001$) and K688R, Ctrl vs. shUSP25 ($P = 0.3206$). Statistical analysis was performed via two-way ANOVA (**F**, **K**, **N**) followed by Tukey's multiple comparison test. Source data are available online for this figure.

© The Author(s)

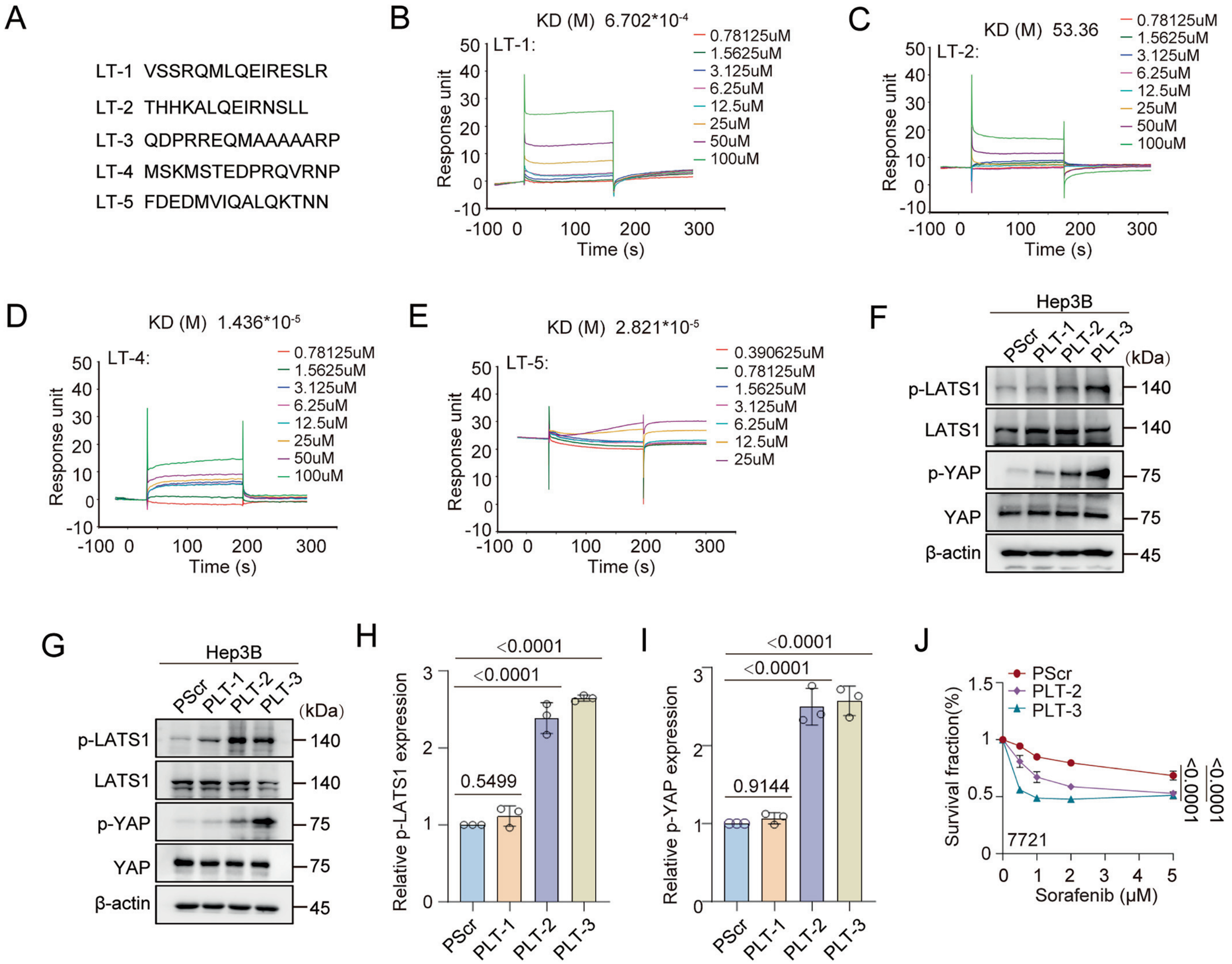

Figure EV4. Disruption of the interaction between USP25 and LATS1 suppresses liver cancer progression.

(**A**) Amino acid (aa) sequences of peptides that include the LATS1 binding region of USP25. (**B–E**) The kinetic interactions between the peptides and USP25 were assessed via surface plasmon resonance (SPR). (**F–I**) Hep3B cells were treated with the indicated peptides (40 μM) for 24 h. The protein expression of the indicated factors was assessed via western blotting. p-LATS1 and p-YAP levels were quantified by densitometric analysis with ImageJ. The quantification results are presented ($n = 3$) (**H, I**). p-LATS1, PScr vs. PLT-1 ($P = 0.5499$), PScr vs. PLT-2 ($P < 0.0001$), and PScr vs. PLT-3 ($P < 0.0001$). p-YAP, PScr vs. PLT-1 ($P = 0.9114$), PScr vs. PLT-2 ($P < 0.0001$), and PScr vs. PLT-3 ($P < 0.0001$). (**J**) Survival of 7721 cells in response to the indicated peptides (40 μM) combined with sorafenib (0, 0.5, 1, 2, or 5 μM) for 48 h determined via a CCK-8 assay. Representative data (mean ± SD) are shown from 3 biologically independent samples. PScr vs. PLT-2 ($P < 0.0001$) and PScr vs. PLT-3 ($P < 0.0001$). Statistical analysis was performed via one-way (**H, I**) or two-way (**J**) ANOVA followed by Tukey's multiple comparison test. Source data are available online for this figure.

 © The Author(s)

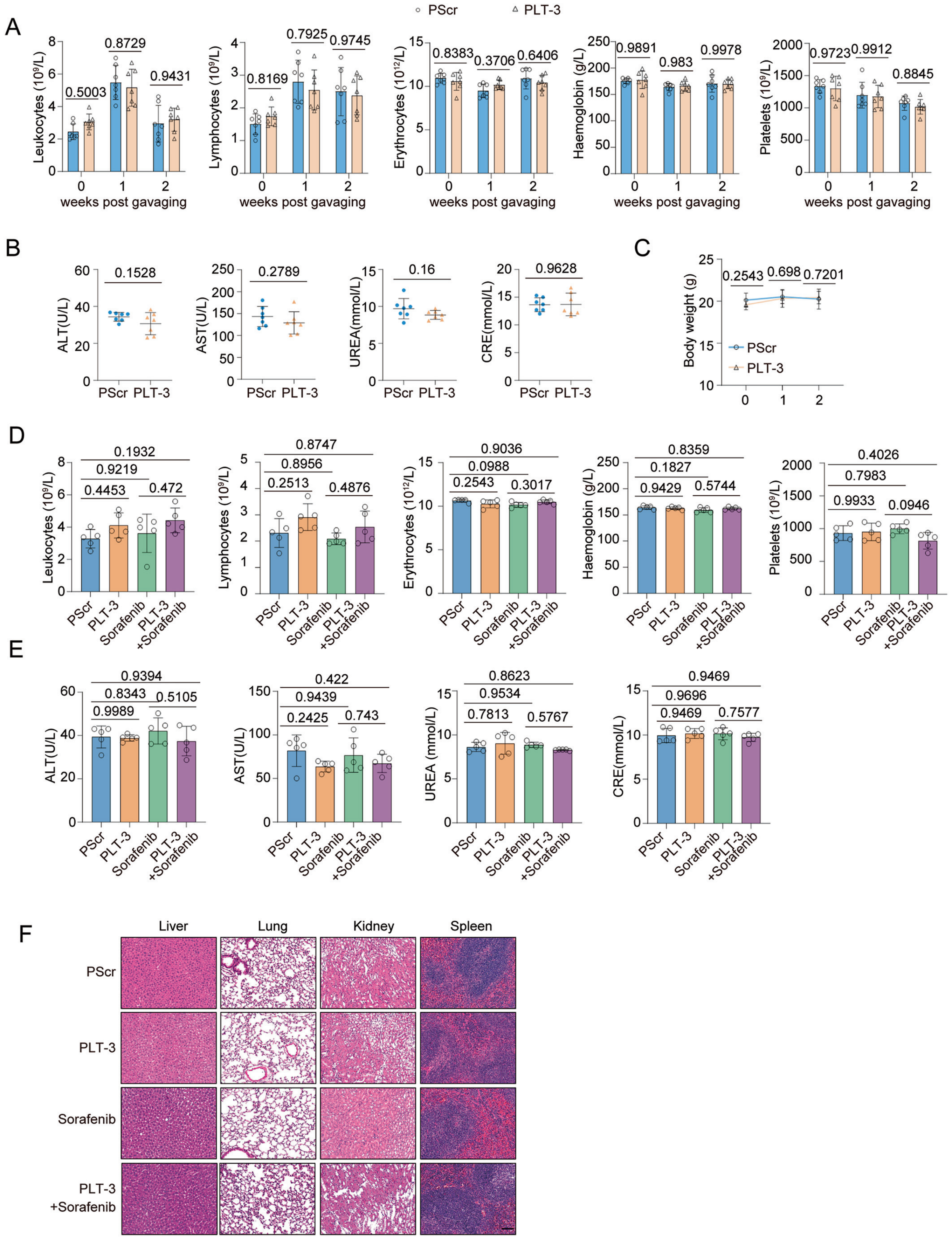
A
PScr
PLT-3
Leukocytes (10⁹/L)
Lymphocytes (10⁹/L)
Erythrocytes (10¹²/L)
Haemoglobin (g/L)
Platelets (10⁹/L)
weeks post gavaging
0.5003
0.8729
0.9431
0.8169
0.7925
0.9745
0.8383
0.3706
0.6406
0.9891
0.983
0.9978
0.9723
0.9912
0.8845
B
ALT(U/L)
AST(U/L)
UREA(mmol/L)
CRE(mmol/L)
0.1528
0.2789
0.16
0.9628
C
Body weight (g)
0.2543
0.698
0.7201
D
PScr
PLT-3
Sorafenib
PLT-3 +Sorafenib
0.1932
0.9219
0.4453
0.472
0.8747
0.8956
0.2513
0.4876
0.9036
0.0988
0.2543
0.3017
0.8359
0.1827
0.9429
0.5744
0.4026
0.7983
0.9933
0.0946
E
0.9394
0.8343
0.9989
0.5105
0.422
0.9439
0.2425
0.743
UREA (mmol/L)
0.8623
0.9534
0.7813
0.5767
0.9469
0.9696
0.9469
0.7577
F
Liver
Lung
Kidney
Spleen
PScr
PLT-3
Sorafenib
PLT-3
+Sorafenib

Figure EV5. The inhibitor peptide does not cause significant toxicity when used alone or in combination with sorafenib in PDX models.

(**A–C**) Routine blood indices (**A**), blood biochemical indices (**B**) and mouse body weight changes (**C**) are shown. Representative data (mean ± SD) are shown from 7 biologically independent samples. Leukocytes, PScr vs. PLT-3 (0w, $P = 0.5003$; 1w, $P = 0.8729$; 2w, $P = 0.9431$). Lymphocytes, PScr vs. PLT-3 (0w, $P = 0.8169$; 1w, $P = 0.7925$; 2w, $P = 0.9745$). Erythrocytes, PScr vs. PLT-3 (0w, $P = 0.8383$; 1w, $P = 0.3706$; 2w, $P = 0.6406$). Haemoglobin, PScr vs. PLT-3 (0w, $P = 0.9891$; 1w, $P = 0.983$; 2w, $P = 0.9978$). Platelets, PScr vs. PLT-3 (0w, $P = 0.9723$; 1w, $P = 0.9912$; 2w, $P = 0.8845$). ALT, PScr vs. PLT-3 ($P = 0.1528$). AST, PScr vs. PLT-3 ($P = 0.2789$). UREA, PScr vs. PLT-3 ($P = 0.16$). CRE, PScr vs. PLT-3 ($P = 0.9628$). Body weight, PScr vs. PLT-3 (0w, $P = 0.2543$; 1w, $P = 0.698$; 2w, $P = 0.7201$). (**D–F**) Routine blood indices (**D**), blood biochemical indices (**E**), and representative H&E staining images of different organs (**F**) are shown. Representative data (mean ± SD) are shown from 5 biologically independent samples. Scale bars, 200 µm. Leukocytes, PScr vs. PLT-3 ($P = 0.4453$), PScr vs. sorafenib ($P = 0.9219$), PScr vs. PLT-3+sorafenib ($P = 0.1932$), and sorafenib vs. PLT-3+sorafenib ($P = 0.472$). Lymphocytes, PScr vs. PLT-3 ($P = 0.2513$), PScr vs. sorafenib ($P = 0.8956$), PScr vs. PLT-3+sorafenib ($P = 0.8747$), and sorafenib vs. PLT-3+sorafenib ($P = 0.4876$). Erythrocytes, PScr vs. PLT-3 ($P = 0.2543$), PScr vs. sorafenib ($P = 0.0988$), PScr vs. PLT-3+sorafenib ($P = 0.9036$), and sorafenib vs. PLT-3+sorafenib ($P = 0.3017$). Haemoglobin, PScr vs. PLT-3 ($P = 0.9429$), PScr vs. sorafenib ($P = 0.1827$), PScr vs. PLT-3+sorafenib ($P = 0.8359$), and sorafenib vs. PLT-3+sorafenib ($P = 0.5744$). Platelets, PScr vs. PLT-3 ($P = 0.9933$), PScr vs. sorafenib ($P = 0.7983$), PScr vs. PLT-3+sorafenib ($P = 0.4026$), and sorafenib vs. PLT-3+sorafenib ($P = 0.0946$). ALT, PScr vs. PLT-3 ($P = 0.9989$), PScr vs. sorafenib ($P = 0.8343$), PScr vs. PLT-3+sorafenib ($P = 0.9394$), and sorafenib vs. PLT-3+sorafenib ($P = 0.5105$). AST, PScr vs. PLT-3 ($P = 0.2425$), PScr vs. sorafenib ($P = 0.9439$), PScr vs. PLT-3+sorafenib ($P = 0.422$), and sorafenib vs. PLT-3+sorafenib ($P = 0.743$). UREA, PScr vs. PLT-3 ($P = 0.7813$), PScr vs. sorafenib ($P = 0.9534$), PScr vs. PLT-3+sorafenib ($P = 0.8623$), and sorafenib vs. PLT-3+sorafenib ($P = 0.5767$). CRE, PScr vs. PLT-3 ($P = 0.9469$), PScr vs. sorafenib ($P = 0.9696$), PScr vs. PLT-3+sorafenib ($P = 0.9469$), and sorafenib vs. PLT-3+sorafenib ($P = 0.7577$). Statistical analysis was performed via *t* tests (**B**), one-way ANOVA (**D**, **E**) or two-way ANOVA (**A**) followed by Tukey's multiple comparison test. Source data are available online for this figure.

 © The Author(s)
