# Supplementary material for: USP25 aggravates liver cancer development and impairs chemosensitivity by limiting LATS1 activation
Source: EMBO Rep. 2026 Mar 25;27(9):2406–33. doi: 10.1038/s44319-026-00749-w (PMC13172046; doi:10.1038/s44319-026-00749-w)
Supplement: Supplementary file 2 — Source data Fig. 1 [file Western blots 1G.pdf › Figure 1/1G/Western blots 1G.pdf]

Figure 1G

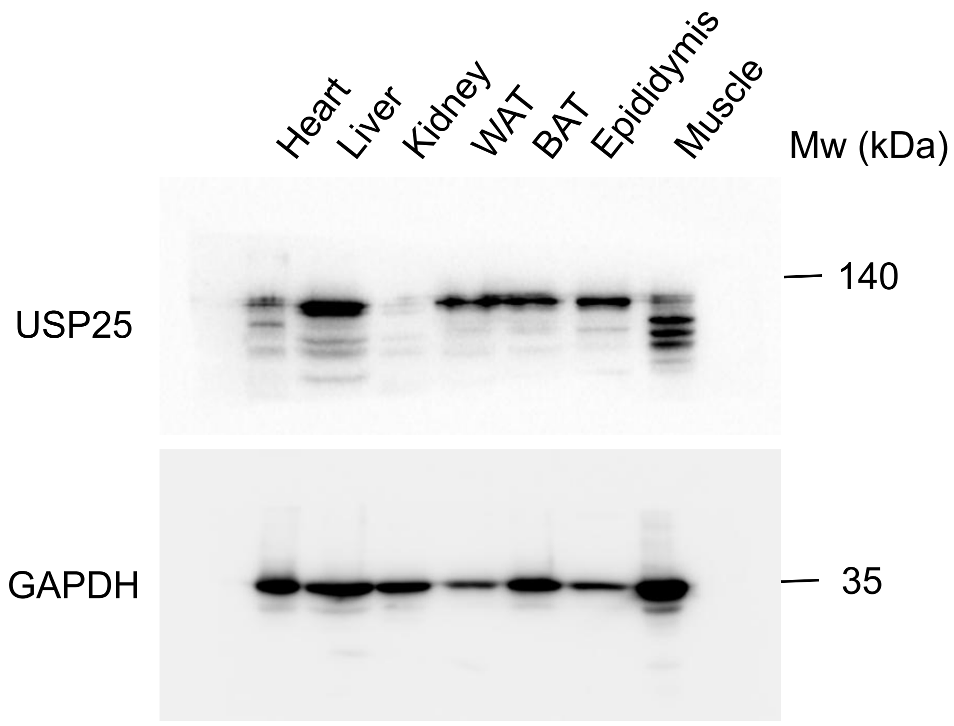

Heart
Liver
Kidney
WAT
BAT
Epididymis
Muscle
Mw (kDa)
USP25
140
GAPDH
35
