# Supplementary material for: USP25 aggravates liver cancer development and impairs chemosensitivity by limiting LATS1 activation
Source: EMBO Rep. 2026 Mar 25;27(9):2406–33. doi: 10.1038/s44319-026-00749-w (PMC13172046; doi:10.1038/s44319-026-00749-w)
Supplement: Supplementary file 11 — Figure EV3 Source Data [file 44319_2026_749_MOESM11_ESM.zip › Figure EV3/EV 3J/@@@2025-12-05 final point to point usp25 lats1.docx]

Authors response to reviewers’ comments

**Referee #1:**

Li et al. demonstrated that LATS1 activity is modulated by the regulation of its ubiquitination by the deubiquitinase USP25. They showed that decreased K688 ubiquitination of LATS1 due to USP25 overexpression reduces LATS1 phosphorylation, thereby enhancing YAP signaling. Conversely, increased LATS1 ubiquitination due to USP25 knockdown increases LATS1 phosphorylation and reduces YAP signaling. Overall, the authors proposed that reduced K688 ubiquitination of LATS1 due to high USP25 expression plays a positive role in hepatocellular carcinoma (HCC) progression. They developed a CPP that blocks the USP25-LATS1 interaction. They showed that the CPP increases p-YAP levels and enhances chemosensitivity in xenograft, patient-derived organoid, and PDX models. The authors conducted a large number of experiments, and the quality of the data is good in most cases.
However, although they provided novel findings showing that LATS1 ubiquitination regulates LATS1 activity without affecting its levels and demonstrating its clinical relevance, they never attempted to show whether this regulation is involved in Hippo signaling under normal conditions. The following questions are obvious and should be addressed:

*We are grateful for your expert review of our article. As you are concerned, there are several issues that need to be addressed. We have made revisions based on your valuable suggestions. The detailed corrections are listed below.*

Q1: What E3 ligase is responsible for the ubiquitination of K688 on LATS1?

*Thank you for your comment. The E3 ligase responsible for mediating ubiquitination at the K688 site of LATS1 is still elusive and we will perform further investigation to clarify it in future. Previous studies have demostrated three E3 liaases, CRL4^DCAF1^, Itch, and WWP1 regulate LATS1 protein stability. CRL4^DCAF1^ promotes the proteasomal degradation of LATS1 through ubiquitinating site at K830 on LATS1.While the specific ubiquitination sites of LATS1 modified by Itch and WWP1 remain unknown (PMID: 20178741; PMID: 21383157； PMID: 23573293).*

Q2: Is LATS1 ubiquitination regulated under typical Hippo signaling conditions? For example, are LATS1 ubiquitination levels on K688 low or high in low- or high-density cells, respectively?

*We performed the LATS1 ubiquitination assay under typical Hippo signaling conditions of high or low cell density and found that the ubiquitination of LATS1 WT markedly decreased under low cell density, while, the ubiquitination of LATS1 K688R mutant is lower and comparing in both high and low cell density (Below Figure 1).*

**Below Figure 1.** LATS1 ubiquitination levels on K688 in high- or low-density cells. HEK293 cells were seeded at 30% (low cell density) and 100% (high cell density) confluency for 16h and were transfected with indicated plasmids.

Q3: LATS1 and LATS2 share conserved domains, including the MOB1-binding domain. Why does USP25 interact only with LATS1 and not LATS2?

*Thanks for the comment. Human LATS1 and LATS2 are Ser/Thr kinases of the AGC subfamily, most closely related to the nuclear Dbf2-related kinases (NDR1/2). While LATS1 and LATS2 share extensive sequence similarity within their kinase domain (85% similarity) located at the C terminus of the proteins, the N terminus portion displays significantly lower conservation(Below Figure2) (PMID: 28644436). We identified the domains of LATS1 required for its interaction with USP25 and found the absence of truncation of the N-terminal of LATS1 completely abrogated its binding to USP25 (Figure. 6A-B). Collectively, the binding of USP25 to the N-terminus of LATS1, coupled with the low sequence conservation between the N-termini of LATS1 and LATS2, perhaps results in USP25 interacting specifically with LATS1 but not LATS2.*

**Below Figure 2** Schematic comparison of human LATS1 and LATS2 protein structures. The heatmap between the LATS1 and LATS2 protein schemes represents the similarity of the aligned sequences, where dark orange represents high and yellow represents low amino-acid similarity. Similarity was calculated using the Waterman Eggert local alignment application (EMBOSS explorer), comparing LATS1 (O95835-1) and LATS2 (Q9NRM7). Numbers above heatmap represent amino-acid position. Reported phosphorylation sites are designated as red lollipops, with the phosphorylating kinase indicated above in dark red. Ubiquitination sites are gray denoted by hexagons, with the reported E3 ligase indicated above in dark gray.

**Figure 6. (A)** Schematic structure of LATS1 truncation constructs. **(B)** Co-IP assay of the putative interaction between USP25 and the indicated LATS1 constructs. HEK293T cells were transfected with the indicated constructs of MYC-LATS1. Cell lysates were collected after 48h. MYC was immunoprecipitated, and the blots were then probed with the indicated antibodies.

Minor point:

Q4: The authors did not examine the interaction between MOB1 and LATS1 at endogenous levels.

*Thanks for the suggestion. We did the suggested experiment and showed the interaction between MOB1 and LATS1 at endogenous levels in Hep3B cells (Below Figure 3).*

**Below Figure 3.** The interaction between MOB1 and LATS1 at endogenous levels in Hep3B cells. Lysates from cells were prepared for co-IP experiments with LATS1 antibody and then blotted with the indicated antibodies.

**Referee #2:**

In this manuscript, Li et al. found that USP25 is overexpressed in HCC, and can mediate LATS1 deubiquitination, which inhibits LATS1 activation and functions as the YAP kinase. They showed that depletion of USP25 significantly suppresses HCC cell growth and tumor growth. Based on this, they developed a peptide that can disrupt USP25-LATS1 interaction, and synergizes with chemotherapy in xenograft, patient-derived organoid and PDX models. Overall, this study is interesting. However, several issues need to be addressed before further consideration.

*We are grateful for your expert review of our article. As you are concerned, there are several issues that need to be addressed. We have made revisions based on your valuable suggestions. The detailed corrections are listed below.*

Q1: In Figure 1D-E, what is the age of the mice used? Why does normal mouse liver show such a high rate of Ki67^+^ cells?

*Thanks for the comment. In Figure 1D, 2 months of age mice was used. Generally, the proliferation rate of Ki67^+^ cells in the livers of young mice is between 1-3%. In Figure 1E, we quantified that the number of Ki67^+^ cells in the fields, not the rate of Ki67^+^ cells. We measured the proliferation rate of Ki67^+^ cells in the livers of normal young mice and found it to be approximately 2.2%. We have updated figure to show the proliferation rate of Ki67^+^ cells (new Figure 1E).*

**Figure1 D-E.** Representative micrographs (D) and quantification data (E) of Ki-67 (green) are shown for Usp25^+/+^ and Usp25^-/-^ mouse livers. Livers from 2-month-old mice. Scale bars, 50 µm.

Q2: In Figure 1J, why do stage IV tumors show lower levels of USP25 than stage III tumors? The *p*-value refers to which comparison?

*Thanks for the comment. In Figure 1J, USP25 expression of LIHC patients with different stages (TCGA samples) were analyzed in GEPIA databases. The p-value displayed in the GEPIA Pathological Stage Plot is calculated using the Kruskal-Wallis H test. This test is a non-parametric method used to determine whether there is an overall significant difference in the medians among multiple independent groups (different pathological stages). This p-value measures the likelihood that the expression differences between groups are due to random sampling error. It does not directly indicate which specific two groups differ from each other (Below Figure 4A).*

*Furthermore, we analyzed USP25 expression of LIHC patients with different stages (TCGA samples) through Ualcan database. p-value directly indicate which specific two groups differ from each other. The analysis from this database included pairwise comparisons of the data and provided the corresponding p-values. These results were shown the high USP25 expression correlates with tumor Stage 1-3 compared with normal tissue, but no significant differences on Stage 4. This might be due to the small number of samples in Stage 4 (only 6 cases)(Below Figure4 B-C). We have updated figure and annotated it with the sample size and p-value (new Figure 1J).*

C

A

B

**Below Figure4.** USP25 expression of LIHC patients with different stages (TCGA samples) were analyzed in GEPIA databases (A) or Ualcan databases (B-C).

**Figure 1J.** USP25 expression of patients with different stages in Ualcan database.

Q3: Does USP25 also bind to and regulate LATS2, or is this regulation unique to LATS1?

*Thanks for the comment. We analyzed the interaction between USP25 with LATS2 by co-IP assay in Figure EV2A. The results showed that USP25 has no interaction with LATS2. We further checked whether USP25 deubiquitinates LATS2 and found that USP25 depletion didn’t promoted the ubiquitination of LATS2 (Below Figure 4).*

**Below Figure 4.** USP25-knockdown HEK293T cells were transfected with the indicated plasmids. His was immunoprecipitated, and the blots were then probed with the indicated antibodies.

**Fig EV2A.** HEK293T cells were transfected with HA-USP25. HA was immunoprecipitated and the blots were probed with the indicated antibodies.

Q4: In Figure 3L, it seems that myc-LATS1 showed increased HA-Ub signal in shUSP25 cells even without adding HA-Ub. How could this happen? Why was there an equal amount of HA-Ub in the input, even without adding HA-Ub?

*Thank you for this suggestion. In this experiment, HA-Ub wild type, HA-Ub K48 and HA-Ub K63 plasmids were transfected into the ctrl or shUSP25 cells, respectively. MYC was immunoprecipitated, and the blots were then probed with the indicated antibodies. Following your suggestion, we have made the corrections (Figure 3L).*

**Figure 3L.** USP25-knockdown HEK293T cells were transfected with the indicated plasmids. MYC was immunoprecipitated, and the blots were then probed with the indicated antibodies.

Q5: Based on the model, LATS1 ubiquitination at K688 will facilitate its interaction with MOB1. Can the authors provide some explanation of how this could happen?

*Thanks for the comment. Recent structural studies have shown the importance of MOB1 proteins as kinase adaptors in the Hippo pathway. MST1/2 kinases recruit and phosphorylate MOB1, which subsequently interacts with and activates LATS1 kinases, leading to YAP phosphorylation and cytoplasmic retention* (PMID: 16674920; PMID: 16096061; PMID: 26108669)*. Our results found that USP25 knockdown promoted LATS1 ubiquitiantion, which in turn increased the interaction between MOB1 and LATS1(Figure 3J), while, LATS1 K688R mutant abolished USP25 mediated ubiquitination signaling dramatically decreased its interaction with MOB1(Figure 4G). We speculated that ubiquitin-modified LATS1 may undergo structural changes and promoted the interaction between MOB1 and LATS1. In the future, we will performed structural analyse to verify the LATS1 structure changed after ub-modification at the K688 and the effect on the interaction between LATS1 and MOB1.*

**Figure 3J.** USP25-knockdown HEK293T cells were transfected with the indicated plasmids. His was immunoprecipitated, and the blots were then probed with the indicated antibodies; **Figure 4G.**  USP25-knockdown cells were transfected with MYC-LATS1WT or MYC-LATS1K688R plasmids. Forty-eight hours after transfection, cells were harvested. MYC was immunoprecipitated, and the blots were then probed with the indicated antibodies.

Q6: In Figure 6A, it is shown that FL and F1 bind to USP25, but F2 does not. Since F2 contains the region of F1, how to explain the result that F2 can not bind to USP25?

*Thanks for the comment. Following your suggestion, we repeated to identify the domain of LATS1 required for its interaction with USP25. The results were shown that FL, F1（1-160bp） and F2 （1-589bp）bind to USP25 in long exposure, but the band, that F2（1-589bp）bind to USP25, is a little bit weak. Furthermore, we checked weather F3 (new, 161-589bp) bind to USP25 and found that F3 couldn’t bind to USP25 (Figure 6A-B). These results suggested that the F2 (1-160bp) fragment of LATS1 is likely the minimal region capable of binding to USP25.*

**Figure 6A-B. To identify the domain of LATS1 required for its interaction with USP25.** (A) Schematic structure of LATS1 truncation constructs. (B) Co-IP assay of the putative interaction between USP25 and the indicated LATS1 constructs. HEK293T cells were transfected with the indicated constructs of MYC-LATS1. Cell lysates were collected after 48h. MYC was immunoprecipitated, and the blots were then probed with the indicated antibodies.

Q7: As shown in Figure 6D, the PLT-3 peptide can disrupt LATS1-USP25 binding. The authors should also investigate whether PLT-3 can enhance the ubiquitination of LATS1 and whether it can increase the interaction between LATS1 and MOB1.

*Thanks for the insightful comments. We tested the ubiquitination of LATS1 and the interaction between LATS1 and MOB1 upon treatment with PLT-1, PLT-2 or PLT-3, and found that PLT-3 can notably increase the interaction between LATS1 and MOB1(new, Figure 6E) and stimulated the ubiquitination of LATS1(new, Figure. 6F).*

**Figure 6E.** HEK293T cells were transfected with the MYC-LATS1 plasmid and treated with the indicated peptides (40µM) for 24 h. MYC was immunoprecipitated, and the blots were then probed with the indicated antibodies. **Figure 6F.** HEK293T cells were transfected with the indicated plasmids and treated with the indicated peptides (40µM) for 24 h. His was immunoprecipitated, and the blots were then probed with the indicated antibodies.

Q8: Since the study has focused on HCC, the author did not provide a rationale for the use of colon cancer PDXs in Figure 7E-F, but not HCC PDXs.

*Thanks for careful reading. That was a typo. We make the corrections on page 7. We performed HCC PDXs but not the colon cancer PDXs by examining the anti-tumor effect of PLT-3. First, we screened three liver cancer PDXs by examining their USP25 levels through the western blot and IHC analyses. As shown in Fig. 7E-F, the tumor from patient 2 exhibited the highest USP25 protein level.*

**Figure 7E.** Western blots of three HCC patient tumors (patients 1-3) showing USP25 levels were presented. **Figure 7F.** Representative HE and immunohistochemistry (IHC) micrographs showing USP25 expression in HCC patient-derived tumors were provided. Scale bars, 100 µm.

Q9: It was known that USP25 can activate several oncogenes other than YAP. It is plausible that when USP25 is inhibited or depleted, these oncogenes would be inhibited. The author should examine whether these potential effects could account for the tumor suppression effect when USP25 is inhibited.

*Thanks for the comment. Recent studies have shown KIFC or Tankyrases (TNKS), which was deubiquitinated and stablized by USP25, displayed oncogenes and multi-drug resistance in Cervical Squamous Cell Carcinoma or Colorectal Carcinoma (PMID: 40379626;PMID: 38261825). To exam whether besides YAP, other USP25 substrates also contribute to USP25 mediated HCC proliferation and therapeutic resistance, we generated USP25 and KIFC or TNKS single- and double-knockdown Hep3B and 7721 HCC cell lines. CCK-8 assays revealed that USP25 knockdown but not KIFC or TNKS knockdown reduced cell proliferation and led cells sensitize to sorafenib, while double-knockdown cells did not further affect cell proliferation or sorafenib response compare to USP25 knockdown cells (Below Figure. 5A-5L). However, knockdown of YAP dramatically decreased cell proliferation and led cells sensitize to sorafenib. In YAP depleted cells, knockdown of USP25 cannot further reduced cell proliferation.*

*Collectively, these results demonstrated that USP25 regulates liver cancer cell proliferation and chemotherapeutic sensitivity majorly through YAP*

**Below Figure 5. USP25 regulates liver cancer cell proliferation and chemotherapeutic sensitivity in a KIFC or TNKS-independent manner.**  Immunoblotting of USP25 in control, USP25-knockdown, KIFC-knockdown and double-knockdown Hep3B cells (5A) or 7721 cells (5G). Immunoblotting of USP25 in control, USP25-knockdown, TNKS-knockdown and double-knockdown Hep3B cells (5D) or 7721 cells (5J). The proliferation in control, USP25-knockdown, KIFC-knockdown and double-knockdown Hep3B cells (5B) or 7721 cells (5H) was quantified via a CCK-8 assay. The proliferation in control, USP25-knockdown, TNKS-knockdown and double-knockdown Hep3B cells (5E) or 7721 cells (5K) was quantified via a CCK-8 assay. Survival assays for the CCK-8 assay in control, USP25-knockdown, KIFC-knockdown and double-knockdown Hep3B cells (5C) or 7721 cells (5I) after sorafenib treatment. Survival assays for the CCK-8 assay in control, USP25-knockdown, TNKS-knockdown and double-knockdown Hep3B cells (5F) or 7721 cells (5L) after sorafenib treatment. Statistical analysis was performed via two-way ANOVA followed by Tukey’s multiple comparison test.

**Referee #3:**

The study by Li et al presents data suggesting a function for the USP25 deubiquitinating enzyme in the regulation of the LATS1 kinase of the Hippo signaling pathway. USP25 knockout mice were found to have smaller livers and high USP25 levels were identified to be associated with hepatocellular carcinomas and promote cell growth in vitro. This prompted the authors to investigate associated mechanisms, which led to the finding that USP25 interacts with the LATS1 kinase. The study argues that depletion of USP25 leads to increased K63-Ubiquitnation of LATS1 and that this modification promotes association with MOB1 to increase LATS1 phosphorylation and subsequent phosphorylation of the transcriptional regulator YAP. A cell permeable peptide that reduces USP25 interaction with LATS1 was identified to function in a similar manner to USP25 depletion, blocking liver cancer cell line and patient-derived organoid growth in vitro and reducing liver cancer cell line xenograft growth in vivo. Further, USP25 depletion/inhibition and the identified peptide inhibitor were found to enhance liver cancer cell sensitivity to the chemotherapeutic sorafenib, suggesting that the USP25-LATS1 axis may offer a new target for cancer therapy. Collectively, the study identifies new functions for USP25 and offers insight into how the ubiquitin pathway may influence Hippo pathway activity in liver cancers. While interesting, however, there are several weaknesses in the data and conclusions that are made, which are summarized in the comments below.

*We are grateful for your expert review of our article. As you are concerned, there are several issues that need to be addressed. We have made revisions based on your valuable suggestions. The detailed corrections are listed below.*

Q1: In general, conclusions from the western blot experiments should be quantified over several replicates.

*Thanks for the comment. We have repeated some key experiments, including the deubiquitination assay (Figure. 3J,K,L, Figure. 4B), the interaction between LATS1WT/K688R and MOB1 (Figure. 4G), and the expression of phospho-LAST1 or phospho-YAP upon treatment with peptides (Figure. 6H). The results were repeatable (Figure EV2D-E, EV2G-H, EV2J-K, EV3I-J, EV3L-M, EV4F-G). Furthermore, we quantified these replicates (Figure EV2F, EV2I, EV2L, EV3K, EV3N, EV4H-I).*

**Figure3J, EV2D-F** USP25-knockdown HEK293T cells were transfected with the indicated plasmids. His was immunoprecipitated, and the blots were then probed with the indicated antibodies. MYC level were quantified with the analysis in densitometric analysis in Image J. The quantifications were presented (F).

**Figure 3K, EV2G-I** Control or USP25-knockdown cells were infected with USP25^WT^ or the USP25^C178S^ lentiviral plasmids. His was immunoprecipitated, and the blots were then probed with the indicated antibodies. MYC level were quantified with the analysis in densitometric analysis in Image J. The quantifications were presented (I).

**Figure 3L, EV2J-L** His-Ublysine-specific mutant constructs were transfected into control or USP25 knockdown cells. Blots were probed with the indicated antibodies. His was immunoprecipitated, and the blots were then probed with the indicated antibodies. MYC level were quantified with the analysis in densitometric analysis in Image J. The quantifications were presented (L).

**Figure 4B, EV3I-K** Control or USP25-knockdown HEK293T cells were transfected with the indicated plasmids. His was immunoprecipitated, and the blots were then probed with the indicated antibodies. MYC level were quantified with the analysis in densitometric analysis in Image J. The quantifications were presented (K).

**Figure 4G, EV3L-N** USP25-knockdown cells were transfected with MYC-LATS1^WT^ or MYC-LATS1^K688R^ plasmids. Forty-eight hours after transfection, cells were harvested. MYC was immunoprecipitated, and the blots were then probed with the indicated antibodies. MOB1 level were quantified with the analysis in densitometric analysis in Image J. The quantifications were presented (N).

**Figure 6H, EV4F-I** Hep3B cells were treated with the indicated peptides (40 µM) for 24 h. The protein expression of the indicated genes was assessed via western blot. p-LATS1 (H) or p-YAP (I) level were quantified with the analysis in densitometric analysis in Image J. The quantifications were presented.

Q2: Unclear if the data shown in 1J is consistent with the conclusion that "high USP25 expression correlates with advanced BCLC stage". Stage IV appears to show lowest expression.

*Thanks for the comment. In Figure 1J, USP25 expression of LIHC patients with different stages were analyzed in GEPIA databases. The p-value displayed in the GEPIA Pathological Stage Plot is calculated using the Kruskal-Wallis H test. This test is a non-parametric method used to determine whether there is an overall significant difference in the medians among multiple independent groups. It does not directly indicate which specific two groups differ from each other (Below Figure3 A).*

*Furthermore, we analyzed USP25 expression of LIHC patients with different stages (TCGA samples) through Ualcan database. p-value directly indicate which specific two groups differ from each other. The analysis from this database included pairwise comparisons of the data and provided the corresponding p-values. These results were shown the high USP25 expression correlates with tumor Stage 1-3 compared with normal tissue, but no significant differences on Stage 4 (Below Figure3 B-C). Due to the limited sample size of Stage 4 (only 6 cases), which may not provide a statistically reliable representation, it was excluded from the subsequent data visualization. We have updated figure (new Figure 1J) and annotated it with the sample size and p-value.*

C

A

B

**Below Figure3.** USP25 expression of LIHC patients with different stages (TCGA samples) were analyzed in GEPIA databases (A) or Ualcan databases (B-C).

**Figure 1J.** USP25 expression of patients with different stages in Ualcan database.

Q3: Unclear if the data in 2A is consistent with the conclusion "the expression levels of USP25 were greater in multiple liver cancer cell lines than in the normal liver cell line LO2". The differences appear visually very minor (no quantitation is included) and the Huh7 cancer cell line appears to have the lowest USP25 levels.

*Thanks for the comment. To make the description more accurate, we changed the conclusion in the manuscript as follows:*

*To clarify the role of USP25 in hepatocarcinoma, we measured the protein level of USP25 in liver cancer cell lines through western blot and the results revealed that the expression levels of USP25 were higher in multiple liver cancer cell lines (HepG2, 7721 and Hep3B) but lower in Huh7 and in the normal liver cell line LO2 (Figure 2A). The results were repeatable and quantified these replicates (Figure EV1 E-G).*

**Figure 2A, EV1E-G.** The protein level of USP25 in liver cancer cell lines and normal liver cell line via WB blot. USP25 level were quantified with the analysis in densitometric analysis in Image J. The quantifications were presented (G).

Q4: The manuscript would benefit from including all the results from the IP-MS data presented in Figure 3A, along with making this data accessible to the public. Additionally, it is unclear how this data was generated. No details are provided in the methods section and it is unclear if any controls were used for defining an interaction.

*Thanks for pointing this out. The generation of MS data of LATS1 ubiquitination sites is detailed in methods section. To identify interacting protein of USP25, HEK293T cells (Control) or stably expressing HA-USP25 were lysed and purified using anti-HA-agarose beads. These samples separate on SDS-PAGE and stain with Coomassie blue. After staining with Coomassie Brilliant Blue, the target protein bands were excised and destained. Next, the gel bands were dehydrated and rehydrated in 10 mM dithiothreitol（DTT）and incubated at 56℃ for 60 mins. Then followed by alkylation with 55 mM iodoacetamide (IAA) in the dark at room temperature for 30 mins. The supernatant was then removed, and the gel pieces were dried. Then the gel pieces were rehydrated in a trypsin solution (10 ng/μL) and incubated overnight at 37℃. After digestion, the peptides remaining in the gel pieces were further extracted. The combined supernatants were dried and were desalted using C18 Zip-Tips. The peptides were subsequently detected using Nano-LC−MS/MS analysis and the mass spectrometry raw data were analyzed using the Mascot search engine (version 2.3.01) against the UniProt Homo sapiens proteome database.*

*The original mass spectrometry data and the database search results have been deposited to the ProteomeXchange Consortium via the iProX partner with the project ID IPX0012796001*

*(URL: https://www.iprox.cn/page/SSV024.html;url=17537021143526VHi, Password: nrGJ).*

Q5: The images in 3G are not convincing. It would be helpful to include a more zoomed out images that include many cells (ideally with cells of similar confluence).

*Thanks for the comment. We have now changed images that include more cells than before (Figure. 3G). These results showed that USP25 deficiency significantly reduced YAP nuclear translocation in Usp25-/- MEFs (Figure. 3H).*

**Figure 3G-H.** USP25 depletion decreased YAP nuclear localization in *Usp25*^+/+^ and *Usp25*^-/-^ MEFs. MEFs were subjected to immunofluorescence staining **(G)** and quantitative analysis **(H)**. When N<C, YAP is enriched in the cytoplasm; when N=C, YAP is evenly distributed in the cytoplasm and nucleus; and when N>C, YAP is enriched in the nucleus. Scale bars, 5 μm.

Q6: The data in 3J (and also 3K) is not convincing. Quantitation across replicates would strengthen this data. It is also unclear that what is shown in the top IP is actually ubiquitinated LATS1 since controls are not included. The Ub blot is cut off at 130kDa for all the experiments and it appears the smear goes down to a much lower size, raising questions into what is actually being visualized since the IP of LATS1 seems to run at near 180kDa.

*Thanks for the comment. The LATS1 protein, which is 1131 amino acids in length, has an apparent molecular weight of approximately 140 kDa. We have corrected the molecular weight marker indications for LATS1 in all relevant figures.*

*Under non-denaturing conditions, ubiquitinated proteins may form non-covalent associations with numerous ubiquitin-binding proteins through ubiquitin chains or other protein intermediaries. These interactions can significantly complicate the composition of immunoprecipitated products, potentially introducing non-specific interacting proteins and resulting in non-specific background or high-molecular-weight aggregated bands/smearing during Western blot analysis. Because we performed deubqutination assay using undenaturing conditions, it perhaphs appears the smear goes down to a much lower size.*

*Futhermore, denaturing conditions disrupt all these non-covalent interactions, preserving only the covalent ubiquitin-substrate linkages. This approach significantly enhances the specificity of immunoprecipitation, ensuring that the detected signals more directly reflect the true ubiquitination levels with cleaner background and sharper bands. We repeated the experiments presented in Figure. 3J,K using denaturing conditions in independently repeated three times, followed by quantitative analysis (Figure. EV2D-I). Furthermore, deubiquitination assay under denaturing conditions was added methods section.*

**Figure3J, EV2D-F** USP25-knockdown HEK293T cells were transfected with the indicated plasmids. His was immunoprecipitated, and the blots were then probed with the indicated antibodies. MYC level were quantified with the analysis in densitometric analysis in Image J. The quantifications were presented (F).

**Figure 3K, EV2G-I** Control or USP25-knockdown cells were infected with USP25^WT^ or the USP25^C178S^ lentiviral plasmids. His was immunoprecipitated, and the blots were then probed with the indicated antibodies. MYC level were quantified with the analysis in densitometric analysis in Image J. The quantifications were presented (I).

Q7: It is very unclear how conclusions can be made from Figure 3L, since HA staining is showing up in the supposed control lane. Similar concerns for this blot as the comment above given that LATS1 runs at ~180kDa.

*Thanks for the suggestion. We have repeated this experiment under denaturing conditions in independently repeated three times, followed by quantitative analysis. Very similar results were obtained (Figure3L; Fig. EV2J-L). Furthermore, we quantified these replicates.*

**Figure 3L, EV2J-L** His-Ublysine-specific mutant constructs were transfected into control or USP25 knockdown cells. Blots were probed with the indicated antibodies. His was immunoprecipitated, and the blots were then probed with the indicated antibodies. MYC level were quantified with the analysis in densitometric analysis in Image J. The quantifications were presented (L).

Q8: No method details are provided for how mass spectrometry was performed to analyze LATS1 ubiquitination sites, and this data does not appear to be shared in any way.

*Thanks for pointing this out. The generation of MS data of LATS1 ubiquitination sites is detailed in the methods section. To identify LATS1 deubiquitination site, control and USP25 knockdown cells stably expressing His-ub and MYC-LATS1. These samples separate on SDS-PAGE and stain with Coomassie blue. Cut off the spectral band corresponding to MYC-LATS1. After staining with Coomassie Brilliant Blue, the target protein bands were excised and destained. Next, the gel bands were dehydrated and rehydrated in 10 mM dithiothreitol（DTT）and incubated at 56℃ for 60 mins. Then followed by alkylation with 55 mM iodoacetamide (IAA) in the dark at room temperature for 30 mins. The supernatant was then removed, and the gel pieces were dried. Then the gel pieces were rehydrated in a trypsin solution (10 ng/μL) and incubated overnight at 37℃. After digestion, the peptides remaining in the gel pieces were further extracted. The combined supernatants were dried and were desalted using C18 Zip-Tips. The peptides were subsequently detected using Nano-LC−MS/MS analysis and the mass spectrometry raw data were analyzed using the Mascot search engine (version 2.3.01) against the UniProt Homo sapiens proteome database.*

*The original mass spectrometry data and the database search results have been deposited to the ProteomeXchange Consortium via the iProX partner with the project ID IPX0012796001*

*(URL: https://www.iprox.cn/page/SSV024.html;url=17537021143526VHi, Password: nrGJ).*

Q9: Based on the model and conclusions presented, the K688R mutant of Lats1 would be less phosphorylated given that would be unable to interact with Mob1. Is this the case?

*Yes, it is. Our results that USP25 knockdown increased LATS1 and YAP phosphorylation in the LATS1^WT^ cells but not in the LATS1^K688R^ mutant cells (Figure.4D). USP25 knockdown promoted the interaction between MOB1 and LATS1, whereas LATS1 K688R impaired this interaction (Figure.4F-G). Collectively, we hypothesize that the K688R mutant of Lats1 would be less phosphorylated due to its inability to interact with Mob1.*

**Figure 4D,F,G.** USP25 deubiquitinates LATS1 and regulates its function at K688. **(D)** Immunoblotting analysis of the indicated protein levels in USP25-knockdown cells expressing the MYC-LATS1^WT^ or the MYC-LATS1^K688R^ mutant via western blot. Cell lysates were collected after 48h and the blots were then probed with the indicated antibodies. **(F)** Control or USP25-knockdown cells were transfected with the MYC-LATS1^WT^ plasmid. Forty-eight hours after transfection, cells were harvested. MYC was immunoprecipitated, and the blots were then probed with the indicated antibodies. **(G)** USP25-knockdown cells were transfected with MYC-LATS1^WT^ or MYC-LATS1^K688R^ plasmids. Forty-eight hours after transfection, cells were harvested. MYC was immunoprecipitated, and the blots were then probed with the indicated antibodies.

Minor comment:

Q10: While the interaction between Lats1 and USP25 is convincing based on Fig C-E, it is unclear why the authors chose to test the endogenous interaction in HEK293 cells given that the focus on hepatocellular carcinoma and the use of the liver cancer cell lines and organoids for functional experiments. Endogenous interactions in these cells would benefit the conclusions of the manuscript.

*Thanks for the suggestion. We investigated whether LATS1 bind directly with USP25 by endogenous co-immunoprecipitation (Co-IP) analysis in Hep3B cells. As expected, USP25 specifically interacted with LATS1 (new Figure. 3E).*

**Figure 3E** USP25 specifically interacted with LATS1 by co-IP endogenous co-IP assays in Hep3B cells*.* Co-IP assay of the interaction between USP25 and LATS1 using antibodies to USP25 in Hep3B cells. Lysates from cells were prepared for co-IP experiments with USP25 antibody and then blotted with the indicated antibodies.
